# The putative maintaining mechanism of gut bacterial ecosystem in giant pandas and its potential application in conservation

**DOI:** 10.1111/eva.13494

**Published:** 2022-12-30

**Authors:** Xinyuan Cui, Qinrong Zhang, Qunde Zhang, Hua Chen, Guoqi Liu, Lifeng Zhu

**Affiliations:** ^1^ College of Life Sciences Nanjing Normal University Nanjing China; ^2^ Mingke Biotechnology (Hangzhou) Co., Ltd. Hangzhou China

**Keywords:** gut microbiome, keystone species, maintaining mechanism, module analysis, rare taxa, wild and captive giant panda

## Abstract

Animals living in captivity and the wild show differences in the internal structure of their gut microbiomes. Here, we performed a meta‐analysis of the microbial data of about 494 fecal samples obtained from giant pandas (captive and wild giant pandas). Our results show that the modular structures and topological features of the captive giant panda gut microbiome differ from those of the wild populations. The co‐occurrence network of wild giant pandas also contained more nodes and edges, indicating a higher complexity and stability compared to that of captive giant pandas. Keystone species analysis revealed the differences between geographically different wild populations, indicating the potential effect of geography on the internal modular structure. When combining all the giant panda samples for module analysis, we found that the abundant taxa (e.g., belonged to *Flavobacterium*, *Herbaspirillum*, and *Escherichia‐Shigella*) usually acted as module hubs to stabilize the modular structure, while the rare taxa usually acted as connectors of different modules. We conclude that abundant and rare taxa play different roles in the gut bacterial ecosystem. The conservation of some key bacterial species is essential for promoting the development of the gut microbiome in pandas. The living environment of the giant pandas can influence the internal structure, topological features, and strength of interrelationships in the gut microbiome. This study provides new insights into the conservation and management of giant panda populations.

## 1 INTRODUCTION

The gut microbiome plays an indispensable role in host development (Carlson et al., 2018; Dinan & Cryan, 2016), digestion (LeBlanc et al., 2013; Mackie, 2002), and health (Honda & Littman, 2016; Kau et al., 2011). Conservation metagenomics and research on the gut microbiome can provide important insights for the protection of endangered species (Wei et al., 2018). The giant panda (*Ailuropoda melanoleuca*) is a flagship species for global biodiversity conservation (Wei et al., 2020). The wild populations of giant pandas are mainly distributed in six mountainous areas, namely Qinling (GPQIN), Minshan (GPMS), Qionglai (GPQIO), Daxiangling (GPDXL), Xiaoxiangling (GPXXL), and Liangshan (GPLS), and the captive giant panda individuals (GPCAP) are maintained in several Chinese research facilities for giant panda conservation located in Chengdu, Ya'an, Wolong, and Dujiangyan (Hu et al., 2021; Schaller, 1985). Giant pandas have developed the herbivorous trait of bamboo consumption during long‐term adaptive evolution (Schaller, 1985), and high proportions of Pseudomonadaceae and Clostridiaceae in their gut microbiota play crucial roles in the detoxification of cyanide compounds (Zhu et al., 2018) and digestion of cellulose (Zhu et al., 2011), respectively. Seasonal shifts could affect the structure and function of the giant panda gut microbiome, and recent evidence suggests that *Clostridium butyricum*, which is abundant during the bamboo shoot‐eating season, might enhance the growth of giant pandas by increasing phospholipid biosynthesis (Huang et al., 2022). *Streptococcus* in the gut of giant pandas could be used as a probiotic to cure weight disorders caused by anorexia by controlling the growth of *Clostridium* (Zhao et al., 2021).

Different habitat environments can also affect the composition and diversity of the gut microbiome as well as its survivability (Ning et al., 2020). Data from numerous comparative studies, including those on crocodile lizards (*Shinisaurus crocodilurus*) (Tang, Liang, et al., 2020), alpine musk deer (*Moschus chrysogaster*) (Sun et al., 2020), Przewalski's horses (*Equus ferus przewalskii*) (Metcalf et al., 2017; Tang, Li, et al., 2020), gaur (*Bos gaurus*) (Prabhu et al., 2020), Andean bears (*Tremarctos ornatus*) (Borbón‐García et al., 2017), and amur tigers (*Panthera tigris altaica*) (Ning et al., 2020), have suggested that the analysis of the gut microbiome can help in the conservation of wild and captive individuals (Wei et al., 2018). Several studies have reported differences in the diversity, function, and composition of the gut microbiome between captive and wild giant pandas (Guo et al., 2019; Hu et al., 2021; Yao, Xu, et al., 2019). The same bamboo‐based diet drives the convergent evolution of the gut microbiome in giant panda and red panda (Huang et al., 2021), causing Proteobacteria to dominate the gut microbiome of the wild populations of both species, whereas Firmicutes dominate the gut microbiome of their captive populations (Kong et al., 2014; Xue et al., 2015; Zhang et al., 2018; Zhu et al., 2011). Moreover, there are also significant differences in the gut microbiome among wild giant panda populations distributed in different geographical regions, especially between Qinling and non‐Qinling populations, because long‐term geographic isolation and food selection pressure resulted in a higher proportion of *Clostridium* and vancomycin resistance genes in Qinling giant panda populations (Hu et al., 2021). The gut microbiome of giant pandas has been extensively studied in several contexts, including bacteria (Zhu et al., 2011), viruses (Zhao et al., 2017, 2021), antibiotic resistance genes (Hu et al., 2021), fungi, and protists (Zhu et al., 2021). However, most studies still focus on gut bacterial communities. Exploring the interactions between gut microbial species will shed new light on the maintenance mechanisms of the gut microbial ecosystem in giant pandas.

Network analysis on soil, plant, aquatic, and the human microbiome has reported that it could investigate co‐occurrence patterns among microorganisms in complex ecosystems, promote understanding of microbial interactions, and provide a unique and insightful perspective on microbial interactions and ecological roles (Arumugam et al., 2011; Banerjee et al., 2018; Chen & Wen, 2021; Du et al., 2020; Layeghifard et al., 2017; Mamet et al., 2019; Muegge et al., 2011; Ren et al., 2022; Tian et al., 2020; Xue et al., 2018; Ziegler et al., 2018). In recent years, there has been increasing focus on the importance of rare microbial taxa for the network as a whole (Li, Pujari, et al., 2021; Xue et al., 2018; Ziegler et al., 2018). Rare species may be more sensitive to environmental changes and more susceptible to extinction (Gaston, 2008; Jousset et al., 2017). Network analysis can be used to display the effects of environmental disturbances on the interaction mechanisms of rare microbial taxa (Hunt & Ward, 2015). Network analysis has been applied to the study of the roles of fungi and protists in the gut microbiome of the captive giant pandas, and protists have been found to occupy an important position in the modules (Zhu et al., 2021). To date, little is known about the potential interactions among gut bacterial ecosystems in giant pandas and understanding the internal structure of the gut microbiome can be helpful for their conservation and management. Here, we aimed to advance this aspect of research by studying the differences in internal structure and network topological changes in the gut microbiome between the captive and wild giant panda populations. Also, we aimed to explore the roles of the keystone and rare taxa in the gut bacterial ecosystem. We expected that these findings could provide the basic information for giant panda conservation at the symbiotic microbiome level.

## 2 MATERIALS AND METHODS

### 2.1 Data collection

The V4 region of 16S rRNA gene sequencing raw data of the feces of 494 giant pandas came from our group (Yao, Xu, et al., 2019; Yao, Yang, et al., 2019). A total of 139 fecal samples of giant pandas were collected from the wild Minshan Mountains (GPMS) from June 2012, 148 fecal samples of giant pandas were collected from the wild Xiaoxiangling Mountains (GPXXL) from December 2012 to October 2014, and 207 fecal samples of giant pandas were collected from the captive Chengdu Research Base (GPCAP) from October 2012 to November 2013. The protocol for the analysis of 16S rRNA data was the same as that used in our previous studies (Yao, Xu, et al., 2019; Yao, Yang, et al., 2019).

### 2.2 Co‐occurrence network analysis

The OTUs that ranked in the top 1000 in relative abundance in each group were selected, and subsequently, all possible pairwise Spearman's rank correlations (r) between these OTUs were calculated using the “Picante” R package. Only correlations that were robust (|*ρ*| ≥ 0.6) and statistically significant (*p*‐value ≤0.05) were included in the network (Chen & Wen, 2021). Network visualization and modular analysis were performed using Gephi v.0.9.2 (Xue et al., 2018). Each node represented one OTU, and each edge represented a significant correlation between two nodes. We calculated the topological features (degree, betweenness centrality, closeness centrality, and eigenvector centrality values) for each node in the network using the *igraph* package (Csardi & Nepusz, 2006). The nonparametric Mann–Whitney U test was used to determine the statistical significance of differences in node‐level attributes measured for different taxa (Xue et al., 2018). Based on the data of shared edges and unique edges of different networks, here, the relevant equation already used by Mo et al. was applied to determine the level of dissimilarity of different networks (Koleff et al., 2003; Mo et al., 2021; Poisot et al., 2012). We used Newman's method to calculate modularity values between 0 and 1, which can be used to measure how well a network can be separated into modules (Deng et al., 2012; Newman, 2006). The degree value is a local quantification feature that reflects the number of direct connections with a specific node, and betweenness centrality is the fraction of cases in which a node lies on the shortest path between all pairs of other nodes. Betweenness centrality reflects the impact of a single node on the co‐occurrences of other nodes in the network (Agler et al., 2016; Barberán et al., 2012; Berry & Widder, 2014; Ma et al., 2016).

Keystone species commonly have a significant impact on the network system as a whole, and in contrast to their abundance ratios (Power et al., 1996), nodes with high degree values (>50) and low betweenness centrality values (<5000) are recognized as keystone species in a co‐occurrence network (Berry & Widder, 2014). Different nodes play distinct topological roles in a network, which can be defined by within‐module connectivity (Zi) and among‐module connectivity (Pi). Based on Zi and Pi, nodes are classified into four categories: (i) network hubs: nodes with Zi >2.5 and Pi >0.62; (ii) module hubs: nodes with Zi >2.5 and Pi ≤0.62; (iii) connectors: nodes with Zi ≤2.5 and Pi >0.62; and (iv) peripheral nodes: nodes with Zi ≤2.5 and Pi ≤0.62 (Guimerà & Nunes Amaral, 2005).

### 2.3 Definition of abundant and rare taxa

In recent years, the role of rare taxa has received increasing attention in the marine microbiome (Zhang, Yao, et al., 2021), plant microbiome (Chen & Wen, 2021; Cheng et al., 2021), and soil microbiome (Ma et al., 2016; Tian et al., 2020) studies. It has been shown that rare taxa could occupy key network hubs and enhance buffering and resilience to environmental disturbances through positive correlations with other taxa (Xue et al., 2018). Some methods have been proposed to classify abundant and rare taxa in previous studies (Chen & Wen, 2021; Du et al., 2020; Xue et al., 2018). Here, we defined abundant and rare taxa based on their relative abundance, with 0.01% and 0.1% as the relative abundance thresholds for rare taxa (relative abundance <0.01%), abundant taxa (relative abundance >0.1%), and moderate taxa (0.1% ≥ relative abundance ≥0.01%) (Zhang, Yao, et al., 2021; Zhao et al., 2022).

## 3 RESULTS

### 3.1 Co‐occurrence network analysis indicates differences between captive and wild populations of giant pandas

**FIGURE 1:**
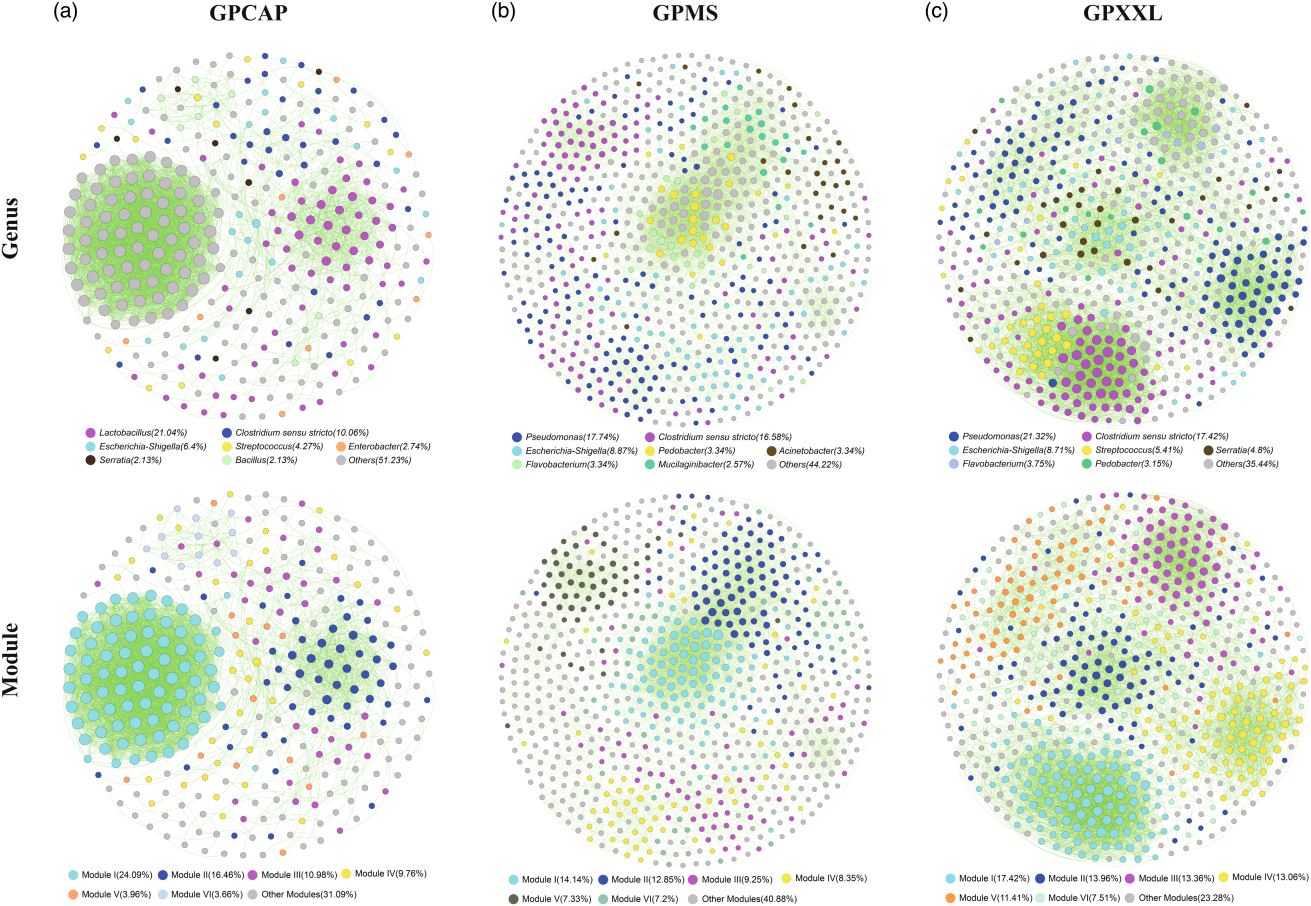
Network co‐occurrence analysis of giant panda gut microbiome among three different populations, GPCAP (a), GPMS (b), and GPXXL (c). The nodes are colored according to different genera and modules, respectively. A connection represented a strong (Spearman's *ρ* ≥ 0.6 or *ρ* ≤ −0.6) and significant (*p*‐value ≤ 0.05) correlation. The size of each node was proportional to the number of connections (degree value). Each major module in GPCAP, GPMS, and GPXXL had more than 11 nodes, 55 nodes, and 49 nodes, respectively. Other modules included all small modules with ≤11, ≤51, and ≤32 nodes per module in GPCAP, GPMS, and GPXXL, respectively. Each circle represented one individual operational taxonomic unit (OTU). For each OTU, abundance was averaged over all samples from each population. Positive and negative correlations were shown as green and red edges, respectively.

In this study, we reanalyzed our V4 region of 16S rRNA sequence data from 494 giant panda fecal samples from three different populations (GPCAP, GPMS, and GPXXL), and beta diversity analysis showed a highly significant difference (*p* < 0.001) in microbial composition among these three groups, indicating the stability of the group clustering patterns (Figure S1 and Table S1). To explore the differences in the gut microbial composition of captive and wild populations of giant pandas, we used 16S rRNA MiSeq amplicon sequencing to analyze the gut bacterial classification of giant panda fecal samples from GPCAP, GPMS, and GPXXL. Comparisons of network co‐occurrence analysis revealed differences in the gut microbiota between wild and captive populations (Figure 1).

In GPCAP, the composition of the gut microbiota was dominated by Firmicutes (47.87%), Proteobacteria (37.5%), Bacteroidetes (4.88%), Planctomycetes (2.13%), Chloroflexi (1.83%), Actinobacteria (1.52%), and Acidobacteria (1.52%) at the phylum level (Figure S2); and *Lactobacillus* (21.04%), *Clostridium* sensu stricto (10.06%), *Escherichia*‐*Shigella* (6.4%), *Streptococcus* (4.27%), *Enterobacter* (2.74%), *Serratia* (2. 13%), and *Bacillus* (2.13%) at the genus level (Figure 1a). In the wild populations, the composition of the gut microbiota was dominated by Proteobacteria (MS:58.23%; XXL:53.15%), Firmicutes (MS:23.26%; XXL:28.23%), Bacteroidetes (MS:14.4%; XXL:12.31%), Actinobacteria (MS:2.06%; XXL:3%), Cyanobacteria (MS:0.77%; XXL:0.75%), Verrucomicrobia (MS:0.77%), Fibrobacteres (MS:0.26%), Acidobacteria (XXL:0.9%), and Planctomycetes (XXL:0.9%) at the phylum level (Figure S2); and *Pseudomonas* (MS:17.74%; XXL:21.32%), *Clostridium* sensu stricto (MS:16.58%; XXL:17.42%), *Escherichia‐Shigella* (MS:8.87%; XXL:8.71%), *Pedobacter* (MS:3.34%; XXL:3.15%), *Flavobacterium* (MS:3.34%; XXL:3.75), *Acinetobacter* (MS:3.34%), *Mucilaginibacter* (MS:2.57%), *Streptococcus* (XXL:5.41%), and *Serratia* (XXL:4.8%) at the genus level (Figure 1b,c). These results suggest that Proteobacteria and Firmicutes were the most abundant phyla in the gut microbiome and that the gut microbiota was dominated by Firmicutes in GPCAP and Proteobacteria in GPMS and GPXXL (Figure S2). Moreover, at the genus level, *Pseudomonas* dominated the wild panda gut microbiome, whereas *Lactobacillus* dominated the captive panda gut microbiome (Figure 1). In addition, the data indicated that the compositions of the gut microbiota in GPCAP and GPXXL were more similar to that in GPMS (Figures 1 and S2).

**FIGURE 2:**
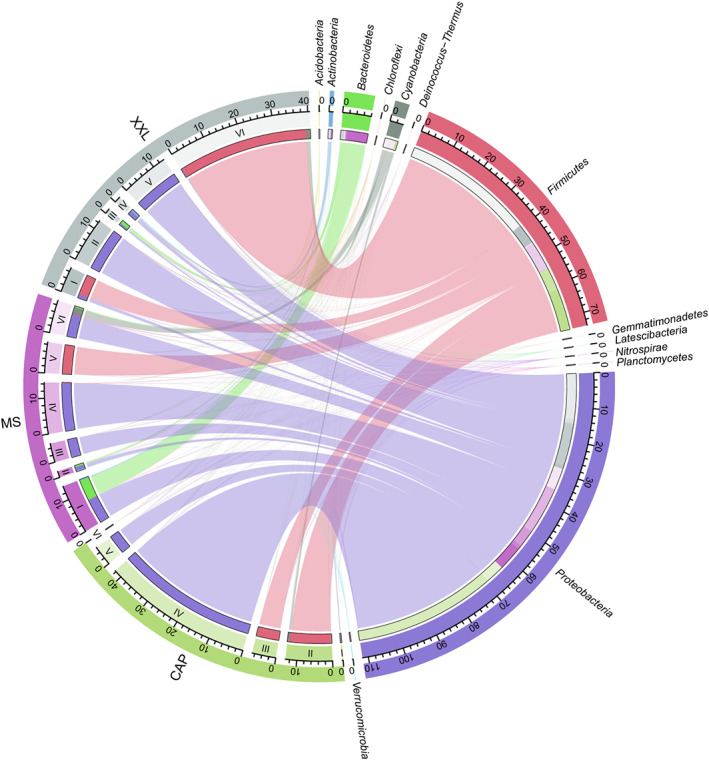
Taxonomic composition of modules in terms of relative abundance of OTUs of phyla in gut microbiome network among three populations (GPCAP, GPMS, and GPXXL). The length of the bars on the outer rings and inner rings represented the percentage and relative abundance of each module type and gut microbiome group (phylum level) in their respective sections, respectively. Each phylum was represented by a specific ribbon color, and the width of each ribbon showed the abundance of each phylum in each module type. I–VI represented the six main modules of the microbial network of three giant panda populations (GPCAP, GPMS, and GPXXL), respectively.

Three metacommunity co‐occurrence networks in GPCAP, GPMS, and GPXXL were built based on correlation relationships and divided into six major modules (Figure 1). The resulting network consisted of 328 nodes linked by 3379 edges in GPCAP, 778 nodes linked by 4860 edges in GPMS, and 666 nodes linked by 5502 edges in GPXXL (Table 1). In addition, the major modules of the gut microbiome in all three populations consisted of Firmicutes and Proteobacteria; however, they had different internal modular structures (Figures 1 and 2 and Table S2), particularly in the case of the two largest modules. Modules I and II in GPCAP were primarily composed of Proteobacteria (60.76%) and Firmicutes (94.64%), respectively, whereas in GPXXL, Modules I and II were primarily composed of Firmicutes (84.97%) and Proteobacteria (97.92%), respectively. Importantly, Bacteroidetes played an indispensable role in the modular structure of GPMS, where both Modules I and II were mainly composed of Proteobacteria (50.00% in Module I and 54.00% in Module II) and Bacteroidetes (44.55% in Module I and 41.00% in Module II). Notably, in all three networks, the number of strong positive correlations was much greater than that of negative correlations (Table 1).

**TABLE 1:** Differences in topological features and modularity analysis of different co‐occurrence networks

Groups	Number of nodes	Number of edges	Number of positive correlations	Number of negative correlations	Probability of positive correlation	Probability of rare taxa	Probability of abundant taxa	Modularity value
GP	607	4844	4818	26	99.46%	59.47%	8.90%	0.608
GPCAP	328	3379	3377	2	99.94%	67.68%	8.84%	0.375
GPMS	778	4860	4846	0	100%	49.61%	12.21%	0.700
GPXXL	666	5502	5499	3	99.95%	72.07%	5.56%	0.733

### 3.2 The overall modular structure of captive and wild giant panda populations

**FIGURE 3:**
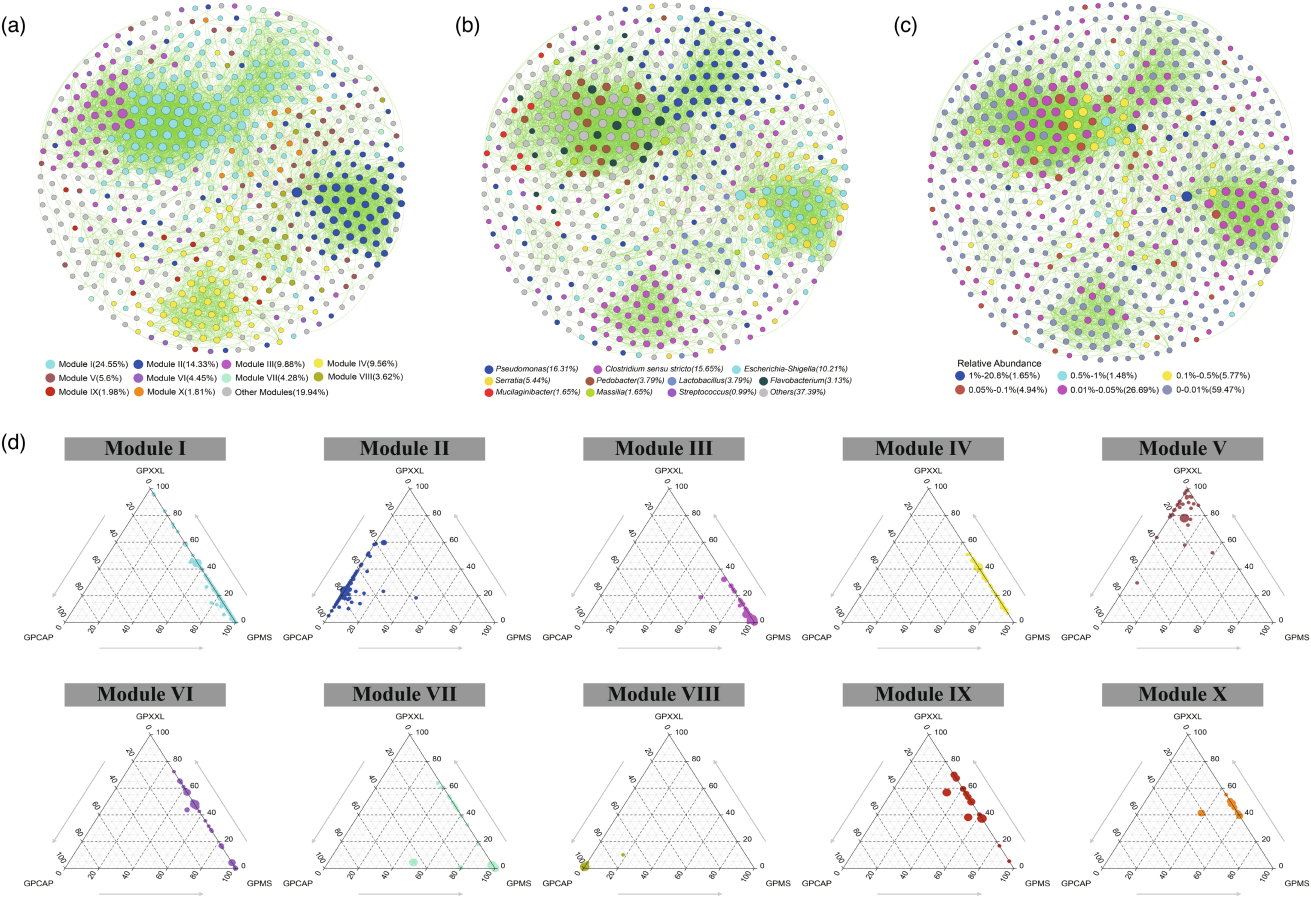
Co‐occurrence patterns of OTUs in all giant panda samples. The nodes are colored according to different modules (a), genera (b), and relative abundance (c), respectively. Ternary plots displayed the relative abundance of OTUs from modules I to X in the three giant panda populations (d). A connection stands for a strong (Spearman's *ρ* ≥ 0.6 or *ρ* ≤ −0.6) and significant (*p*‐value ≤0.05) correlation. The size of each node was proportional to the number of connections (degree value). Each major module had more than 10 nodes. Other modules included all small modules with ≤10 nodes per module. Each circle represents one individual OTU. For each OTU, abundance was averaged over all samples from each population. Positive and negative correlations were shown as green and red edges, respectively.

The entire network based on OTU data from all giant panda populations could be clearly divided into 10 major modules (Figure 3a and Table S3), of which Modules I and II accounted for 24.55% and 14.33%, respectively. The resulting network consisted of 607 nodes linked by 4844 edges, with many more positive correlations than negative correlations (Table 1). Ternary plot analysis indicated that most modules were specific (relatively more abundant) to a particular sampling location (captivity or wild) (Figure 3d). For instance, most of the OTUs from Module I, the largest module, had higher relative abundances in the wild populations (GPMS and GPXXL) than in the captive population (GPCAP) and were dominated by Pseudomonadaceae and Streptomycetaceae; whereas the majority of the OTUs from Module II and VIII had higher relative abundances in the captive population (GPCAP) than in the wild populations (GPMS and GPXXL) and were dominated by Enterobacteriaceae and Lactobacillaceae, respectively.

**FIGURE 4:**
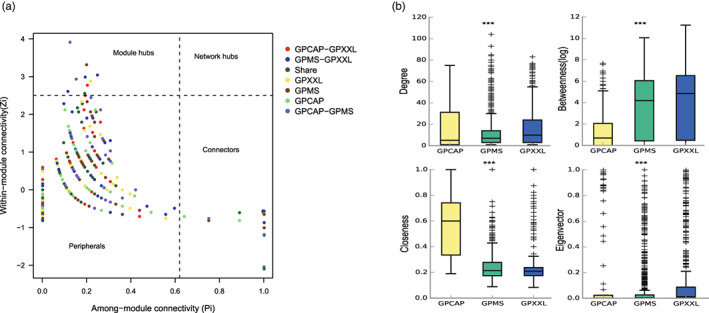
Properties of the co‐occurrence network of OTUs based on correlation. (a) Roles of nodes from the giant panda populations (GP, combing GPCAP, GPMS, and GPXXL) in Zi‐Pi parameter space. Each node in a network could be characterized by its within‐module connectivity (Zi) and its among‐module connectivity (Pi). Nodes with Zi ≥2.5 and Zi <2.5 were classified as module hubs and nonhubs, respectively. Nodes were classified as module hubs (Zi >2.5, Pi <0.62), network hubs (Zi >2.5, Pi >0.62), peripheral nodes (Zi <2.5, Pi <0.62), and connectors (Zi <2.5, Pi >0.62). (b) Comparison of node‐level topological features (degree value, betweenness centrality value, closeness centrality value, and eigenvector centrality value) among three giant panda populations (GPCAP, GPMS, and GPXXL). The top and bottom boundaries of each box indicated the 75th and 25th quartile values, and the lines within each box represented the median values. *** indicated highly significant differences (*p* < 0.001).

According to the connectivity values, within‐module connectivity (*Z*_*i*_) and among‐module connectivity (*P*_*i*_), the roles of nodes were classified into four categories: peripheral nodes, connectors, module hubs, and network hubs (Figure 4a and Table S3). A total of 506 OTUs (83.36%) were peripheral nodes, with most of their links inside their own modules, 92 nodes (15.16%) were connectors, and only nine nodes (1.48%) were module hubs. Among the nine module hubs, four OTUs, mainly belonging to *Flavobacterium*, *Herbaspirillum*, and *Escherichia‐Shigella*, were abundant, and only one OTU (*Bacteriovorax*) was rare. Similarly, out of the 92 OTUs classified as connectors, 9 OTUs were abundant, 23 OTUs were moderately prevalent, and 60 OTUs were rare.

### 3.3 Topology features and modularity

Microbial network dissimilarity analysis showed that the dissimilarity index between the captive and wild populations (about 99%) was greater than that between the two wild giant panda populations (about 87%) (Table S4). We further calculated a set of network‐level topological features among these three populations and found that the values of the degree, betweenness centrality, closeness centrality, and eigenvector centrality in gut bacteria were highly significant (*p* < 0.001) in all three of them. The values of degree, betweenness centrality, and eigenvector centrality in wild individuals (GPMS and GPXXL) were higher than those in captive individuals (GPCAP), but closeness centrality values were higher in captive individuals than those in wild individuals (Figure 4b). Furthermore, modularity analysis showed that the gut microbiomes of wild individuals might have a more similar modular structure, and the modularity value of the captive individuals (GPCAP: 0.375) was lower than that of wild individuals (GPMS: 0.700, GPXXL: 0.733) (Table 1).

### 3.4 Keystone species and rare taxa

Here, we set a standard (degree value >50 and betweenness centrality value <5000) for defining keystone species. A total of 19, 0, 14, and 32 OTUs were defined as keystone species in GP, GPCAP, GPMS, and GPXXL, respectively (Table S5). In GPMS, no keystone species belonged to rare taxa, and they included Sphingomonadaceae (5 OTUs), Oxalobacteraceae (2 OTUs), Comamonadaceae (2 OTUs), no_rank (1 OTU), uncultured (1 OTU), Opitutaceae (1 OTU), Methylophilaceae (1 OTU), and Bacteriovoracaceae (1 OTU). In GPXXL, the keystone species include Clostridiaceae (24 OTUs), Streptococcaceae (4 OTUs), Pseudomonadaceae (1 OTU), Phyllobacteriaceae (1 OTU), Flavobacteriaceae (1 OTU), Aeromonadaceae (1 OTU), and 21 other OTUs (e.g., OTUs from Clostridiaceae, Streptococcaceae, Aeromonadaceae, Flavobacteriaceae, and Pseudomonadaceae) belonged to rare taxa.

OTUs belonging to rare taxa were widely distributed in all populations, but their proportions differed between populations: 67.68% in GPCAP, 49.61% in GPMS, and 72.07% in GPXXL (Table 1). In all populations, the top three most abundant OTUs of rare taxa belonged to Proteobacteria, Firmicutes, and Bacteroidetes (Table S6). The difference was that the rare taxa in captive individuals (GPCAP) were dominated by Firmicutes, while those in the wild individuals (GPMS and GPXXL) were dominated by Proteobacteria. In addition, 65.63% (21 OTUs) of keystone species belonged to rare taxa in GPXXL, and no keystone species in GPMS belonged to rare taxa, which were mainly composed of 28.57% (4 OTUs) abundant taxa and 71.43% (10 OTUs) moderate taxa (Table S5).

## 4 DISCUSSION

### 4.1 High complexity in the gut microbiome of the wild giant panda populations

Microbial interactions play an important role in an ecosystem (Deng et al., 2012; Montoya et al., 2006; Zhou et al., 2010). Here, we used co‐occurrence network analysis to explore the internal modular structures and complex interrelationships in the gut microbiome of giant pandas. This study provides new insights into the structures and interactions of the gut bacterial communities of pandas, which cannot be revealed by the standard α/β diversity metrics that are widely used in microbial ecology (Wu et al., 2021). Previous studies on soil and plant microbiomes have shown that complex microbial networks are more beneficial to the host than simple ones (Ge et al., 2021; Tao et al., 2018). In this study, the degree value, betweenness centrality value, and eigenvector centrality value of the co‐occurrence network in wild individuals (GPMS and GPXXL) were higher than those in captive individuals (GPCAP), except for the closeness centrality value. A higher degree value of a node indicates more interactions and relationships with other nodes, and a higher betweenness centrality value of a node indicates that they are close to the core of the network compared with other nodes (Greenblum et al., 2012; Ma et al., 2016). Thus, we speculated that the gut microbiomes of wild individuals were more closely linked to each other. Furthermore, a high number of nodes and edges reflects high complexity (Chen & Wen, 2021). Highly connected and complex microbial networks may contribute to community stability and better environmental resource utilization and provide higher functional redundancy for the host, enabling it to adapt better to the environment (Morriën et al., 2017; Zhao et al., 2022). In this study, the co‐occurrence network of wild giant pandas also contained more nodes and edges, which indicates higher complexity and stability compared to that of the captive giant pandas. The high topological features of the gut microbiome of giant pandas (especially wild individuals) might reflect their potential adaptability to the intestinal habitat. Strong positive correlations might maintain the stability of the gut microbiota and enhance its buffering capacity against infections (Chen & Wen, 2021). The positive correlations were far stronger than the negative correlations in the gut microbial network of giant pandas, indicating that symbiotic strategies and positive effects (mutualism and synergism) played a more important role than negative effects (competition, parasitism, predation, and antagonism) in their gut microbiota.

Different microorganisms can form complex networks by interacting with each other (Faust & Raes, 2012; Xue et al., 2018), thereby affecting their distribution and function in the ecosystem (Chen & Wen, 2021; Du et al., 2020). Dividing a co‐occurrence network into modules helps gain insights into the different groups of OTUs that perform different functions (Du et al., 2020; Xiong et al., 2018). Module I had higher relative abundances in the wild populations and was occupied by *Pseudomonas*, whereas the majority of the OTUs in Module VIII had higher relative abundances in the captive population, and the module was occupied by *Lactobacillus*. This indicates the presence of different functional units in the gut microbiome of wild and captive pandas. The modularity of bacterial communities can reflect biological interactions and phylogenetic clustering of species with strong interrelationships (Chen & Wen, 2021; Zhou et al., 2011). Higher modularity (the degree of the network to be separated into modules) usually indicates higher habitat heterogeneity and ecological niche diversity (Barberán et al., 2012; Deng et al., 2012; Ren et al., 2017, 2022). Compared with artificially controlled captive environments, the wild environments (e.g., temperature and humidity) are usually versatile, which might lead to the higher modularity values of the two wild giant panda populations than that of the captive populations. Moreover, the difference in the gut microbial community between the wild and captive giant pandas may be associated with the other ecological conditions (e.g., food‐provisioned diet and human contact), which result in the high proportion of *Escherichia‐Shigella* throughout the years and the decreased alpha diversity in the captive giant pandas (Xue et al., 2018; Zhu et al., 2011). Thus, this would further lead to low modularity in the gut microbiome of the captive giant pandas.

### 4.2 Abundant taxa and rare taxa had different roles in the gut bacterial ecosystem

Keystone nodes in co‐occurrence networks tend to have high degree values and low betweenness centrality values based on scale‐free features and have a significant impact on the composition of the gut microbial community as a whole (Barabási, 2009; Berry & Widder, 2014). Like in the case of modules, keystone species can also reflect the key function of a microbial system (Xiong et al., 2021). For example, in GPXXL, 75% of the keystone species belong to the Clostridiaceae family, which is known for its cellulose degradation potential (Xue et al., 2015; Zhang et al., 2018; Zhu et al., 2011). The keystone species in GPMS (14 OTUs) belonged to the phyla Proteobacteria (eight OTUs), Bacteroidetes (four OTUs), and Verrucomicrobia (two OTUs). Compared with other populations, keystone species belonging to Verrucomicrobia were only present in GPMS, and Verrucomicrobia also had a higher relative abundance ranking in GPMS. In addition, *Bacteriovorax* is a bacterial genus specific to wild giant panda populations and is also the main genus in GPMS. These findings indicate the potential effect of geographic region on the internal modular structure.

Rare taxa can occupy key network hubs and enhance buffering and resilience to environmental disturbances by positively correlating with other taxa (Xue et al., 2018). We found that there were always rarer taxa than abundant taxa in the OTUs of all the networks (Table 1), but rare taxa accounted for different proportions of keystone species in different geographical populations (Table S5). Similarly, as has been shown previously, the long‐term geographical isolation has resulted in different gut antibiotic resistance gene distribution characteristics of wild giant panda populations in the Qinling and non‐Qinling Mountains (Hu et al., 2021). Here, a high proportion (65.63%) of keystone species belonged to rare tax in GPXXL (Table S5). We suggested some rare taxa were essential for maintaining the gut bacteria's internal structure and specific functions in GPXXL. However, based on our definitions of keystone species (degree value >50 and betweenness centrality value <5000) and rare taxa (relative abundance <0.01%) in this study, none of these 14 GPMS keystone species belonged to rare taxa. These keystones mainly belonged to Proteobacteria (e.g., Comamonadaceae and Oxalobacteraceae). Comamonadaceae widely distribute in the environments (Moon et al., 2018). The living environment is the important factor affecting the gut microbiome of the host (Bitterlich, 1985; Cui et al., 2022; Lavrinienko et al., 2018; Li et al., 2015; Li, Liu, et al., 2021; Zhang, Yang, et al., 2021). Thus, we deduced that the different environmental conditions in MS might lead to this pattern.

Furthermore, in the microbial network system, rare taxa and abundant taxa played distinct roles. It has been reported that rare taxa could act as network hubs and positively correlate with other taxa to strengthen the resistance of microbial networks to environmental disturbances (Xue et al., 2018). And abundant taxa are also extensively interconnected with other taxa and affect microbial community diversity, structure, and resource cycling (Campbell & Kirchman, 2013; Zhang et al., 2013). In addition, connectors and module hubs also have different positions and topological roles in maintaining microbial networks (Guimerà & Nunes Amaral, 2005). Connectors are primarily determined by among‐module connectivity, which can better enhance the connectivity between other modules, while module hubs are primarily determined by within‐module connectivity, which mainly maintains the stability of independent modules (Guimerà & Nunes Amaral, 2005). In this study, only 9.78% of the nodes in the connectors belong to the abundant taxa, while 65.22% of the species in the connectors belonged to rare taxa and were widely distributed in each module; even modules IX and X were composed entirely of rare taxa. Whereas most of the module hubs (88.89%) belonged to abundant taxa (e.g., OTUs from *Flavobacterium*, *Herbaspirillum*, and *Escherichia‐Shigella*). Therefore, we suggested that abundant taxa and rare taxa played different roles in the gut microbial network of giant pandas. The abundant taxa played a role as modular hubs in maintaining the structure of independent modules. By contrast, rare taxa acted more as connectors between different modules to maintain the overall internal structure of the network.

## 5 CONCLUSION

The results of this study showed that there are differences in the topological features and internal modular structures of the gut microbiomes in captive and wild giant pandas. The gut microbiome of wild giant pandas is more complex than that of captive pandas. The conservation of some key bacterial species was essential for promoting the development of the gut microbiome in pandas. This study improved the understanding of the interconnectedness and potential functional roles of giant panda gut bacteria. However, while the network analysis in this study can shed light on the interactions between gut bacteria, it can only demonstrate a statistical correlation among microbial communities and is not a direct proof of microbial interactions (Ma et al., 2016; Tian et al., 2020).

## FUNDING INFORMATION

Financial support was provided by National Natural Science Foundation of China (32270546) and Priority Academic Program Development of Jiangsu Higher Education Institutions (PAPD).

## CONFLICT OF INTEREST

The authors declare that they have no known competing financial interests or personal relationships that could have appeared to influence the work reported in this article.

## Supporting information


Appendix S1

## Data Availability

Not applicable.
