# Supplementary material for: The putative maintaining mechanism of gut bacterial ecosystem in giant pandas and its potential application in conservation
Source: Evol Appl. 2022 Dec 30;16(1):36–47. doi: 10.1111/eva.13494 (PMC9850007; doi:10.1111/eva.13494)
Supplement: Supplementary file 1 — Appendix S1 [file EVA-16-36-s001.docx]

### Supplementary information of the article:

#### Figure. S1


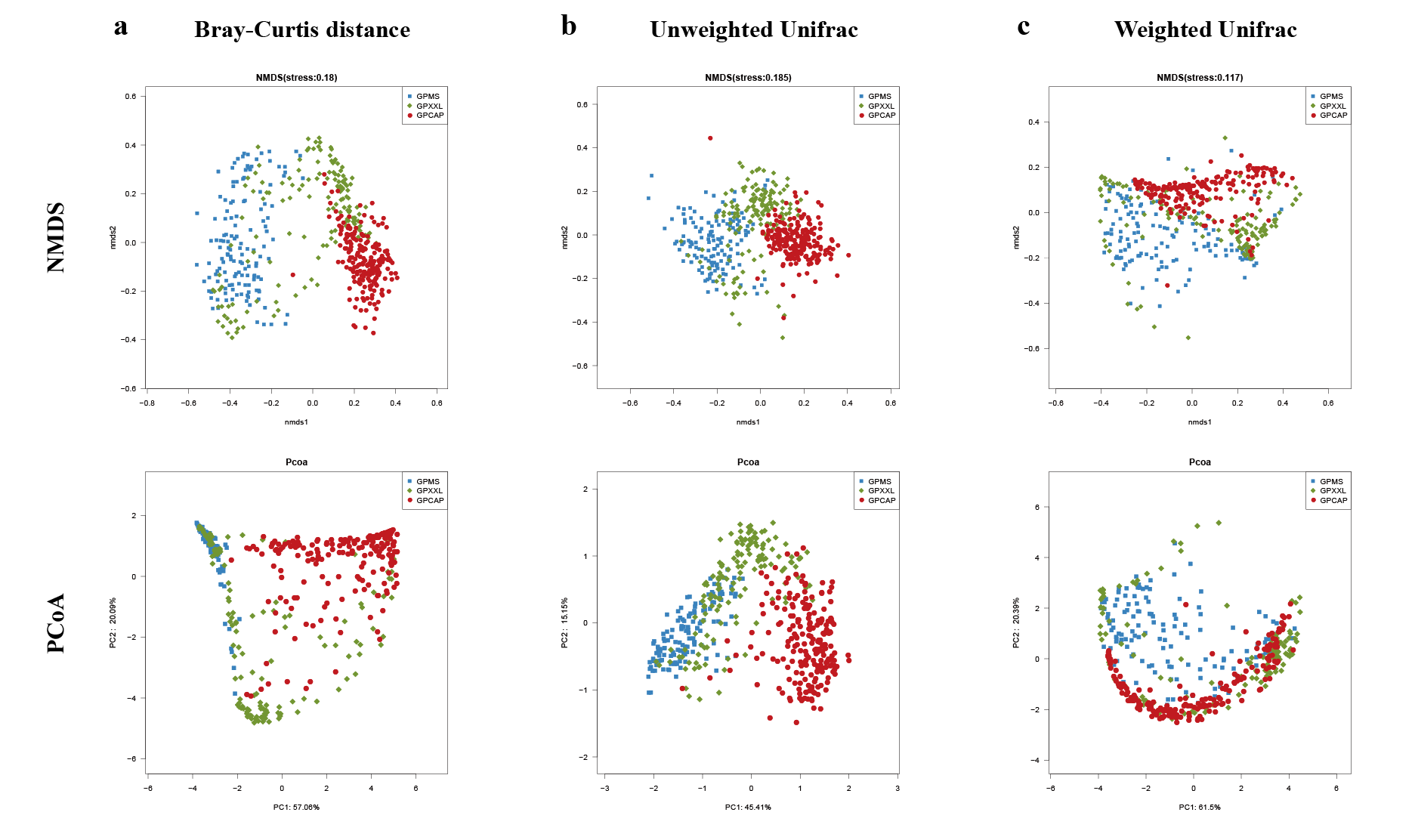


**FIGURE S1** The results of beta diversity among the four groups. The NMDS (non-metric multidimensional scaling) and principle co-ordinates analysis (PCoA) cluster analysis using Bray-Curtis distance**(a)**, unweighted Unifrac distance**(b)** and weighted Unifrac distance**(c)** among all samples, respectively. Each spot represented one OUT. Different shapes and colors denote different groups (GPCAP, GPMS, and GPXXL).

#### Figure. S2


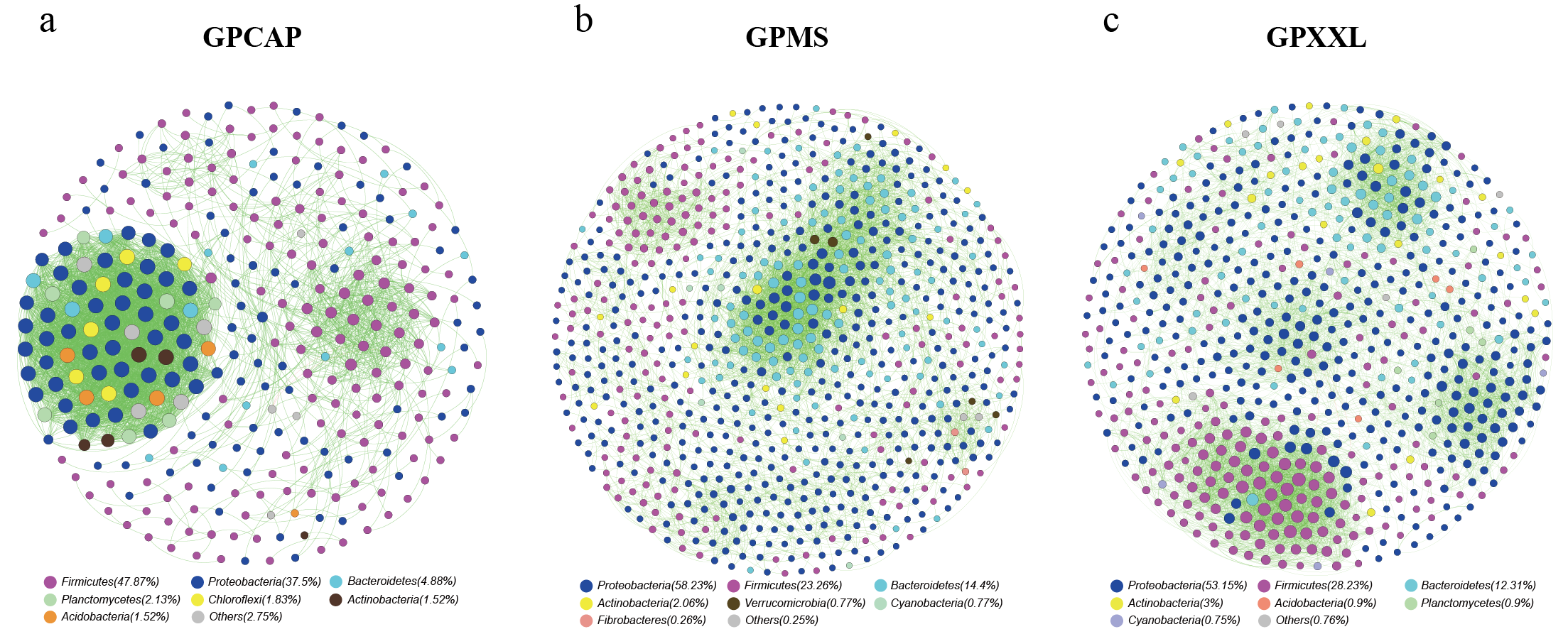


**FIGURE S2** Network co-occurrence analysis of giant panda gut microbiome among three different populations, GPCAP (a), GPMS (b) and GPXXL (c). The nodes are colored according to different phyla. A connection represents a strong (Spearman’s ρ ≥ 0.6 or ρ ≤ -0.6) and significant (P-value ≤ 0.05) correlation. The size of each node is proportional to the number of connections (degree value). Each circle represents one individual operational taxonomic unit (OTU). For each OTU, abundance was averaged over all samples from each population. Positive and negative correlations are shown as green and red edges, respectively.

#### Table. S1

**Table. S1** Analysis of similarity (ADNOIS) statistics of population of giant pandas (GPCAP, GPMS, and GPXXL) testing differences of gut microbiota community groupings at geospatial scales, which based on Bray Curtis, weighted UniFrac and unweighted UniFrac distances.

| Distance | Adnois | | | |
| --- | --- | --- | --- | --- |
|  | Df | F | R^2^ | P |
| Bray Curtis | 2 | 72.812 | 0.22910 | 0.001 |
| Unweighted Unifrac | 2 | 35.840 | 0.12762 | 0.001 |
| Weighted Unifrac | 2 | 43.099 | 0.14960 | 0.001 |

#### Table. S2

**Table. S2** Composition of the top ten modules of co-occurrence network analysis in GPCAP, GPMS and GPXXL.

| Group | Module | Phlum | Genus | Number of OTUs(genus) |
| --- | --- | --- | --- | --- |
| GPCAP | I | Acidobacteria | norank | 3 |
| GPCAP | I | Acidobacteria | DS-100 | 1 |
| GPCAP | I | Actinobacteria | Propioniciclava | 1 |
| GPCAP | I | Actinobacteria | Rhodococcus | 1 |
| GPCAP | I | Actinobacteria | Beutenbergia | 1 |
| GPCAP | I | Actinobacteria | Propionicicella | 1 |
| GPCAP | I | Bacteroidetes | uncultured | 2 |
| GPCAP | I | Bacteroidetes | norank | 2 |
| GPCAP | I | Chloroflexi | norank | 2 |
| GPCAP | I | Chloroflexi | uncultured | 3 |
| GPCAP | I | Chloroflexi | Roseiflexus | 1 |
| GPCAP | I | Firmicutes | Vagococcus | 1 |
| GPCAP | I | Gemmatimonadetes | uncultured | 3 |
| GPCAP | I | Latescibacteria | norank | 1 |
| GPCAP | I | Nitrospirae | Nitrospira | 1 |
| GPCAP | I | Planctomycetes | norank | 1 |
| GPCAP | I | Planctomycetes | Gemmata | 2 |
| GPCAP | I | Planctomycetes | Planctomyces | 3 |
| GPCAP | I | Planctomycetes | uncultured | 1 |
| GPCAP | I | Proteobacteria | Amaricoccus | 2 |
| GPCAP | I | Proteobacteria | Azohydromonas | 1 |
| GPCAP | I | Proteobacteria | Candidatus Alysiosphaera | 3 |
| GPCAP | I | Proteobacteria | Candidatus Competibacter | 1 |
| GPCAP | I | Proteobacteria | Comamonas | 1 |
| GPCAP | I | Proteobacteria | Dechloromonas | 1 |
| GPCAP | I | Proteobacteria | Filomicrobium | 1 |
| GPCAP | I | Proteobacteria | Gemmobacter | 1 |
| GPCAP | I | Proteobacteria | H16 | 1 |
| GPCAP | I | Proteobacteria | Haliangium | 3 |
| GPCAP | I | Proteobacteria | Leisingera | 1 |
| GPCAP | I | Proteobacteria | Luteimonas | 2 |
| GPCAP | I | Proteobacteria | Lysobacter | 1 |
| GPCAP | I | Proteobacteria | Meganema | 2 |
| GPCAP | I | Proteobacteria | norank | 10 |
| GPCAP | I | Proteobacteria | Paracoccus | 1 |
| GPCAP | I | Proteobacteria | Pseudacidovorax | 1 |
| GPCAP | I | Proteobacteria | Rhodobacter | 2 |
| GPCAP | I | Proteobacteria | Rubellimicrobium | 1 |
| GPCAP | I | Proteobacteria | Sphaerotilus | 1 |
| GPCAP | I | Proteobacteria | Tabrizicola | 1 |
| GPCAP | I | Proteobacteria | Tahibacter | 2 |
| GPCAP | I | Proteobacteria | Thiothrix | 1 |
| GPCAP | I | Proteobacteria | uncultured | 7 |
| GPCAP | II | Cyanobacteria | norank | 3 |
| GPCAP | II | Firmicutes | Lactobacillus | 1 |
| GPCAP | II | Firmicutes | Lactococcus | 1 |
| GPCAP | II | Firmicutes | Weissella | 1 |
| GPCAP | II | Firmicutes | Lactobacillus | 50 |
| GPCAP | II | Proteobacteria | norank | 1 |
| GPCAP | III | Firmicutes | Bacillus | 2 |
| GPCAP | III | Firmicutes | Cellulosilyticum | 3 |
| GPCAP | III | Firmicutes | Clostridium sensu stricto 1 | 21 |
| GPCAP | III | Firmicutes | Clostridium sensu stricto 13 | 1 |
| GPCAP | III | Firmicutes | Epulopiscium | 1 |
| GPCAP | III | Firmicutes | Lachnospiraceae NC2004 group | 1 |
| GPCAP | III | Firmicutes | Lysinibacillus | 1 |
| GPCAP | III | Firmicutes | Paenibacillus | 3 |
| GPCAP | III | Firmicutes | Terrisporobacter | 1 |
| GPCAP | III | Firmicutes | Turicibacter | 2 |
| GPCAP | IV | Proteobacteria | Cedecea | 1 |
| GPCAP | IV | Proteobacteria | Escherichia-Shigella | 18 |
| GPCAP | IV | Proteobacteria | Hafnia-Obesumbacterium | 2 |
| GPCAP | IV | Proteobacteria | norank | 1 |
| GPCAP | IV | Proteobacteria | Plesiomonas | 1 |
| GPCAP | IV | Proteobacteria | Rosenbergiella | 1 |
| GPCAP | IV | Proteobacteria | Serratia | 8 |
| GPCAP | V | Proteobacteria | Enterobacter | 5 |
| GPCAP | V | Proteobacteria | Erwinia | 2 |
| GPCAP | V | Proteobacteria | Klebsiella | 2 |
| GPCAP | V | Proteobacteria | Kluyvera | 1 |
| GPCAP | V | Proteobacteria | Pantoea | 2 |
| GPCAP | V | Proteobacteria | Salmonella | 1 |
| GPCAP | VI | Proteobacteria | Pseudomonas | 1 |
| GPCAP | VI | Firmicutes | Streptococcus | 2 |
| GPCAP | VI | Firmicutes | Psychrobacillus | 1 |
| GPCAP | VI | Firmicutes | Kurthia | 1 |
| GPCAP | VI | Firmicutes | Carnobacterium | 2 |
| GPCAP | VI | Firmicutes | Bacillus | 5 |
| GPCAP | VII | Bacteroidetes | Arachidicoccus | 1 |
| GPCAP | VII | Bacteroidetes | Chryseobacterium | 3 |
| GPCAP | VII | Bacteroidetes | Moheibacter | 1 |
| GPCAP | VII | Proteobacteria | Lysobacter | 2 |
| GPCAP | VII | Proteobacteria | Ottowia | 1 |
| GPCAP | VII | Proteobacteria | Rhizorhapis | 1 |
| GPCAP | VII | Proteobacteria | Sphingomonas | 1 |
| GPCAP | VII | Proteobacteria | uncultured | 1 |
| GPCAP | VII | Firmicutes | Lactobacillus | 7 |
| GPCAP | IX | Firmicutes | Clostridium sensu stricto 1 | 1 |
| GPCAP | IX | Firmicutes | Clostridium sensu stricto 13 | 1 |
| GPCAP | IX | Firmicutes | Sarcina | 4 |
| GPCAP | IX | Proteobacteria | Escherichia-Shigella | 1 |
| GPCAP | X | Firmicutes | Clostridium sensu stricto 1 | 4 |
| GPCAP | X | Firmicutes | Enterococcus | 1 |
| GPMS | I | Actinobacteria | Herbiconiux | 1 |
| GPMS | I | Actinobacteria | Ruania | 1 |
| GPMS | I | Actinobacteria | Microbacterium | 1 |
| GPMS | I | Actinobacteria | Sanguibacter | 1 |
| GPMS | I | Actinobacteria | Galbitalea | 1 |
| GPMS | I | Actinobacteria | Glutamicibacter | 1 |
| GPMS | I | Bacteroidetes | Chryseobacterium | 5 |
| GPMS | I | Bacteroidetes | Dyadobacter | 1 |
| GPMS | I | Bacteroidetes | Epilithonimonas | 1 |
| GPMS | I | Bacteroidetes | Flavobacterium | 18 |
| GPMS | I | Bacteroidetes | Fluviicola | 1 |
| GPMS | I | Bacteroidetes | Mucilaginibacter | 1 |
| GPMS | I | Bacteroidetes | Pedobacter | 20 |
| GPMS | I | Bacteroidetes | Sphingobacterium | 1 |
| GPMS | I | Bacteroidetes | uncultured | 1 |
| GPMS | I | Proteobacteria | Acidovorax | 1 |
| GPMS | I | Proteobacteria | Advenella | 1 |
| GPMS | I | Proteobacteria | Bacteriovorax | 2 |
| GPMS | I | Proteobacteria | Brevundimonas | 3 |
| GPMS | I | Proteobacteria | Caenimonas | 1 |
| GPMS | I | Proteobacteria | Cellvibrio | 2 |
| GPMS | I | Proteobacteria | Devosia | 6 |
| GPMS | I | Proteobacteria | Duganella | 4 |
| GPMS | I | Proteobacteria | Herbaspirillum | 4 |
| GPMS | I | Proteobacteria | Hydrogenophaga | 1 |
| GPMS | I | Proteobacteria | Janthinobacterium | 1 |
| GPMS | I | Proteobacteria | Kaistia | 1 |
| GPMS | I | Proteobacteria | Limnohabitans | 1 |
| GPMS | I | Proteobacteria | Massilia | 6 |
| GPMS | I | Proteobacteria | Methylophilus | 2 |
| GPMS | I | Proteobacteria | Novosphingobium | 1 |
| GPMS | I | Proteobacteria | Paucimonas | 1 |
| GPMS | I | Proteobacteria | Polaromonas | 1 |
| GPMS | I | Proteobacteria | Pseudochrobactrum | 1 |
| GPMS | I | Proteobacteria | Pseudomonas | 3 |
| GPMS | I | Proteobacteria | Pseudorhodoferax | 1 |
| GPMS | I | Proteobacteria | Rhizobium | 3 |
| GPMS | I | Proteobacteria | Sphingomonas | 1 |
| GPMS | I | Proteobacteria | Stenotrophomonas | 1 |
| GPMS | I | Proteobacteria | Variovorax | 3 |
| GPMS | I | Proteobacteria | Xanthomonas | 2 |
| GPMS | I | Proteobacteria | Xylophilus | 1 |
| GPMS | II | Bacteroidetes | Pedobacter | 5 |
| GPMS | II | Bacteroidetes | norank | 4 |
| GPMS | II | Bacteroidetes | Mucilaginibacter | 19 |
| GPMS | II | Bacteroidetes | Fluviicola | 3 |
| GPMS | II | Bacteroidetes | Flavobacterium | 5 |
| GPMS | II | Bacteroidetes | Cytophaga | 4 |
| GPMS | II | Bacteroidetes | Chitinophaga | 1 |
| GPMS | II | Firmicutes | Pelosinus | 2 |
| GPMS | II | Proteobacteria | Asticcacaulis | 2 |
| GPMS | II | Proteobacteria | Aureimonas | 1 |
| GPMS | II | Proteobacteria | Bacteriovorax | 1 |
| GPMS | II | Proteobacteria | Brevundimonas | 2 |
| GPMS | II | Proteobacteria | Candidatus Odyssella | 1 |
| GPMS | II | Proteobacteria | Caulobacter | 3 |
| GPMS | II | Proteobacteria | Cellvibrio | 1 |
| GPMS | II | Proteobacteria | Devosia | 1 |
| GPMS | II | Proteobacteria | Duganella | 1 |
| GPMS | II | Proteobacteria | Herminiimonas | 1 |
| GPMS | II | Proteobacteria | Janthinobacterium | 1 |
| GPMS | II | Proteobacteria | Leptothrix | 1 |
| GPMS | II | Proteobacteria | Massilia | 4 |
| GPMS | II | Proteobacteria | Methylobacterium | 1 |
| GPMS | II | Proteobacteria | norank | 7 |
| GPMS | II | Proteobacteria | Novosphingobium | 1 |
| GPMS | II | Proteobacteria | Polaromonas | 1 |
| GPMS | II | Proteobacteria | Pseudomonas | 3 |
| GPMS | II | Proteobacteria | Rhizobacter | 1 |
| GPMS | II | Proteobacteria | Rhizobium | 2 |
| GPMS | II | Proteobacteria | Rhodopseudomonas | 1 |
| GPMS | II | Proteobacteria | Rubrivivax | 1 |
| GPMS | II | Proteobacteria | Rugamonas | 1 |
| GPMS | II | Proteobacteria | Sphaerotilus | 1 |
| GPMS | II | Proteobacteria | Sphingobium | 2 |
| GPMS | II | Proteobacteria | Sphingomonas | 5 |
| GPMS | II | Proteobacteria | Tardiphaga | 2 |
| GPMS | II | Proteobacteria | uncultured | 4 |
| GPMS | II | Proteobacteria | Verticia | 1 |
| GPMS | II | Verrucomicrobia | norank | 2 |
| GPMS | II | Verrucomicrobia | Opitutus | 1 |
| GPMS | III | Proteobacteria | Citrobacter | 1 |
| GPMS | III | Proteobacteria | Cronobacter | 1 |
| GPMS | III | Proteobacteria | Enterobacter | 1 |
| GPMS | III | Proteobacteria | Escherichia-Shigella | 69 |
| GPMS | IV | Firmicutes | Lysinibacillus | 1 |
| GPMS | IV | Firmicutes | Bacillus | 1 |
| GPMS | IV | Firmicutes | Lachnoclostridium | 1 |
| GPMS | IV | Proteobacteria | Acinetobacter | 3 |
| GPMS | IV | Proteobacteria | Pseudomonas | 59 |
| GPMS | V | Firmicutes | [Ruminococcus] torques group | 1 |
| GPMS | V | Firmicutes | Clostridium sensu stricto 1 | 49 |
| GPMS | V | Firmicutes | Lachnospiraceae NC2004 group | 1 |
| GPMS | V | Firmicutes | Terrisporobacter | 1 |
| GPMS | V | Firmicutes | Turicibacter | 3 |
| GPMS | V | Proteobacteria | Halomonas | 2 |
| GPMS | VI | Bacteroidetes | Chryseobacterium | 1 |
| GPMS | VI | Bacteroidetes | Empedobacter | 1 |
| GPMS | VI | Bacteroidetes | Myroides | 2 |
| GPMS | VI | Bacteroidetes | norank | 1 |
| GPMS | VI | Bacteroidetes | Sphingobacterium | 2 |
| GPMS | VI | Cyanobacteria | norank | 1 |
| GPMS | VI | Firmicutes | Paenibacillus | 1 |
| GPMS | VI | Proteobacteria | Acidovorax | 1 |
| GPMS | VI | Proteobacteria | Acinetobacter | 20 |
| GPMS | VI | Proteobacteria | Comamonas | 3 |
| GPMS | VI | Proteobacteria | Diaphorobacter | 2 |
| GPMS | VI | Proteobacteria | Enterobacter | 1 |
| GPMS | VI | Proteobacteria | Hafnia-Obesumbacterium | 1 |
| GPMS | VI | Proteobacteria | Kluyvera | 1 |
| GPMS | VI | Proteobacteria | Lampropedia | 1 |
| GPMS | VI | Proteobacteria | Providencia | 1 |
| GPMS | VI | Proteobacteria | Pseudomonas | 1 |
| GPMS | VI | Proteobacteria | Raoultella | 4 |
| GPMS | VI | Proteobacteria | Rosenbergiella | 1 |
| GPMS | VI | Proteobacteria | Serratia | 4 |
| GPMS | VI | Proteobacteria | Sphaerotilus | 1 |
| GPMS | VI | Proteobacteria | Variovorax | 1 |
| GPMS | VI | Proteobacteria | Wohlfahrtiimonas | 1 |
| GPMS | VI | Proteobacteria | Yersinia | 3 |
| GPMS | VII | Actinobacteria | E1B-B3-114 | 1 |
| GPMS | VII | Proteobacteria | Pseudomonas | 49 |
| GPMS | VII | Proteobacteria | Psychrobacter | 1 |
| GPMS | VIII | Cyanobacteria | norank | 2 |
| GPMS | VIII | Firmicutes | Clostridium sensu stricto 1 | 25 |
| GPMS | VIII | Proteobacteria | Serratia | 2 |
| GPMS | VIII | Proteobacteria | Pseudomonas | 1 |
| GPMS | VIII | Proteobacteria | Proteus | 1 |
| GPMS | VIII | Proteobacteria | Klebsiella | 1 |
| GPMS | VIII | Proteobacteria | Comamonas | 2 |
| GPMS | VIII | Proteobacteria | Acinetobacter | 1 |
| GPMS | IX | Acidobacteria | Geothrix | 1 |
| GPMS | IX | Bacteroidetes | Cytophaga | 1 |
| GPMS | IX | Bacteroidetes | norank | 2 |
| GPMS | IX | Bacteroidetes | Paludibacter | 2 |
| GPMS | IX | Bacteroidetes | uncultured | 3 |
| GPMS | IX | Fibrobacteres | uncultured | 1 |
| GPMS | IX | Fibrobacteres | possible genus 04 | 1 |
| GPMS | IX | Planctomycetes | norank | 1 |
| GPMS | IX | Proteobacteria | Candidatus Accumulibacter | 1 |
| GPMS | IX | Proteobacteria | Denitratisoma | 1 |
| GPMS | IX | Proteobacteria | Halomonas | 1 |
| GPMS | IX | Proteobacteria | Paucimonas | 1 |
| GPMS | IX | Proteobacteria | Pseudohongiella | 2 |
| GPMS | IX | Proteobacteria | Pseudoxanthomonas | 1 |
| GPMS | IX | Proteobacteria | Reyranella | 1 |
| GPMS | IX | Proteobacteria | Rhizomicrobium | 4 |
| GPMS | IX | Proteobacteria | Sphingomonas | 1 |
| GPMS | IX | Proteobacteria | uncultured | 7 |
| GPMS | IX | Verrucomicrobia | Opitutus | 3 |
| GPMS | X | Actinobacteria | Micrococcus | 1 |
| GPMS | X | Actinobacteria | Nesterenkonia | 1 |
| GPMS | X | Cyanobacteria | norank | 1 |
| GPMS | X | Proteobacteria | Acinetobacter | 1 |
| GPMS | X | Proteobacteria | Aliihoeflea | 2 |
| GPMS | X | Proteobacteria | Enterobacter | 1 |
| GPMS | X | Proteobacteria | Halomonas | 17 |
| GPMS | X | Proteobacteria | Mannheimia | 1 |
| GPMS | X | Proteobacteria | Pantoea | 1 |
| GPMS | X | Proteobacteria | Pelagibacterium | 3 |
| GPMS | X | Proteobacteria | Pseudomonas | 1 |
| GPMS | X | Proteobacteria | Serratia | 1 |
| GPMS | X | Proteobacteria | uncultured | 2 |
| GPXXL | I | Actinobacteria | Nesterenkonia | 1 |
| GPXXL | I | Bacteroidetes | Elizabethkingia | 1 |
| GPXXL | I | Cyanobacteria | norank | 1 |
| GPXXL | I | Deinococcus-Thermus | Thermus | 1 |
| GPXXL | I | Firmicutes | Clostridium sensu stricto 1 | 113 |
| GPXXL | I | Firmicutes | Streptococcus | 31 |
| GPXXL | I | Firmicutes | Terrisporobacter | 2 |
| GPXXL | I | Firmicutes | Turicibacter | 1 |
| GPXXL | I | Proteobacteria | Acinetobacter | 1 |
| GPXXL | I | Proteobacteria | Aeromonas | 1 |
| GPXXL | I | Proteobacteria | Aliihoeflea | 2 |
| GPXXL | I | Proteobacteria | Aquabacterium | 1 |
| GPXXL | I | Proteobacteria | Caulobacter | 1 |
| GPXXL | I | Proteobacteria | Citrobacter | 2 |
| GPXXL | I | Proteobacteria | Enterobacter | 1 |
| GPXXL | I | Proteobacteria | Escherichia-Shigella | 1 |
| GPXXL | I | Proteobacteria | Halomonas | 3 |
| GPXXL | I | Proteobacteria | Klebsiella | 2 |
| GPXXL | I | Proteobacteria | Methyloversatilis | 1 |
| GPXXL | I | Proteobacteria | norank | 1 |
| GPXXL | I | Proteobacteria | Pantoea | 1 |
| GPXXL | I | Proteobacteria | Pelagibacterium | 1 |
| GPXXL | I | Proteobacteria | Pseudomonas | 1 |
| GPXXL | I | Proteobacteria | Sphingobium | 1 |
| GPXXL | I | Proteobacteria | uncultured | 1 |
| GPXXL | II | Firmicutes | Streptococcus | 1 |
| GPXXL | II | Firmicutes | Veillonella | 1 |
| GPXXL | II | Proteobacteria | Cedecea | 1 |
| GPXXL | II | Proteobacteria | Cronobacter | 3 |
| GPXXL | II | Proteobacteria | Escherichia-Shigella | 53 |
| GPXXL | II | Proteobacteria | Hafnia-Obesumbacterium | 2 |
| GPXXL | II | Proteobacteria | norank | 1 |
| GPXXL | II | Proteobacteria | Plesiomonas | 1 |
| GPXXL | II | Proteobacteria | Rahnella | 1 |
| GPXXL | II | Proteobacteria | Salmonella | 1 |
| GPXXL | II | Proteobacteria | Serratia | 28 |
| GPXXL | II | Proteobacteria | Yersinia | 3 |
| GPXXL | III | Actinobacteria | Arthrobacter | 1 |
| GPXXL | III | Actinobacteria | Leifsonia | 1 |
| GPXXL | III | Actinobacteria | Microbacterium | 1 |
| GPXXL | III | Actinobacteria | Micrococcus | 1 |
| GPXXL | III | Actinobacteria | Mycetocola | 1 |
| GPXXL | III | Actinobacteria | Paeniglutamicibacter | 2 |
| GPXXL | III | Actinobacteria | Rhodococcus | 1 |
| GPXXL | III | Bacteroidetes | Chryseobacterium | 3 |
| GPXXL | III | Bacteroidetes | Dyadobacter | 1 |
| GPXXL | III | Bacteroidetes | Dysgonomonas | 1 |
| GPXXL | III | Bacteroidetes | Empedobacter | 1 |
| GPXXL | III | Bacteroidetes | Epilithonimonas | 1 |
| GPXXL | III | Bacteroidetes | Flavobacterium | 9 |
| GPXXL | III | Bacteroidetes | Myroides | 2 |
| GPXXL | III | Bacteroidetes | norank | 1 |
| GPXXL | III | Bacteroidetes | Pedobacter | 11 |
| GPXXL | III | Bacteroidetes | Sphingobacterium | 4 |
| GPXXL | III | Bacteroidetes | Taibaiella | 2 |
| GPXXL | III | Proteobacteria | Acidovorax | 3 |
| GPXXL | III | Proteobacteria | Acinetobacter | 1 |
| GPXXL | III | Proteobacteria | Bacteriovorax | 1 |
| GPXXL | III | Proteobacteria | Brevundimonas | 3 |
| GPXXL | III | Proteobacteria | Cellvibrio | 1 |
| GPXXL | III | Proteobacteria | Comamonas | 4 |
| GPXXL | III | Proteobacteria | Diaphorobacter | 2 |
| GPXXL | III | Proteobacteria | Duganella | 3 |
| GPXXL | III | Proteobacteria | Herbaspirillum | 1 |
| GPXXL | III | Proteobacteria | Lampropedia | 1 |
| GPXXL | III | Proteobacteria | Limnohabitans | 1 |
| GPXXL | III | Proteobacteria | Massilia | 3 |
| GPXXL | III | Proteobacteria | Methylophilus | 2 |
| GPXXL | III | Proteobacteria | Paucimonas | 1 |
| GPXXL | III | Proteobacteria | Polaromonas | 1 |
| GPXXL | III | Proteobacteria | Pseudochrobactrum | 1 |
| GPXXL | III | Proteobacteria | Pseudomonas | 3 |
| GPXXL | III | Proteobacteria | Pseudorhodoferax | 1 |
| GPXXL | III | Proteobacteria | Pseudoxanthomonas | 1 |
| GPXXL | III | Proteobacteria | Rhizobium | 2 |
| GPXXL | III | Proteobacteria | Rubellimicrobium | 1 |
| GPXXL | III | Proteobacteria | Sphingomonas | 1 |
| GPXXL | III | Proteobacteria | Stenotrophomonas | 3 |
| GPXXL | III | Proteobacteria | Variovorax | 2 |
| GPXXL | III | Proteobacteria | Xanthomonas | 1 |
| GPXXL | III | Proteobacteria | Xylophilus | 1 |
| GPXXL | IV | Actinobacteria | Arthrobacter | 1 |
| GPXXL | IV | Actinobacteria | E1B-B3-114 | 1 |
| GPXXL | IV | Bacteroidetes | Pedobacter | 1 |
| GPXXL | IV | Proteobacteria | Duganella | 1 |
| GPXXL | IV | Proteobacteria | Massilia | 1 |
| GPXXL | IV | Proteobacteria | Pseudomonas | 74 |
| GPXXL | IV | Proteobacteria | Psychrobacter | 1 |
| GPXXL | IV | Proteobacteria | Raoultella | 2 |
| GPXXL | IV | Proteobacteria | Serratia | 4 |
| GPXXL | IV | Proteobacteria | Xanthomonas | 1 |
| GPXXL | V | Proteobacteria | Acidovorax | 1 |
| GPXXL | V | Proteobacteria | Escherichia-Shigella | 1 |
| GPXXL | V | Proteobacteria | Pseudomonas | 60 |
| GPXXL | V | Proteobacteria | Stenotrophomonas | 1 |
| GPXXL | V | Firmicutes | Enterococcus | 1 |
| GPXXL | V | Firmicutes | Psychrobacillus | 1 |
| GPXXL | V | Bacteroidetes | Flavobacterium | 3 |
| GPXXL | V | Bacteroidetes | Pedobacter | 1 |
| GPXXL | V | Actinobacteria | Cryobacterium | 1 |
| GPXXL | V | Actinobacteria | Leifsonia | 1 |
| GPXXL | V | Actinobacteria | Pseudarthrobacter | 3 |
| GPXXL | V | Actinobacteria | Ruania | 1 |
| GPXXL | V | Actinobacteria | Sanguibacter | 1 |
| GPXXL | VI | Acidobacteria | Edaphobacter | 1 |
| GPXXL | VI | Acidobacteria | Granulicella | 2 |
| GPXXL | VI | Acidobacteria | Terriglobus | 1 |
| GPXXL | VI | Cyanobacteria | norank | 1 |
| GPXXL | VI | Firmicutes | Clostridium sensu stricto 1 | 32 |
| GPXXL | VI | Firmicutes | Lachnospiraceae NC2004 group | 1 |
| GPXXL | VI | Firmicutes | Streptococcus | 4 |
| GPXXL | VI | Proteobacteria | norank | 5 |
| GPXXL | VI | Proteobacteria | Rhodopseudomonas | 1 |
| GPXXL | VII | Actinobacteria | Nesterenkonia | 1 |
| GPXXL | VII | Cyanobacteria | norank | 1 |
| GPXXL | VII | Firmicutes | Clostridium sensu stricto 1 | 16 |
| GPXXL | VII | Firmicutes | Leuconostoc | 1 |
| GPXXL | VII | Firmicutes | Lysinibacillus | 1 |
| GPXXL | VII | Firmicutes | Peptoclostridium | 1 |
| GPXXL | VII | Firmicutes | Terrisporobacter | 2 |
| GPXXL | VII | Firmicutes | Turicibacter | 1 |
| GPXXL | VII | Proteobacteria | Acinetobacter | 1 |
| GPXXL | VII | Proteobacteria | Aquabacterium | 2 |
| GPXXL | VII | Proteobacteria | Enterobacter | 1 |
| GPXXL | VII | Proteobacteria | Klebsiella | 1 |
| GPXXL | VII | Proteobacteria | Serratia | 1 |
| GPXXL | VII | Proteobacteria | Stenotrophomonas | 1 |
| GPXXL | VII | Proteobacteria | uncultured | 1 |
| GPXXL | VIII | Bacteroidetes | Chryseobacterium | 1 |
| GPXXL | VIII | Bacteroidetes | Flavobacterium | 11 |
| GPXXL | VIII | Bacteroidetes | Pedobacter | 7 |
| GPXXL | VIII | Bacteroidetes | Sphingobacterium | 2 |
| GPXXL | VIII | Proteobacteria | Duganella | 1 |
| GPXXL | VIII | Proteobacteria | Massilia | 1 |
| GPXXL | VIII | Proteobacteria | Pseudomonas | 2 |
| GPXXL | VIII | Proteobacteria | Sphaerotilus | 1 |
| GPXXL | VIII | Proteobacteria | uncultured | 1 |
| GPXXL | IX | Firmicutes | Clostridium sensu stricto 1 | 6 |
| GPXXL | IX | Firmicutes | Peptoclostridium | 4 |
| GPXXL | IX | Firmicutes | Streptococcus | 1 |
| GPXXL | IX | Firmicutes | Terrisporobacter | 3 |
| GPXXL | IX | Firmicutes | Turicibacter | 5 |
| GPXXL | X | Acidobacteria | Candidatus Koribacter | 1 |
| GPXXL | X | Acidobacteria | norank | 1 |
| GPXXL | X | Planctomycetes | Gemmata | 1 |
| GPXXL | X | Planctomycetes | Pir4 lineage | 1 |
| GPXXL | X | Planctomycetes | Planctomyces | 1 |
| GPXXL | X | Planctomycetes | uncultured | 3 |
| GPXXL | X | Proteobacteria | Bradyrhizobium | 1 |
| GPXXL | X | Proteobacteria | Nitrobacter | 1 |
| GPXXL | X | Proteobacteria | norank | 2 |
| GPXXL | X | Proteobacteria | Pseudolabrys | 1 |
| GPXXL | X | Proteobacteria | Reyranella | 1 |
| GPXXL | X | Proteobacteria | Tabrizicola | 1 |

#### Table. S3

**Table. S3** Information on module composition and topography role in GP.

| **OTU-ID** | **Zi** | **Pi** | **Module** | **Degree** | **relative-abundance** | **Topography roles** | **Abundance classification** | **Taxonomy** |
| --- | --- | --- | --- | --- | --- | --- | --- | --- |
| DQ816918.1.1321 | -0.60126 | 0 | Other | 5 | 0.0026% | peripheral-nodes | Rare | d__Bacteria;p__Firmicutes;c__Clostridia;o__Clostridiales;f__Clostridiaceae 1;g__Clostridium sensu stricto 1;s__uncultured bacterium |
| KJ728853.1.1393 | -1.21218 | 1 | III | 19 | 0.0026% | connectors | Rare | d__Bacteria;p__Bacteroidetes;c__Sphingobacteriia;o__Sphingobacteriales;f__Sphingobacteriaceae;g__Mucilaginibacter;s__Mucilaginibacter sp. HME9299 |
| FJ893936.1.1358 | -0.22099 | 0 | I | 12 | 0.0027% | peripheral-nodes | Rare | d__Bacteria;p__Proteobacteria;c__Gammaproteobacteria;o__Pseudomonadales;f__Pseudomonadaceae;g__Pseudomonas;s__uncultured bacterium |
| FJ849614.1.1465 | -0.76423 | 0.555556 | Other | 3 | 0.0027% | peripheral-nodes | Rare | d__Bacteria;p__Proteobacteria;c__Gammaproteobacteria;o__Enterobacteriales;f__Enterobacteriaceae;g__Serratia;s__uncultured bacterium |
| JQ426027.1.1453 | -0.76423 | 0.555556 | Other | 3 | 0.0027% | peripheral-nodes | Rare | d__Bacteria;p__Proteobacteria;c__Deltaproteobacteria;o__Myxococcales;f__Sandaracinaceae;g__uncultured;s__uncultured bacterium |
| HQ190454.1.1493 | -0.65555 | 1 | Other | 7 | 0.0027% | connectors | Rare | d__Bacteria;p__Bacteroidetes;c__Cytophagia;o__Cytophagales;f__Cytophagaceae;g__uncultured;s__uncultured bacterium |
| KP120814.1.1405 | -0.70991 | 0.4375 | IX | 4 | 0.0027% | peripheral-nodes | Rare | d__Bacteria;p__Proteobacteria;c__Gammaproteobacteria;o__Enterobacteriales;f__Enterobacteriaceae;g__Citrobacter;s__bacterium 118_39 |
| KF756313.1.1238 | -0.27532 | 0 | VII | 11 | 0.0027% | peripheral-nodes | Rare | d__Bacteria;p__Proteobacteria;c__Gammaproteobacteria;o__Pseudomonadales;f__Pseudomonadaceae;g__Pseudomonas;s__uncultured Pseudomonas sp. |
| FN667084.1.1493 | -0.22099 | 0 | VIII | 12 | 0.0027% | peripheral-nodes | Rare | d__Bacteria;p__Firmicutes;c__Bacilli;o__Lactobacillales;f__Lactobacillaceae;g__Lactobacillus;s__uncultured compost bacterium |
| EF603719.1.1492 | -0.64784 | 1 | Other | 2 | 0.0027% | connectors | Rare | d__Bacteria;p__Bacteroidetes;c__Bacteroidia;o__Bacteroidales;f__Bacteroidales S24-7 group;g__norank;s__uncultured bacterium |
| KC134362.1.1431 | -0.64784 | 1 | Other | 3 | 0.0027% | connectors | Rare | d__Bacteria;p__Proteobacteria;c__Gammaproteobacteria;o__Enterobacteriales;f__Enterobacteriaceae;g__Yersinia;s__Yersinia sp. HME8608 |
| AB991631.1.1448 | -0.81856 | 0.888889 | VI | 3 | 0.0027% | connectors | Rare | d__Bacteria;p__Proteobacteria;c__Betaproteobacteria;o__Burkholderiales;f__Oxalobacteraceae;g__Massilia;s__Janthinobacterium sp. MsC-10-4CB1-04 |
| KT029535.1.1459 | -0.16667 | 0.248889 | I | 15 | 0.0027% | peripheral-nodes | Rare | d__Bacteria;p__Proteobacteria;c__Gammaproteobacteria;o__Pseudomonadales;f__Pseudomonadaceae;g__Pseudomonas;s__uncultured bacterium |
| AZRA01000044.142.1659 | -0.65558 | 0 | III | 4 | 0.0027% | peripheral-nodes | Rare | d__Bacteria;p__Proteobacteria;c__Betaproteobacteria;o__Burkholderiales;f__Comamonadaceae;g__Sphaerotilus;s__Sphaerotilus natans subsp. natans DSM 6575 |
| KT023574.1.1460 | -0.76423 | 0 | III | 2 | 0.0027% | peripheral-nodes | Rare | d__Bacteria;p__Proteobacteria;c__Betaproteobacteria;o__Burkholderiales;f__Oxalobacteraceae;g__Duganella;s__Massilia sp. THG-SS12.3 |
| KM288700.1.1414 | -0.65555 | 1 | III | 2 | 0.0027% | connectors | Rare | d__Bacteria;p__Bacteroidetes;c__Sphingobacteriia;o__Sphingobacteriales;f__Sphingobacteriaceae;g__Mucilaginibacter;s__Mucilaginibacter vulcanisilvae |
| KJ808500.1.1480 | -0.81856 | 0 | IV | 1 | 0.0028% | peripheral-nodes | Rare | d__Bacteria;p__Firmicutes;c__Clostridia;o__Clostridiales;f__Clostridiaceae 1;g__Clostridium sensu stricto 1;s__uncultured bacterium |
| EU474127.1.1379 | -0.16667 | 0.137755 | IV | 14 | 0.0028% | peripheral-nodes | Rare | d__Bacteria;p__Firmicutes;c__Clostridia;o__Clostridiales;f__Clostridiaceae 1;g__Clostridium sensu stricto 1;s__uncultured bacterium |
| AY532551.1.1499 | -0.32964 | 0.173554 | VII | 11 | 0.0028% | peripheral-nodes | Rare | d__Bacteria;p__Proteobacteria;c__Gammaproteobacteria;o__Pseudomonadales;f__Pseudomonadaceae;g__Pseudomonas;s__uncultured bacterium |
| LMDJ01000021.2783.4322 | -0.61052 | 0.888889 | III | 3 | 0.0028% | connectors | Rare | d__Bacteria;p__Proteobacteria;c__Betaproteobacteria;o__Burkholderiales;f__Oxalobacteraceae;g__Janthinobacterium;s__Massilia sp. Root351 |
| LMYF01009029.3575.5082 | -0.81856 | 0 | III | 1 | 0.0028% | peripheral-nodes | Rare | d__Bacteria;p__Bacteroidetes;c__Flavobacteriia;o__Flavobacteriales;f__Cryomorphaceae;g__Fluviicola;s__Hypsibius dujardini |
| HQ800197.1.1426 | -0.54694 | 0 | IV | 6 | 0.0028% | peripheral-nodes | Rare | d__Bacteria;p__Firmicutes;c__Clostridia;o__Clostridiales;f__Clostridiaceae 1;g__Clostridium sensu stricto 1;s__uncultured organism |
| GU563822.1.1406 | -0.70991 | 0.4375 | VI | 4 | 0.0028% | peripheral-nodes | Rare | d__Bacteria;p__Proteobacteria;c__Gammaproteobacteria;o__Pseudomonadales;f__Pseudomonadaceae;g__Pseudomonas;s__Pseudomonas fluorescens |
| BCZE01000148.3884.5352 | -0.11234 | 0.128889 | III | 15 | 0.0028% | peripheral-nodes | Rare | d__Bacteria;p__Proteobacteria;c__Alphaproteobacteria;o__Sphingomonadales;f__Sphingomonadaceae;g__Novosphingobium;s__Novosphingobium rosa NBRC 15208 |
| CVQX01000010.90813.92342 | -0.81856 | 0 | VII | 1 | 0.0028% | peripheral-nodes | Rare | d__Bacteria;p__Firmicutes;c__Bacilli;o__Bacillales;f__Bacillaceae;g__Bacillus;s__Bacillus sp. SIT10 |
| HQ809662.1.1427 | -0.65555 | 1 | IV | 22 | 0.0028% | connectors | Rare | d__Bacteria;p__Firmicutes;c__Clostridia;o__Clostridiales;f__Clostridiaceae 1;g__Clostridium sensu stricto 1;s__uncultured organism |
| EU775436.1.1404 | -0.65555 | 1 | Other | 2 | 0.0028% | connectors | Rare | d__Bacteria;p__Firmicutes;c__Clostridia;o__Clostridiales;f__Clostridiaceae 1;g__Sarcina;s__uncultured bacterium |
| FN377712.1.1243 | -0.00369 | 0 | I | 16 | 0.0028% | peripheral-nodes | Rare | d__Bacteria;p__Proteobacteria;c__Gammaproteobacteria;o__Pseudomonadales;f__Pseudomonadaceae;g__Pseudomonas;s__Pseudomonas sp. RS-42 |
| FJ957843.1.1431 | -0.76423 | 0 | Other | 2 | 0.0028% | peripheral-nodes | Rare | d__Bacteria;p__Firmicutes;c__Clostridia;o__Clostridiales;f__Clostridiaceae 1;g__Clostridium sensu stricto 13;s__uncultured bacterium |
| EU523225.1.1465 | -0.81856 | 0.75 | Other | 2 | 0.0028% | connectors | Rare | d__Bacteria;p__Proteobacteria;c__Gammaproteobacteria;o__Enterobacteriales;f__Enterobacteriaceae;g__Yersinia;s__Yersinia enterocolitica subsp. enterocolitica |
| EU775640.1.1374 | -0.65555 | 1 | Other | 3 | 0.0028% | connectors | Rare | d__Bacteria;p__Firmicutes;c__Clostridia;o__Clostridiales;f__Clostridiaceae 1;g__Clostridium sensu stricto 1;s__uncultured bacterium |
| EU539071.1.1395 | -0.81856 | 0.75 | VI | 2 | 0.0028% | connectors | Rare | d__Bacteria;p__Proteobacteria;c__Gammaproteobacteria;o__Pseudomonadales;f__Pseudomonadaceae;g__Pseudomonas;s__uncultured bacterium |
| HQ114113.1.1514 | -0.65558 | 0 | Other | 4 | 0.0028% | peripheral-nodes | Rare | d__Bacteria;p__Acidobacteria;c__Blastocatellia;o__Blastocatellales;f__Blastocatellaceae (Subgroup 4);g__DS-100;s__uncultured bacterium |
| LN651143.1.1400 | -0.65555 | 1 | IV | 2 | 0.0028% | connectors | Rare | d__Bacteria;p__Proteobacteria;c__Gammaproteobacteria;o__Oceanospirillales;f__Halomonadaceae;g__Halomonas;s__Halomonas sulfidaeris |
| FJ880455.1.1488 | -0.81856 | 0.75 | Other | 2 | 0.0028% | connectors | Rare | d__Bacteria;p__Firmicutes;c__Erysipelotrichia;o__Erysipelotrichales;f__Erysipelotrichaceae;g__uncultured;s__uncultured bacterium |
| AZIG01004893.834.2330 | -0.65558 | 0 | I | 4 | 0.0029% | peripheral-nodes | Rare | d__Bacteria;p__Bacteroidetes;c__Flavobacteriia;o__Flavobacteriales;f__Flavobacteriaceae;g__Flavobacterium;s__marine sediment metagenome |
| KJ782866.1.1516 | -0.16667 | 0.248889 | III | 15 | 0.0029% | peripheral-nodes | Rare | d__Bacteria;p__Proteobacteria;c__Deltaproteobacteria;o__Oligoflexales;f__0319-6G20;g__norank;s__uncultured bacterium |
| HM247284.1.1357 | -0.76423 | 0 | Other | 2 | 0.0029% | peripheral-nodes | Rare | d__Bacteria;p__Proteobacteria;c__Gammaproteobacteria;o__Pseudomonadales;f__Pseudomonadaceae;g__Pseudomonas;s__uncultured bacterium |
| GBSO01019834.117.1630 | -0.70991 | 0.4375 | I | 4 | 0.0029% | peripheral-nodes | Rare | d__Bacteria;p__Proteobacteria;c__Betaproteobacteria;o__Burkholderiales;f__Comamonadaceae;g__Hydrogenophaga;s__Klebsormidium flaccidum |
| CXWJ01035424.14.1545 | -0.49261 | 0.234375 | Other | 8 | 0.0029% | peripheral-nodes | Rare | d__Bacteria;p__Acidobacteria;c__Subgroup 6;o__norank;f__norank;g__norank;s__wastewater metagenome |
| EU452902.1.1432 | -0.22099 | 0 | VIII | 12 | 0.0029% | peripheral-nodes | Rare | d__Bacteria;p__Firmicutes;c__Bacilli;o__Lactobacillales;f__Lactobacillaceae;g__Lactobacillus;s__uncultured bacterium |
| GAFH01001643.7.1510 | -0.65555 | 1 | Other | 1 | 0.0029% | connectors | Rare | d__Bacteria;p__Bacteroidetes;c__Bacteroidia;o__Bacteroidales;f__Porphyromonadaceae;g__Paludibacter;s__Paratrimastix pyriformis |
| KT369699.1.1424 | -0.65555 | 1 | X | 1 | 0.0029% | connectors | Rare | d__Bacteria;p__Proteobacteria;c__Gammaproteobacteria;o__Pseudomonadales;f__Pseudomonadaceae;g__Pseudomonas;s__bacterium ASP20 |
| AKNC01000029.41468.42992 | -0.70991 | 0.4375 | IX | 4 | 0.0029% | peripheral-nodes | Rare | d__Bacteria;p__Proteobacteria;c__Gammaproteobacteria;o__Enterobacteriales;f__Enterobacteriaceae;g__Escherichia-Shigella;s__Shigella sonnei 3226-85 |
| HE582462.1.1498 | -0.65558 | 0.36 | IX | 5 | 0.0029% | peripheral-nodes | Rare | d__Bacteria;p__Proteobacteria;c__Gammaproteobacteria;o__Enterobacteriales;f__Enterobacteriaceae;g__Escherichia-Shigella;s__uncultured bacterium |
| KC819123.1.1291 | -0.65555 | 1 | IX | 7 | 0.0029% | connectors | Rare | d__Bacteria;p__Proteobacteria;c__Gammaproteobacteria;o__Enterobacteriales;f__Enterobacteriaceae;g__Escherichia-Shigella;s__Escherichia sp. 68 |
| FJ605265.1.1398 | -0.27532 | 0.284024 | III | 13 | 0.0030% | peripheral-nodes | Rare | d__Bacteria;p__Proteobacteria;c__Alphaproteobacteria;o__Sphingomonadales;f__Sphingomonadaceae;g__Sphingomonas;s__Sphingomonas sp. DCY40 |
| KC311567.1.1447 | -0.76423 | 0 | Other | 2 | 0.0030% | peripheral-nodes | Rare | d__Bacteria;p__Actinobacteria;c__Actinobacteria;o__Micrococcales;f__Micrococcaceae;g__Pseudarthrobacter;s__Arthrobacter sp. PF1T3 |
| KP899232.1.1343 | -0.22099 | 0.265306 | III | 14 | 0.0030% | peripheral-nodes | Rare | d__Bacteria;p__Bacteroidetes;c__Sphingobacteriia;o__Sphingobacteriales;f__Sphingobacteriaceae;g__Mucilaginibacter;s__Mucilaginibacter sp. S20-104 |
| FJ269055.1.1501 | -0.22099 | 0.147929 | I | 13 | 0.0030% | peripheral-nodes | Rare | d__Bacteria;p__Proteobacteria;c__Gammaproteobacteria;o__Pseudomonadales;f__Pseudomonadaceae;g__Pseudomonas;s__iron-reducing bacterium enrichment culture clone HN26 |
| JN792364.1.1408 | -0.65555 | 1 | Other | 1 | 0.0030% | connectors | Rare | d__Bacteria;p__Firmicutes;c__Clostridia;o__Clostridiales;f__Clostridiaceae 1;g__Clostridium sensu stricto 1;s__uncultured Clostridium sp. |
| EU469647.1.1396 | -0.16667 | 0 | I | 13 | 0.0030% | peripheral-nodes | Rare | d__Bacteria;p__Proteobacteria;c__Gammaproteobacteria;o__Pseudomonadales;f__Pseudomonadaceae;g__Pseudomonas;s__uncultured bacterium |
| AB013827.1.1502 | -0.65558 | 0 | I | 4 | 0.0030% | peripheral-nodes | Rare | d__Bacteria;p__Proteobacteria;c__Gammaproteobacteria;o__Pseudomonadales;f__Pseudomonadaceae;g__Pseudomonas;s__Pseudomonas sp. |
| EU470758.1.1381 | -0.76423 | 0 | Other | 2 | 0.0030% | peripheral-nodes | Rare | d__Bacteria;p__Firmicutes;c__Clostridia;o__Clostridiales;f__Clostridiaceae 1;g__Clostridium sensu stricto 1;s__uncultured bacterium |
| EF154242.1.1424 | -0.81856 | 0 | Other | 1 | 0.0030% | peripheral-nodes | Rare | d__Bacteria;p__Actinobacteria;c__Actinobacteria;o__Micrococcales;f__Micrococcaceae;g__Arthrobacter;s__Arthrobacter citreus |
| KT767683.1.1422 | -0.27532 | 0.159722 | VII | 12 | 0.0030% | peripheral-nodes | Rare | d__Bacteria;p__Proteobacteria;c__Gammaproteobacteria;o__Pseudomonadales;f__Pseudomonadaceae;g__Pseudomonas;s__Pseudomonas fragi |
| JRHH01000003.140784.142303 | -0.8765 | 1 | III | 12 | 0.0030% | connectors | Rare | d__Bacteria;p__Bacteroidetes;c__Flavobacteriia;o__Flavobacteriales;f__Flavobacteriaceae;g__Flavobacterium;s__Flavobacterium aquatile LMG 4008 |
| JQ977551.1.1387 | -0.00369 | 0.209877 | III | 18 | 0.0031% | peripheral-nodes | Rare | d__Bacteria;p__Proteobacteria;c__Alphaproteobacteria;o__Rhizobiales;f__Hyphomicrobiaceae;g__Devosia;s__Devosia sp. Bma11 |
| EU778421.1.1389 | -0.76423 | 0.555556 | Other | 3 | 0.0031% | peripheral-nodes | Rare | d__Bacteria;p__Proteobacteria;c__Gammaproteobacteria;o__Enterobacteriales;f__Enterobacteriaceae;g__Escherichia-Shigella;s__uncultured bacterium |
| GQ448226.1.1394 | -0.38396 | 0 | X | 9 | 0.0031% | peripheral-nodes | Rare | d__Bacteria;p__Proteobacteria;c__Gammaproteobacteria;o__Pseudomonadales;f__Moraxellaceae;g__Psychrobacter;s__uncultured bacterium |
| LN570801.1.1384 | -0.76423 | 0.555556 | Other | 3 | 0.0031% | peripheral-nodes | Rare | d__Bacteria;p__Proteobacteria;c__Deltaproteobacteria;o__Myxococcales;f__Sandaracinaceae;g__uncultured;s__uncultured bacterium |
| AIHK01000032.2221.3748 | -0.76423 | 0 | IX | 2 | 0.0031% | peripheral-nodes | Rare | d__Bacteria;p__Proteobacteria;c__Gammaproteobacteria;o__Enterobacteriales;f__Enterobacteriaceae;g__Escherichia-Shigella;s__Escherichia coli DEC14A |
| KM105816.1.1391 | -0.81856 | 0 | Other | 1 | 0.0031% | peripheral-nodes | Rare | d__Bacteria;p__Proteobacteria;c__Gammaproteobacteria;o__Enterobacteriales;f__Enterobacteriaceae;g__Escherichia-Shigella;s__uncultured bacterium |
| KP318449.1.1472 | -0.81856 | 0 | III | 1 | 0.0031% | peripheral-nodes | Rare | d__Bacteria;p__Proteobacteria;c__Betaproteobacteria;o__Burkholderiales;f__Alcaligenaceae;g__Verticia;s__Achromobacter pulmonis |
| HM265402.1.1358 | -0.43829 | 0 | I | 8 | 0.0032% | peripheral-nodes | Rare | d__Bacteria;p__Proteobacteria;c__Gammaproteobacteria;o__Pseudomonadales;f__Pseudomonadaceae;g__Pseudomonas;s__uncultured bacterium |
| JF690870.1.1452 | -0.81856 | 0 | IX | 1 | 0.0032% | peripheral-nodes | Rare | d__Bacteria;p__Proteobacteria;c__Gammaproteobacteria;o__Enterobacteriales;f__Enterobacteriaceae;g__Escherichia-Shigella;s__Cronobacter sakazakii |
| GQ871729.1.1504 | -0.81856 | 0 | Other | 1 | 0.0032% | peripheral-nodes | Rare | d__Bacteria;p__Proteobacteria;c__Gammaproteobacteria;o__Pseudomonadales;f__Pseudomonadaceae;g__Pseudomonas;s__uncultured bacterium |
| AYZE01000006.82.1620 | -0.76423 | 0 | Other | 2 | 0.0032% | peripheral-nodes | Rare | d__Bacteria;p__Firmicutes;c__Bacilli;o__Lactobacillales;f__Lactobacillaceae;g__Lactobacillus;s__Lactobacillus cacaonum DSM 21116 |
| AY689062.1.1490 | -0.81856 | 0 | Other | 1 | 0.0032% | peripheral-nodes | Rare | d__Bacteria;p__Proteobacteria;c__Gammaproteobacteria;o__Enterobacteriales;f__Enterobacteriaceae;g__Enterobacter;s__Enterobacter sp. 9B_2 |
| JQ184858.1.1342 | -0.64784 | 1 | V | 2 | 0.0032% | connectors | Rare | d__Bacteria;p__Firmicutes;c__Clostridia;o__Clostridiales;f__Clostridiaceae 1;g__Clostridium sensu stricto 1;s__uncultured bacterium |
| GQ379548.1.1274 | -0.81856 | 0.75 | VI | 2 | 0.0032% | connectors | Rare | d__Bacteria;p__Proteobacteria;c__Betaproteobacteria;o__Methylophilales;f__Methylophilaceae;g__Methylophilus;s__uncultured bacterium |
| KM021092.1.1313 | -0.38396 | 0.330579 | I | 11 | 0.0032% | peripheral-nodes | Rare | d__Bacteria;p__Bacteroidetes;c__Flavobacteriia;o__Flavobacteriales;f__Flavobacteriaceae;g__Flavobacterium;s__Flavobacterium sp. 127H |
| FJ849530.1.1404 | -0.81856 | 0.75 | III | 2 | 0.0032% | connectors | Rare | d__Bacteria;p__Proteobacteria;c__Alphaproteobacteria;o__Rhizobiales;f__Hyphomicrobiaceae;g__Devosia;s__uncultured bacterium |
| HQ741332.1.1445 | -0.00369 | 0.209877 | IV | 18 | 0.0032% | peripheral-nodes | Rare | d__Bacteria;p__Firmicutes;c__Clostridia;o__Clostridiales;f__Clostridiaceae 1;g__Clostridium sensu stricto 1;s__uncultured bacterium |
| HQ799925.1.1432 | -0.70991 | 0 | IV | 3 | 0.0033% | peripheral-nodes | Rare | d__Bacteria;p__Firmicutes;c__Clostridia;o__Clostridiales;f__Clostridiaceae 1;g__Clostridium sensu stricto 1;s__uncultured organism |
| KF256006.1.1527 | -0.76423 | 0 | Other | 2 | 0.0033% | peripheral-nodes | Rare | d__Bacteria;p__Firmicutes;c__Bacilli;o__Lactobacillales;f__Lactobacillaceae;g__Lactobacillus;s__uncultured bacterium |
| EF212892.1.1403 | -0.81856 | 0 | Other | 1 | 0.0033% | peripheral-nodes | Rare | d__Bacteria;p__Firmicutes;c__Bacilli;o__Bacillales;f__Paenibacillaceae;g__Paenibacillus;s__Paenibacillus urinalis |
| JN082730.1.1437 | -0.81856 | 0 | Other | 1 | 0.0033% | peripheral-nodes | Rare | d__Bacteria;p__Proteobacteria;c__Gammaproteobacteria;o__Enterobacteriales;f__Enterobacteriaceae;g__Pantoea;s__Pantoea sp. MR8 |
| FQ659571.1.1334 | -0.49261 | 0.234375 | Other | 8 | 0.0033% | peripheral-nodes | Rare | d__Bacteria;p__Chloroflexi;c__Thermomicrobia;o__JG30-KF-CM45;f__norank;g__norank;s__uncultured soil bacterium |
| JQ974027.1.1737 | -0.16667 | 0 | I | 13 | 0.0033% | peripheral-nodes | Rare | d__Bacteria;p__Proteobacteria;c__Gammaproteobacteria;o__Pseudomonadales;f__Pseudomonadaceae;g__Pseudomonas;s__Pseudomonas fluorescens |
| HQ661297.1.1434 | -0.70991 | 0 | Other | 3 | 0.0033% | peripheral-nodes | Rare | d__Bacteria;p__Firmicutes;c__Clostridia;o__Clostridiales;f__Clostridiaceae 1;g__Clostridium sensu stricto 1;s__uncultured bacterium |
| KU230002.1.1282 | -0.81856 | 0 | VI | 1 | 0.0033% | peripheral-nodes | Rare | d__Bacteria;p__Proteobacteria;c__Gammaproteobacteria;o__Pseudomonadales;f__Pseudomonadaceae;g__Pseudomonas;s__Pseudomonas sp. CAWT13 |
| AB991050.1.1411 | -0.70991 | 0 | V | 3 | 0.0034% | peripheral-nodes | Rare | d__Bacteria;p__Proteobacteria;c__Alphaproteobacteria;o__Rhizobiales;f__1174-901-12;g__norank;s__uncultured bacterium |
| JF135633.1.1359 | -0.65558 | 0.36 | VI | 5 | 0.0034% | peripheral-nodes | Rare | d__Bacteria;p__Proteobacteria;c__Gammaproteobacteria;o__Pseudomonadales;f__Pseudomonadaceae;g__Pseudomonas;s__uncultured bacterium |
| EU473897.1.1381 | -0.87288 | 1 | V | 1 | 0.0034% | connectors | Rare | d__Bacteria;p__Firmicutes;c__Clostridia;o__Clostridiales;f__Clostridiaceae 1;g__Clostridium sensu stricto 1;s__uncultured bacterium |
| FJ894339.1.1358 | -0.32964 | 0 | I | 10 | 0.0034% | peripheral-nodes | Rare | d__Bacteria;p__Proteobacteria;c__Gammaproteobacteria;o__Pseudomonadales;f__Pseudomonadaceae;g__Pseudomonas;s__uncultured bacterium |
| FQ659858.2.1373 | -0.70991 | 0.4375 | Other | 4 | 0.0034% | peripheral-nodes | Rare | d__Bacteria;p__Proteobacteria;c__Deltaproteobacteria;o__Myxococcales;f__Sandaracinaceae;g__uncultured;s__uncultured soil bacterium |
| LC076730.1.1422 | -0.76423 | 0 | V | 2 | 0.0034% | peripheral-nodes | Rare | d__Bacteria;p__Acidobacteria;c__Acidobacteria;o__Acidobacteriales;f__Acidobacteriaceae (Subgroup 1);g__Granulicella;s__uncultured bacterium |
| JX097001.1.1450 | -0.60126 | 0.489796 | III | 7 | 0.0034% | peripheral-nodes | Rare | d__Bacteria;p__Bacteroidetes;c__Flavobacteriia;o__Flavobacteriales;f__Flavobacteriaceae;g__Flavobacterium;s__Flavobacterium sp. C1206 |
| EU474114.1.1389 | 0.050629 | 0.108025 | IV | 18 | 0.0034% | peripheral-nodes | Rare | d__Bacteria;p__Firmicutes;c__Clostridia;o__Clostridiales;f__Clostridiaceae 1;g__Clostridium sensu stricto 1;s__uncultured bacterium |
| HQ741239.1.1415 | 0.648195 | 0.067776 | IV | 29 | 0.0034% | peripheral-nodes | Rare | d__Bacteria;p__Firmicutes;c__Clostridia;o__Clostridiales;f__Clostridiaceae 1;g__Clostridium sensu stricto 1;s__uncultured bacterium |
| GQ069622.1.1358 | -0.65558 | 0.36 | VI | 5 | 0.0034% | peripheral-nodes | Rare | d__Bacteria;p__Proteobacteria;c__Gammaproteobacteria;o__Pseudomonadales;f__Pseudomonadaceae;g__Pseudomonas;s__uncultured bacterium |
| AKNF01001123.2375.3897 | -0.70991 | 0.4375 | IX | 4 | 0.0034% | peripheral-nodes | Rare | d__Bacteria;p__Proteobacteria;c__Gammaproteobacteria;o__Enterobacteriales;f__Enterobacteriaceae;g__Escherichia-Shigella;s__Shigella flexneri 1235-66 |
| EU534622.1.1395 | -0.54694 | 0 | I | 6 | 0.0035% | peripheral-nodes | Rare | d__Bacteria;p__Proteobacteria;c__Gammaproteobacteria;o__Pseudomonadales;f__Pseudomonadaceae;g__Pseudomonas;s__uncultured bacterium |
| AB043868.1.1537 | -0.81856 | 0 | Other | 1 | 0.0035% | peripheral-nodes | Rare | d__Bacteria;p__Firmicutes;c__Bacilli;o__Bacillales;f__Paenibacillaceae;g__Paenibacillus;s__Paenibacillus sp. 7-5 |
| DQ816773.1.1442 | -0.49261 | 0 | Other | 7 | 0.0035% | peripheral-nodes | Rare | d__Bacteria;p__Firmicutes;c__Clostridia;o__Clostridiales;f__Clostridiaceae 1;g__Clostridium sensu stricto 1;s__uncultured bacterium |
| JQ010853.1.1504 | -0.65555 | 1 | VI | 3 | 0.0035% | connectors | Rare | d__Bacteria;p__Proteobacteria;c__Gammaproteobacteria;o__Pseudomonadales;f__Pseudomonadaceae;g__Pseudomonas;s__Pseudomonas sp. CRF3-Ps-2 |
| LFLU01000036.3737.5249 | 0.050629 | 0.2775 | I | 20 | 0.0035% | peripheral-nodes | Rare | d__Bacteria;p__Proteobacteria;c__Betaproteobacteria;o__Burkholderiales;f__Oxalobacteraceae;g__Herbaspirillum;s__Herbaspirillum rhizosphaerae |
| JACT01000002.454477.455967 | -0.54694 | 0.265306 | III | 7 | 0.0035% | peripheral-nodes | Rare | d__Bacteria;p__Proteobacteria;c__Alphaproteobacteria;o__Sphingomonadales;f__Sphingomonadaceae;g__Sphingobium;s__Sphingobium czechense LL01 |
| JN867430.1.1478 | -0.65558 | 0 | Other | 4 | 0.0035% | peripheral-nodes | Rare | d__Bacteria;p__Firmicutes;c__Clostridia;o__Clostridiales;f__Clostridiaceae 1;g__Clostridium sensu stricto 1;s__uncultured bacterium |
| DQ318861.1.1491 | -0.65558 | 0 | VI | 4 | 0.0036% | peripheral-nodes | Rare | d__Bacteria;p__Proteobacteria;c__Gammaproteobacteria;o__Pseudomonadales;f__Pseudomonadaceae;g__Pseudomonas;s__Pseudomonas syringae |
| EF532776.1.1348 | -0.65555 | 1 | Other | 1 | 0.0036% | connectors | Rare | d__Bacteria;p__Firmicutes;c__Clostridia;o__Clostridiales;f__Clostridiaceae 1;g__Clostridium sensu stricto 1;s__uncultured bacterium |
| EU473171.1.1378 | -0.54694 | 0 | IV | 6 | 0.0036% | peripheral-nodes | Rare | d__Bacteria;p__Firmicutes;c__Clostridia;o__Clostridiales;f__Clostridiaceae 1;g__Clostridium sensu stricto 1;s__uncultured bacterium |
| AIGA01000042.544051.545581 | -0.70991 | 0.4375 | IX | 4 | 0.0036% | peripheral-nodes | Rare | d__Bacteria;p__Proteobacteria;c__Gammaproteobacteria;o__Enterobacteriales;f__Enterobacteriaceae;g__Escherichia-Shigella;s__Escherichia coli DEC7A |
| KM979383.1.1422 | -0.76423 | 0 | V | 2 | 0.0036% | peripheral-nodes | Rare | d__Bacteria;p__Acidobacteria;c__Acidobacteria;o__Acidobacteriales;f__Acidobacteriaceae (Subgroup 1);g__Granulicella;s__Granulicella sp. 5B5 |
| GQ868409.1.1439 | 0.104953 | 0 | IV | 18 | 0.0036% | peripheral-nodes | Rare | d__Bacteria;p__Firmicutes;c__Clostridia;o__Clostridiales;f__Clostridiaceae 1;g__Clostridium sensu stricto 1;s__uncultured Clostridium sp. |
| ASVH01000027.1.1264 | -0.81856 | 0 | IX | 1 | 0.0037% | peripheral-nodes | Rare | d__Bacteria;p__Proteobacteria;c__Gammaproteobacteria;o__Enterobacteriales;f__Enterobacteriaceae;g__Escherichia-Shigella;s__Escherichia coli KTE103 |
| EU473373.1.1384 | -0.70991 | 0 | Other | 3 | 0.0037% | peripheral-nodes | Rare | d__Bacteria;p__Firmicutes;c__Clostridia;o__Clostridiales;f__Clostridiaceae 1;g__Clostridium sensu stricto 1;s__uncultured bacterium |
| FJ937929.1.1393 | -0.32964 | 0 | I | 10 | 0.0038% | peripheral-nodes | Rare | d__Bacteria;p__Proteobacteria;c__Gammaproteobacteria;o__Pseudomonadales;f__Pseudomonadaceae;g__Pseudomonas;s__Pseudomonas sp. LS227 |
| HQ176105.1.1471 | 0.539546 | 0.072702 | IV | 27 | 0.0038% | peripheral-nodes | Rare | d__Bacteria;p__Firmicutes;c__Clostridia;o__Clostridiales;f__Clostridiaceae 1;g__Clostridium sensu stricto 1;s__uncultured bacterium |
| EU473921.1.1384 | -0.65555 | 1 | IV | 5 | 0.0038% | connectors | Rare | d__Bacteria;p__Firmicutes;c__Clostridia;o__Clostridiales;f__Clostridiaceae 1;g__Clostridium sensu stricto 1;s__uncultured bacterium |
| LK392831.1.1375 | -0.54694 | 0.4375 | III | 8 | 0.0038% | peripheral-nodes | Rare | d__Bacteria;p__Bacteroidetes;c__Flavobacteriia;o__Flavobacteriales;f__Cryomorphaceae;g__Fluviicola;s__uncultured bacterium |
| AJ586620.1.1482 | -0.81856 | 0 | Other | 1 | 0.0038% | peripheral-nodes | Rare | d__Bacteria;p__Proteobacteria;c__Gammaproteobacteria;o__Enterobacteriales;f__Enterobacteriaceae;g__Erwinia;s__Candidatus Erwinia dacicola |
| EU775644.1.1361 | -0.76423 | 0 | IV | 2 | 0.0038% | peripheral-nodes | Rare | d__Bacteria;p__Firmicutes;c__Clostridia;o__Clostridiales;f__Clostridiaceae 1;g__Clostridium sensu stricto 1;s__uncultured bacterium |
| JN656286.1.1496 | -0.22099 | 0 | I | 12 | 0.0038% | peripheral-nodes | Rare | d__Bacteria;p__Proteobacteria;c__Gammaproteobacteria;o__Pseudomonadales;f__Pseudomonadaceae;g__Pseudomonas;s__Pseudomonas fluorescens |
| JUHU01000040.4332.5792 | 0.811167 | 0.21551 | III | 35 | 0.0038% | peripheral-nodes | Rare | d__Bacteria;p__Proteobacteria;c__Alphaproteobacteria;o__Rhizobiales;f__Rhizobiaceae;g__Rhizobium;s__Rhizobium leguminosarum |
| JQ410865.1.1530 | -0.87288 | 1 | II | 1 | 0.0038% | connectors | Rare | d__Bacteria;p__Proteobacteria;c__Gammaproteobacteria;o__Enterobacteriales;f__Enterobacteriaceae;g__Serratia;s__uncultured bacterium |
| KC854934.1.1444 | -0.76423 | 0 | I | 2 | 0.0039% | peripheral-nodes | Rare | d__Bacteria;p__Bacteroidetes;c__Sphingobacteriia;o__Sphingobacteriales;f__Sphingobacteriaceae;g__Pedobacter;s__Pedobacter nyackensis |
| EU775669.1.1323 | 0.702519 | 0.065556 | IV | 30 | 0.0039% | peripheral-nodes | Rare | d__Bacteria;p__Firmicutes;c__Clostridia;o__Clostridiales;f__Clostridiaceae 1;g__Clostridium sensu stricto 1;s__uncultured bacterium |
| EU537227.1.1395 | -0.27532 | 0 | VII | 11 | 0.0039% | peripheral-nodes | Rare | d__Bacteria;p__Proteobacteria;c__Gammaproteobacteria;o__Pseudomonadales;f__Pseudomonadaceae;g__Pseudomonas;s__uncultured bacterium |
| DQ833324.1.1534 | 0.159278 | 0.0975 | I | 20 | 0.0039% | peripheral-nodes | Rare | d__Bacteria;p__Proteobacteria;c__Gammaproteobacteria;o__Pseudomonadales;f__Pseudomonadaceae;g__Pseudomonas;s__uncultured bacterium |
| HQ743788.1.1298 | -0.76423 | 0 | IV | 2 | 0.0039% | peripheral-nodes | Rare | d__Bacteria;p__Firmicutes;c__Clostridia;o__Clostridiales;f__Clostridiaceae 1;g__Clostridium sensu stricto 1;s__uncultured organism |
| FJ975811.1.1357 | -0.16667 | 0 | VIII | 13 | 0.0039% | peripheral-nodes | Rare | d__Bacteria;p__Firmicutes;c__Bacilli;o__Lactobacillales;f__Lactobacillaceae;g__Lactobacillus;s__uncultured Lactobacillaceae bacterium |
| HQ120445.1.1508 | -0.49261 | 0.234375 | Other | 8 | 0.0039% | peripheral-nodes | Rare | d__Bacteria;p__Proteobacteria;c__Gammaproteobacteria;o__Xanthomonadales;f__Xanthomonadaceae;g__Tahibacter;s__uncultured bacterium |
| KJ540691.2.1398 | -0.87288 | 1 | Other | 1 | 0.0039% | connectors | Rare | d__Bacteria;p__Bacteroidetes;c__Sphingobacteriia;o__Sphingobacteriales;f__env.OPS 17;g__norank;s__uncultured Bacteroidetes bacterium |
| AM930376.1.1492 | -0.65558 | 0.36 | V | 5 | 0.0040% | peripheral-nodes | Rare | d__Bacteria;p__Firmicutes;c__Clostridia;o__Clostridiales;f__Clostridiaceae 1;g__Clostridium sensu stricto 1;s__uncultured bacterium |
| LC120820.1.1436 | -0.05802 | 0.221453 | III | 17 | 0.0040% | peripheral-nodes | Rare | d__Bacteria;p__Bacteroidetes;c__Flavobacteriia;o__Flavobacteriales;f__Flavobacteriaceae;g__Flavobacterium;s__Flavobacterium sp. TMd3a3 |
| KF101424.1.1357 | -0.65558 | 0.555556 | VII | 6 | 0.0040% | peripheral-nodes | Rare | d__Bacteria;p__Proteobacteria;c__Gammaproteobacteria;o__Pseudomonadales;f__Pseudomonadaceae;g__Pseudomonas;s__uncultured bacterium |
| JPRJ01000062.9586.11114 | -0.27532 | 0.284024 | I | 13 | 0.0040% | peripheral-nodes | Rare | d__Bacteria;p__Bacteroidetes;c__Flavobacteriia;o__Flavobacteriales;f__Flavobacteriaceae;g__Chryseobacterium;s__Chryseobacterium piperi |
| JUGA01000068.3613.4907 | -0.76423 | 0 | V | 2 | 0.0040% | peripheral-nodes | Rare | d__Bacteria;p__Cyanobacteria;c__Chloroplast;o__norank;f__norank;g__norank;s__Coccomyxa sp. LA000219 |
| LGEK01000159.187.1688 | -0.65555 | 1 | III | 4 | 0.0040% | connectors | Rare | d__Bacteria;p__Bacteroidetes;c__Sphingobacteriia;o__Sphingobacteriales;f__Sphingobacteriaceae;g__Mucilaginibacter;s__Mucilaginibacter sp. L294 |
| AYMG01000035.3947.5448 | -0.00369 | 0.419501 | III | 21 | 0.0040% | peripheral-nodes | Rare | d__Bacteria;p__Bacteroidetes;c__Sphingobacteriia;o__Sphingobacteriales;f__Sphingobacteriaceae;g__Pedobacter;s__Sphingobacterium sp. H1ai |
| JX144697.1.1366 | -0.76423 | 0 | Other | 2 | 0.0041% | peripheral-nodes | Rare | d__Bacteria;p__Firmicutes;c__Bacilli;o__Bacillales;f__Paenibacillaceae;g__Paenibacillus;s__Paenibacillus taiwanensis |
| AB862892.1.1496 | 0.104953 | 0.19 | III | 20 | 0.0042% | peripheral-nodes | Rare | d__Bacteria;p__Bacteroidetes;c__Sphingobacteriia;o__Sphingobacteriales;f__env.OPS 17;g__norank;s__uncultured bacterium |
| EU470514.1.1399 | -1.19338 | 1 | Other | 1 | 0.0042% | connectors | Rare | d__Bacteria;p__Proteobacteria;c__Gammaproteobacteria;o__Enterobacteriales;f__Enterobacteriaceae;g__Escherichia-Shigella;s__uncultured bacterium |
| KM977829.1.1399 | 0.159278 | 0 | I | 19 | 0.0042% | peripheral-nodes | Rare | d__Bacteria;p__Proteobacteria;c__Gammaproteobacteria;o__Pseudomonadales;f__Pseudomonadaceae;g__Pseudomonas;s__Pseudomonas sp. LP31_L02 |
| HM204922.1.1448 | -0.16667 | 0.137755 | III | 14 | 0.0042% | peripheral-nodes | Rare | d__Bacteria;p__Bacteroidetes;c__Sphingobacteriia;o__Sphingobacteriales;f__Sphingobacteriaceae;g__Mucilaginibacter;s__Mucilaginibacter sp. QM49 |
| CEMM01316617.553.2054 | -0.70991 | 0 | III | 3 | 0.0042% | peripheral-nodes | Rare | d__Bacteria;p__Bacteroidetes;c__Sphingobacteriia;o__Sphingobacteriales;f__Sphingobacteriaceae;g__Mucilaginibacter;s__Hygrocybe conica |
| KC424692.1.1527 | -0.54694 | 0.265306 | Other | 7 | 0.0042% | peripheral-nodes | Rare | d__Bacteria;p__Proteobacteria;c__Gammaproteobacteria;o__Xanthomonadales;f__Xanthomonadales Incertae Sedis;g__uncultured;s__uncultured bacterium |
| FJ889557.1.1497 | -0.70991 | 0.64 | IV | 5 | 0.0042% | connectors | Rare | d__Bacteria;p__Proteobacteria;c__Gammaproteobacteria;o__Oceanospirillales;f__Halomonadaceae;g__Halomonas;s__Halomonas sp. BR055 |
| AQPX01000042.62.1623 | -0.64784 | 1 | Other | 1 | 0.0042% | connectors | Rare | d__Bacteria;p__Firmicutes;c__Bacilli;o__Bacillales;f__Planococcaceae;g__Lysinibacillus;s__Lysinibacillus sphaericus OT4b.31 |
| CXOM01000002.332084.333594 | 0.702519 | 0.272491 | I | 34 | 0.0043% | peripheral-nodes | Rare | d__Bacteria;p__Proteobacteria;c__Betaproteobacteria;o__Burkholderiales;f__Comamonadaceae;g__Limnohabitans;s__Limnohabitans sp. Rim11 |
| JF117025.1.1305 | -0.60126 | 0.305556 | V | 6 | 0.0043% | peripheral-nodes | Rare | d__Bacteria;p__Proteobacteria;c__Alphaproteobacteria;o__Rhizobiales;f__1174-901-12;g__norank;s__uncultured bacterium |
| HM341084.1.1343 | -0.70991 | 0.4375 | Other | 4 | 0.0043% | peripheral-nodes | Rare | d__Bacteria;p__Fibrobacteres;c__Fibrobacteria;o__Fibrobacterales;f__Fibrobacteraceae;g__uncultured;s__uncultured bacterium |
| JQ977531.1.1368 | 0.919815 | 0.245845 | III | 38 | 0.0043% | peripheral-nodes | Rare | d__Bacteria;p__Bacteroidetes;c__Sphingobacteriia;o__Sphingobacteriales;f__Sphingobacteriaceae;g__Pedobacter;s__Pedobacter sp. Bca39 |
| AJ288899.1.1371 | 1.028464 | 0.305556 | III | 42 | 0.0044% | peripheral-nodes | Rare | d__Bacteria;p__Proteobacteria;c__Deltaproteobacteria;o__Bdellovibrionales;f__Bacteriovoracaceae;g__Bacteriovorax;s__Bacteriovorax stolpii |
| AB911469.1.2879 | -0.76423 | 0 | Other | 2 | 0.0044% | peripheral-nodes | Rare | d__Bacteria;p__Firmicutes;c__Bacilli;o__Lactobacillales;f__Lactobacillaceae;g__Lactobacillus;s__Lactobacillus salivarius |
| HM248571.1.1358 | -0.22099 | 0 | I | 12 | 0.0044% | peripheral-nodes | Rare | d__Bacteria;p__Proteobacteria;c__Gammaproteobacteria;o__Pseudomonadales;f__Pseudomonadaceae;g__Pseudomonas;s__uncultured bacterium |
| HM295535.1.1352 | 1.463057 | 0.2604 | III | 50 | 0.0044% | peripheral-nodes | Rare | d__Bacteria;p__Proteobacteria;c__Betaproteobacteria;o__Burkholderiales;f__Oxalobacteraceae;g__Paucimonas;s__uncultured bacterium |
| KC108996.1.1244 | -0.76423 | 0 | II | 2 | 0.0044% | peripheral-nodes | Rare | d__Bacteria;p__Proteobacteria;c__Gammaproteobacteria;o__Enterobacteriales;f__Enterobacteriaceae;g__Yersinia;s__Yersinia sp. UA-JF4206 |
| EU774345.1.1370 | -0.76423 | 0 | IV | 2 | 0.0044% | peripheral-nodes | Rare | d__Bacteria;p__Firmicutes;c__Clostridia;o__Clostridiales;f__Lachnospiraceae;g__[Ruminococcus] torques group;s__uncultured bacterium |
| ACOQ01000017.19.1555 | -0.70991 | 0 | Other | 3 | 0.0044% | peripheral-nodes | Rare | d__Bacteria;p__Proteobacteria;c__Gammaproteobacteria;o__Pseudomonadales;f__Pseudomonadaceae;g__Pseudomonas;s__Pseudomonas sp. UK4 |
| FN667071.1.1468 | 0.376574 | 0.217456 | VII | 26 | 0.0044% | peripheral-nodes | Rare | d__Bacteria;p__Proteobacteria;c__Gammaproteobacteria;o__Pseudomonadales;f__Pseudomonadaceae;g__Pseudomonas;s__uncultured compost bacterium |
| EF552157.1.1423 | -0.70991 | 0 | VII | 3 | 0.0044% | peripheral-nodes | Rare | d__Bacteria;p__Proteobacteria;c__Gammaproteobacteria;o__Pseudomonadales;f__Pseudomonadaceae;g__Pseudomonas;s__Pseudomonas fluorescens |
| AJ289162.1.1486 | -0.65555 | 1 | III | 2 | 0.0044% | connectors | Rare | d__Bacteria;p__Proteobacteria;c__Gammaproteobacteria;o__Cellvibrionales;f__Cellvibrionaceae;g__Cellvibrio;s__Cellvibrio gandavensis |
| GQ133753.1.1376 | -1.19338 | 1 | V | 5 | 0.0045% | connectors | Rare | d__Bacteria;p__Firmicutes;c__Clostridia;o__Clostridiales;f__Clostridiaceae 1;g__Clostridium sensu stricto 1;s__uncultured bacterium |
| KC855481.1.1365 | -0.70991 | 0.4375 | I | 4 | 0.0045% | peripheral-nodes | Rare | d__Bacteria;p__Bacteroidetes;c__Sphingobacteriia;o__Sphingobacteriales;f__Sphingobacteriaceae;g__Pedobacter;s__Pedobacter boryungensis |
| EU460438.1.1398 | 0.159278 | 0.0975 | I | 20 | 0.0045% | peripheral-nodes | Rare | d__Bacteria;p__Proteobacteria;c__Gammaproteobacteria;o__Pseudomonadales;f__Pseudomonadaceae;g__Pseudomonas;s__uncultured bacterium |
| GQ136415.1.1208 | -0.76423 | 0.555556 | V | 3 | 0.0045% | peripheral-nodes | Rare | d__Bacteria;p__Firmicutes;c__Clostridia;o__Clostridiales;f__Clostridiaceae 1;g__Clostridium sensu stricto 1;s__uncultured bacterium |
| AB702756.1.1405 | -0.81856 | 0.75 | Other | 2 | 0.0045% | connectors | Rare | d__Bacteria;p__Bacteroidetes;c__Bacteroidia;o__Bacteroidales;f__Bacteroidales S24-7 group;g__norank;s__uncultured Bacteroidales bacterium |
| FJ682164.1.1450 | -0.81856 | 0.75 | Other | 2 | 0.0045% | connectors | Rare | d__Bacteria;p__Firmicutes;c__Clostridia;o__Clostridiales;f__Clostridiaceae 1;g__Clostridium sensu stricto 1;s__uncultured bacterium |
| KT215434.1.1399 | -0.76423 | 0 | II | 2 | 0.0046% | peripheral-nodes | Rare | d__Bacteria;p__Proteobacteria;c__Gammaproteobacteria;o__Enterobacteriales;f__Enterobacteriaceae;g__Serratia;s__Serratia marcescens |
| HQ741385.1.1426 | -0.00369 | 0 | IV | 16 | 0.0046% | peripheral-nodes | Rare | d__Bacteria;p__Firmicutes;c__Clostridia;o__Clostridiales;f__Clostridiaceae 1;g__Clostridium sensu stricto 1;s__uncultured bacterium |
| AJKX01000093.1447.2967 | -0.81856 | 0 | II | 1 | 0.0046% | peripheral-nodes | Rare | d__Bacteria;p__Proteobacteria;c__Gammaproteobacteria;o__Enterobacteriales;f__Enterobacteriaceae;g__Cronobacter;s__Cronobacter dublinensis subsp. lactaridi LMG 23825 |
| KF080460.1.1364 | -0.54694 | 0.265306 | IX | 7 | 0.0046% | peripheral-nodes | Rare | d__Bacteria;p__Proteobacteria;c__Gammaproteobacteria;o__Enterobacteriales;f__Enterobacteriaceae;g__Escherichia-Shigella;s__uncultured bacterium |
| EU474074.1.1383 | -0.76423 | 0 | Other | 2 | 0.0046% | peripheral-nodes | Rare | d__Bacteria;p__Firmicutes;c__Clostridia;o__Clostridiales;f__Clostridiaceae 1;g__Clostridium sensu stricto 1;s__uncultured bacterium |
| EU469655.1.1396 | -0.32964 | 0 | X | 10 | 0.0046% | peripheral-nodes | Rare | d__Bacteria;p__Proteobacteria;c__Gammaproteobacteria;o__Pseudomonadales;f__Pseudomonadaceae;g__Pseudomonas;s__uncultured bacterium |
| LMPZ01000003.219530.220994 | -0.87288 | 1 | III | 1 | 0.0047% | connectors | Rare | d__Bacteria;p__Proteobacteria;c__Alphaproteobacteria;o__Caulobacterales;f__Caulobacteraceae;g__Brevundimonas;s__Brevundimonas sp. Leaf363 |
| HQ755382.1.1429 | -0.87288 | 1 | Other | 1 | 0.0047% | connectors | Rare | d__Bacteria;p__Firmicutes;c__Clostridia;o__Clostridiales;f__Clostridiaceae 1;g__Clostridium sensu stricto 1;s__uncultured organism |
| KR189115.1.1248 | -0.65558 | 0 | II | 4 | 0.0047% | peripheral-nodes | Rare | d__Bacteria;p__Proteobacteria;c__Gammaproteobacteria;o__Enterobacteriales;f__Enterobacteriaceae;g__Escherichia-Shigella;s__Escherichia sp. UIWRF0965 |
| FJ975875.1.1374 | -0.22099 | 0 | VIII | 12 | 0.0047% | peripheral-nodes | Rare | d__Bacteria;p__Firmicutes;c__Bacilli;o__Lactobacillales;f__Lactobacillaceae;g__Lactobacillus;s__uncultured Lactobacillaceae bacterium |
| HQ111163.1.1491 | -0.00369 | 0.290859 | III | 19 | 0.0047% | peripheral-nodes | Rare | d__Bacteria;p__Bacteroidetes;c__Sphingobacteriia;o__Sphingobacteriales;f__env.OPS 17;g__norank;s__uncultured Bacteroidetes bacterium |
| HQ158674.1.1473 | -0.49261 | 0.234375 | Other | 8 | 0.0047% | peripheral-nodes | Rare | d__Bacteria;p__Gemmatimonadetes;c__Gemmatimonadetes;o__Gemmatimonadales;f__Gemmatimonadaceae;g__uncultured;s__uncultured bacterium |
| JQ066779.1.1349 | -0.81856 | 0 | II | 1 | 0.0048% | peripheral-nodes | Rare | d__Bacteria;p__Proteobacteria;c__Gammaproteobacteria;o__Enterobacteriales;f__Enterobacteriaceae;g__Serratia;s__Serratia marcescens |
| KF624752.1.1349 | -0.65558 | 0.36 | II | 5 | 0.0048% | peripheral-nodes | Rare | d__Bacteria;p__Proteobacteria;c__Gammaproteobacteria;o__Enterobacteriales;f__Enterobacteriaceae;g__Serratia;s__Serratia marcescens |
| KJ808499.1.1480 | -0.00369 | 0.114187 | IV | 17 | 0.0048% | peripheral-nodes | Rare | d__Bacteria;p__Firmicutes;c__Clostridia;o__Clostridiales;f__Clostridiaceae 1;g__Clostridium sensu stricto 1;s__uncultured bacterium |
| EU474010.1.1383 | -0.76423 | 0 | Other | 2 | 0.0048% | peripheral-nodes | Rare | d__Bacteria;p__Firmicutes;c__Clostridia;o__Clostridiales;f__Clostridiaceae 1;g__Clostridium sensu stricto 1;s__uncultured bacterium |
| KP638488.1.1411 | -0.81856 | 0 | Other | 1 | 0.0048% | peripheral-nodes | Rare | d__Bacteria;p__Firmicutes;c__Bacilli;o__Bacillales;f__Paenibacillaceae;g__Paenibacillus;s__Paenibacillus sp. M33 |
| KT369696.1.1375 | -0.60126 | 0 | X | 5 | 0.0048% | peripheral-nodes | Rare | d__Bacteria;p__Proteobacteria;c__Gammaproteobacteria;o__Pseudomonadales;f__Pseudomonadaceae;g__Pseudomonas;s__bacterium ASP7 |
| EU459317.1.1373 | 0.539546 | 0.137755 | IV | 28 | 0.0048% | peripheral-nodes | Rare | d__Bacteria;p__Firmicutes;c__Clostridia;o__Clostridiales;f__Clostridiaceae 1;g__Clostridium sensu stricto 1;s__uncultured bacterium |
| LN832022.1.1432 | -0.65558 | 0.555556 | I | 6 | 0.0049% | peripheral-nodes | Rare | d__Bacteria;p__Bacteroidetes;c__Sphingobacteriia;o__Sphingobacteriales;f__Sphingobacteriaceae;g__Pedobacter;s__Pedobacter sp. NA93 |
| JNQM01000002.414865.416394 | -0.81856 | 0 | II | 1 | 0.0049% | peripheral-nodes | Rare | d__Bacteria;p__Proteobacteria;c__Gammaproteobacteria;o__Enterobacteriales;f__Enterobacteriaceae;g__Escherichia-Shigella;s__Escherichia coli 2-222-05_S1_C3 |
| LEUJ01000014.228371.229900 | -0.65555 | 1 | II | 1 | 0.0049% | connectors | Rare | d__Bacteria;p__Proteobacteria;c__Gammaproteobacteria;o__Enterobacteriales;f__Enterobacteriaceae;g__Escherichia-Shigella;s__Escherichia coli |
| EU775591.1.1273 | -0.87288 | 1 | V | 1 | 0.0049% | connectors | Rare | d__Bacteria;p__Firmicutes;c__Bacilli;o__Lactobacillales;f__Streptococcaceae;g__Streptococcus;s__uncultured bacterium |
| HE576008.1.1485 | -0.38396 | 0.330579 | II | 11 | 0.0049% | peripheral-nodes | Rare | d__Bacteria;p__Proteobacteria;c__Gammaproteobacteria;o__Enterobacteriales;f__Enterobacteriaceae;g__Serratia;s__uncultured bacterium |
| FJ682831.1.1371 | 0.702519 | 0.065556 | IV | 30 | 0.0049% | peripheral-nodes | Rare | d__Bacteria;p__Firmicutes;c__Clostridia;o__Clostridiales;f__Clostridiaceae 1;g__Clostridium sensu stricto 1;s__uncultured bacterium |
| EU455354.1.1406 | -0.16667 | 0 | VIII | 13 | 0.0050% | peripheral-nodes | Rare | d__Bacteria;p__Firmicutes;c__Bacilli;o__Lactobacillales;f__Lactobacillaceae;g__Lactobacillus;s__uncultured bacterium |
| HQ880406.1.1446 | 0.050629 | 0.199446 | II | 19 | 0.0050% | peripheral-nodes | Rare | d__Bacteria;p__Proteobacteria;c__Gammaproteobacteria;o__Enterobacteriales;f__Enterobacteriaceae;g__Cronobacter;s__Cronobacter turicensis |
| JN091870.1.1475 | -0.49261 | 0.395062 | II | 9 | 0.0050% | peripheral-nodes | Rare | d__Bacteria;p__Proteobacteria;c__Gammaproteobacteria;o__Enterobacteriales;f__Enterobacteriaceae;g__Serratia;s__Serratia sp. J145 |
| HQ811059.1.1417 | -0.49261 | 0.234375 | Other | 8 | 0.0051% | peripheral-nodes | Rare | d__Bacteria;p__Firmicutes;c__Clostridia;o__Clostridiales;f__Clostridiaceae 1;g__Clostridium sensu stricto 1;s__uncultured organism |
| JX006255.1.1435 | -0.76423 | 0 | IX | 2 | 0.0051% | peripheral-nodes | Rare | d__Bacteria;p__Proteobacteria;c__Gammaproteobacteria;o__Enterobacteriales;f__Enterobacteriaceae;g__Escherichia-Shigella;s__bacterium NLAE-zl-H4 |
| EU470739.1.1321 | -0.49261 | 0.395062 | II | 9 | 0.0051% | peripheral-nodes | Rare | d__Bacteria;p__Proteobacteria;c__Gammaproteobacteria;o__Enterobacteriales;f__Enterobacteriaceae;g__Escherichia-Shigella;s__uncultured bacterium |
| KF079674.1.1363 | -0.60126 | 0.489796 | II | 7 | 0.0051% | peripheral-nodes | Rare | d__Bacteria;p__Proteobacteria;c__Gammaproteobacteria;o__Enterobacteriales;f__Enterobacteriaceae;g__Serratia;s__uncultured bacterium |
| HQ721305.1.1397 | 0.376574 | 0.1536 | I | 25 | 0.0051% | peripheral-nodes | Rare | d__Bacteria;p__Proteobacteria;c__Gammaproteobacteria;o__Pseudomonadales;f__Pseudomonadaceae;g__Pseudomonas;s__uncultured bacterium |
| AB681099.1.1480 | -0.81856 | 0 | Other | 1 | 0.0051% | peripheral-nodes | Rare | d__Bacteria;p__Firmicutes;c__Bacilli;o__Bacillales;f__Paenibacillaceae;g__Paenibacillus;s__Paenibacillus durus |
| HM321947.1.1358 | 0.485222 | 0.142661 | I | 27 | 0.0052% | peripheral-nodes | Rare | d__Bacteria;p__Proteobacteria;c__Gammaproteobacteria;o__Pseudomonadales;f__Pseudomonadaceae;g__Pseudomonas;s__uncultured bacterium |
| AM491369.1.1503 | 1.028464 | 0.305556 | I | 42 | 0.0052% | peripheral-nodes | Rare | d__Bacteria;p__Bacteroidetes;c__Sphingobacteriia;o__Sphingobacteriales;f__Sphingobacteriaceae;g__Pedobacter;s__Pedobacter westerhofensis |
| EU729366.1.1431 | 0.648195 | 0.234375 | III | 32 | 0.0052% | peripheral-nodes | Rare | d__Bacteria;p__Bacteroidetes;c__Sphingobacteriia;o__Sphingobacteriales;f__Sphingobacteriaceae;g__Mucilaginibacter;s__Mucilaginibacter ximonensis |
| KF071691.1.1363 | -0.54694 | 0.265306 | II | 7 | 0.0053% | peripheral-nodes | Rare | d__Bacteria;p__Proteobacteria;c__Gammaproteobacteria;o__Enterobacteriales;f__Enterobacteriaceae;g__Serratia;s__uncultured bacterium |
| EU057878.1.1398 | -0.49261 | 0.595041 | III | 11 | 0.0053% | peripheral-nodes | Rare | d__Bacteria;p__Proteobacteria;c__Betaproteobacteria;o__Burkholderiales;f__Oxalobacteraceae;g__Massilia;s__Oxalobacteraceae bacterium CH37-4 |
| HK240580.1.1481 | -0.76423 | 0 | I | 2 | 0.0053% | peripheral-nodes | Rare | d__Bacteria;p__Bacteroidetes;c__Flavobacteriia;o__Flavobacteriales;f__Flavobacteriaceae;g__Flavobacterium;s__unidentified |
| AB696044.1.1436 | -0.54694 | 0 | Other | 6 | 0.0053% | peripheral-nodes | Rare | d__Bacteria;p__Firmicutes;c__Clostridia;o__Clostridiales;f__Clostridiaceae 1;g__Clostridium sensu stricto 1;s__uncultured bacterium |
| HK555901.1.1470 | -0.54694 | 0 | Other | 6 | 0.0053% | peripheral-nodes | Rare | d__Bacteria;p__Firmicutes;c__Clostridia;o__Clostridiales;f__Clostridiaceae 1;g__Clostridium sensu stricto 1;s__unidentified |
| FM873470.1.1467 | -0.81856 | 0 | Other | 1 | 0.0053% | peripheral-nodes | Rare | d__Bacteria;p__Firmicutes;c__Clostridia;o__Clostridiales;f__Clostridiaceae 1;g__Clostridium sensu stricto 1;s__uncultured bacterium |
| HQ176186.1.1439 | -0.38396 | 0 | IV | 9 | 0.0053% | peripheral-nodes | Rare | d__Bacteria;p__Firmicutes;c__Clostridia;o__Clostridiales;f__Clostridiaceae 1;g__Clostridium sensu stricto 1;s__uncultured bacterium |
| APMI01029102.28.1530 | -0.87288 | 1 | I | 1 | 0.0053% | connectors | Rare | d__Bacteria;p__Actinobacteria;c__Actinobacteria;o__Micrococcales;f__Microbacteriaceae;g__Galbitalea;s__wastewater metagenome |
| EU539396.1.1395 | -0.00369 | 0.209877 | VII | 18 | 0.0054% | peripheral-nodes | Rare | d__Bacteria;p__Proteobacteria;c__Gammaproteobacteria;o__Pseudomonadales;f__Pseudomonadaceae;g__Pseudomonas;s__uncultured bacterium |
| HE974825.1.1502 | -0.54694 | 0.265306 | Other | 7 | 0.0055% | peripheral-nodes | Rare | d__Bacteria;p__Proteobacteria;c__Gammaproteobacteria;o__Xanthomonadales;f__Xanthomonadales Incertae Sedis;g__uncultured;s__uncultured Steroidobacter sp. |
| EU558694.1.1408 | -0.81856 | 0 | II | 1 | 0.0055% | peripheral-nodes | Rare | d__Bacteria;p__Proteobacteria;c__Gammaproteobacteria;o__Enterobacteriales;f__Enterobacteriaceae;g__Serratia;s__Serratia marcescens |
| AM183065.1.1437 | -0.81856 | 0 | Other | 1 | 0.0055% | peripheral-nodes | Rare | d__Bacteria;p__Firmicutes;c__Clostridia;o__Clostridiales;f__Clostridiaceae 1;g__Clostridium sensu stricto 1;s__uncultured bacterium |
| GQ138620.1.1378 | -0.76423 | 0.555556 | V | 3 | 0.0055% | peripheral-nodes | Rare | d__Bacteria;p__Firmicutes;c__Clostridia;o__Clostridiales;f__Clostridiaceae 1;g__Clostridium sensu stricto 1;s__uncultured bacterium |
| EU776680.1.1401 | -0.87288 | 1 | VIII | 1 | 0.0055% | connectors | Rare | d__Bacteria;p__Proteobacteria;c__Gammaproteobacteria;o__Enterobacteriales;f__Enterobacteriaceae;g__Escherichia-Shigella;s__uncultured bacterium |
| JN867392.1.1512 | 0.593871 | 0.133175 | I | 29 | 0.0056% | peripheral-nodes | Rare | d__Bacteria;p__Proteobacteria;c__Gammaproteobacteria;o__Pseudomonadales;f__Pseudomonadaceae;g__Pseudomonas;s__uncultured bacterium |
| LXTQ01000003.3712.5257 | -0.58867 | 0.997732 | III | 42 | 0.0056% | connectors | Rare | d__Bacteria;p__Proteobacteria;c__Betaproteobacteria;o__Methylophilales;f__Methylophilaceae;g__uncultured;s__Methylobacillus sp. MM2 |
| FJ748509.1.1498 | 0.485222 | 0.075444 | I | 26 | 0.0056% | peripheral-nodes | Rare | d__Bacteria;p__Proteobacteria;c__Gammaproteobacteria;o__Pseudomonadales;f__Pseudomonadaceae;g__Pseudomonas;s__Pseudomonas sp. BSw21401 |
| LC050335.1.1497 | -0.38396 | 0.19 | VII | 10 | 0.0056% | peripheral-nodes | Rare | d__Bacteria;p__Proteobacteria;c__Gammaproteobacteria;o__Pseudomonadales;f__Pseudomonadaceae;g__Pseudomonas;s__uncultured Pseudomonas sp. |
| KC734179.1.1441 | -0.32964 | 0.305556 | II | 12 | 0.0056% | peripheral-nodes | Rare | d__Bacteria;p__Proteobacteria;c__Gammaproteobacteria;o__Enterobacteriales;f__Enterobacteriaceae;g__Serratia;s__bacterium 14W324 |
| EU460278.1.1396 | -0.27532 | 0 | X | 11 | 0.0056% | peripheral-nodes | Rare | d__Bacteria;p__Proteobacteria;c__Gammaproteobacteria;o__Pseudomonadales;f__Pseudomonadaceae;g__Pseudomonas;s__uncultured bacterium |
| FJ950690.1.1448 | -0.49261 | 0 | X | 7 | 0.0056% | peripheral-nodes | Rare | d__Bacteria;p__Proteobacteria;c__Gammaproteobacteria;o__Pseudomonadales;f__Pseudomonadaceae;g__Pseudomonas;s__Pseudomonas psychrophila |
| GQ133849.1.1380 | -0.8765 | 1 | Other | 1 | 0.0057% | connectors | Rare | d__Bacteria;p__Firmicutes;c__Clostridia;o__Clostridiales;f__Clostridiaceae 1;g__Clostridium sensu stricto 1;s__uncultured bacterium |
| KP713417.1.1541 | -0.05802 | 0.221453 | II | 17 | 0.0057% | peripheral-nodes | Rare | d__Bacteria;p__Proteobacteria;c__Gammaproteobacteria;o__Enterobacteriales;f__Enterobacteriaceae;g__Serratia;s__uncultured bacterium |
| DD183453.1.1404 | -0.70991 | 0 | III | 3 | 0.0057% | peripheral-nodes | Rare | d__Bacteria;p__Bacteroidetes;c__Sphingobacteriia;o__Sphingobacteriales;f__Sphingobacteriaceae;g__Pedobacter;s__unidentified |
| JQ691696.1.1490 | -0.70991 | 0 | VI | 3 | 0.0057% | peripheral-nodes | Rare | d__Bacteria;p__Proteobacteria;c__Gammaproteobacteria;o__Pseudomonadales;f__Pseudomonadaceae;g__Pseudomonas;s__Pseudomonas fluorescens |
| LC132830.1.1355 | -0.61052 | 0.996094 | III | 16 | 0.0057% | connectors | Rare | d__Bacteria;p__Proteobacteria;c__Alphaproteobacteria;o__Caulobacterales;f__Caulobacteraceae;g__Brevundimonas;s__Brevundimonas sp. ICHIDE14 |
| AB976554.1.1526 | -0.32964 | 0 | VIII | 10 | 0.0057% | peripheral-nodes | Rare | d__Bacteria;p__Firmicutes;c__Bacilli;o__Lactobacillales;f__Lactobacillaceae;g__Lactobacillus;s__Lactobacillus gasseri |
| JUGR01000001.3427598.3429077 | -0.60126 | 0 | V | 5 | 0.0058% | peripheral-nodes | Rare | d__Bacteria;p__Acidobacteria;c__Acidobacteria;o__Acidobacteriales;f__Acidobacteriaceae (Subgroup 1);g__Terriglobus;s__Terriglobus sp. TAA 43 |
| AF227841.1.1383 | 0.539546 | 0.137755 | I | 28 | 0.0058% | peripheral-nodes | Rare | d__Bacteria;p__Proteobacteria;c__Gammaproteobacteria;o__Pseudomonadales;f__Pseudomonadaceae;g__Pseudomonas;s__Pseudomonas sp. 63596 |
| KU057022.1.1477 | -0.22099 | 0.147929 | II | 13 | 0.0058% | peripheral-nodes | Rare | d__Bacteria;p__Proteobacteria;c__Gammaproteobacteria;o__Enterobacteriales;f__Enterobacteriaceae;g__Serratia;s__Serratia sp. R27 |
| KC819114.1.1304 | -0.76423 | 0 | II | 2 | 0.0058% | peripheral-nodes | Rare | d__Bacteria;p__Proteobacteria;c__Gammaproteobacteria;o__Enterobacteriales;f__Enterobacteriaceae;g__Escherichia-Shigella;s__Escherichia sp. 6B |
| JQMS01000001.5394.6887 | -0.32964 | 0.305556 | I | 12 | 0.0058% | peripheral-nodes | Rare | d__Bacteria;p__Bacteroidetes;c__Flavobacteriia;o__Flavobacteriales;f__Flavobacteriaceae;g__Flavobacterium;s__Flavobacterium sp. 83 |
| EU465136.1.1384 | -0.81856 | 0 | Other | 1 | 0.0058% | peripheral-nodes | Rare | d__Bacteria;p__Proteobacteria;c__Betaproteobacteria;o__Burkholderiales;f__Comamonadaceae;g__Comamonas;s__uncultured bacterium |
| EU558692.1.1407 | -0.22099 | 0.147929 | II | 13 | 0.0059% | peripheral-nodes | Rare | d__Bacteria;p__Proteobacteria;c__Gammaproteobacteria;o__Enterobacteriales;f__Enterobacteriaceae;g__Serratia;s__Serratia sp. ITA-1 |
| AM982567.1.1476 | 0.702519 | 0.065556 | IV | 30 | 0.0059% | peripheral-nodes | Rare | d__Bacteria;p__Firmicutes;c__Clostridia;o__Clostridiales;f__Clostridiaceae 1;g__Clostridium sensu stricto 1;s__uncultured bacterium |
| AM696803.1.1494 | -0.76423 | 0 | II | 2 | 0.0059% | peripheral-nodes | Rare | d__Bacteria;p__Proteobacteria;c__Gammaproteobacteria;o__Enterobacteriales;f__Enterobacteriaceae;g__Serratia;s__uncultured bacterium |
| AM500814.1.1473 | -0.65558 | 0 | VI | 4 | 0.0060% | peripheral-nodes | Rare | d__Bacteria;p__Firmicutes;c__Clostridia;o__Clostridiales;f__Clostridiaceae 1;g__Clostridium sensu stricto 1;s__uncultured bacterium |
| EU774947.1.1392 | -0.16667 | 0.137755 | X | 14 | 0.0060% | peripheral-nodes | Rare | d__Bacteria;p__Proteobacteria;c__Gammaproteobacteria;o__Pseudomonadales;f__Pseudomonadaceae;g__Pseudomonas;s__uncultured bacterium |
| LN564846.1.1296 | -0.54694 | 0.265306 | Other | 7 | 0.0060% | peripheral-nodes | Rare | d__Bacteria;p__Proteobacteria;c__Alphaproteobacteria;o__Rhodobacterales;f__Rhodobacteraceae;g__Rubellimicrobium;s__uncultured bacterium |
| JRXT01000001.116208.117597 | -0.76423 | 0 | Other | 2 | 0.0060% | peripheral-nodes | Rare | d__Bacteria;p__Proteobacteria;c__Gammaproteobacteria;o__Pseudomonadales;f__Pseudomonadaceae;g__Pseudomonas;s__Pseudomonas fluorescens |
| EU775272.1.1284 | 0.376574 | 0.081597 | IV | 24 | 0.0060% | peripheral-nodes | Rare | d__Bacteria;p__Firmicutes;c__Clostridia;o__Clostridiales;f__Clostridiaceae 1;g__Clostridium sensu stricto 1;s__uncultured bacterium |
| EU469571.1.1403 | -0.60126 | 0 | VII | 5 | 0.0060% | peripheral-nodes | Rare | d__Bacteria;p__Proteobacteria;c__Gammaproteobacteria;o__Pseudomonadales;f__Pseudomonadaceae;g__Pseudomonas;s__uncultured bacterium |
| JARP01000001.1246036.1247529 | -2.04669 | 0.99927 | I | 37 | 0.0060% | connectors | Rare | d__Bacteria;p__Bacteroidetes;c__Flavobacteriia;o__Flavobacteriales;f__Flavobacteriaceae;g__Flavobacterium;s__Flavobacterium sp. KJJ |
| LN558591.1.1378 | -0.70991 | 0 | Other | 3 | 0.0061% | peripheral-nodes | Rare | d__Bacteria;p__Proteobacteria;c__Gammaproteobacteria;o__Enterobacteriales;f__Enterobacteriaceae;g__Klebsiella;s__Klebsiella pneumoniae |
| JN049591.1.1506 | 0.104953 | 0.265306 | II | 21 | 0.0061% | peripheral-nodes | Rare | d__Bacteria;p__Proteobacteria;c__Gammaproteobacteria;o__Enterobacteriales;f__Enterobacteriaceae;g__Escherichia-Shigella;s__Escherichia coli |
| JF737926.1.1480 | 0.593871 | 0 | IV | 27 | 0.0061% | peripheral-nodes | Rare | d__Bacteria;p__Firmicutes;c__Clostridia;o__Clostridiales;f__Clostridiaceae 1;g__Clostridium sensu stricto 1;s__uncultured bacterium |
| KU057042.1.1485 | -0.76423 | 0 | II | 2 | 0.0061% | peripheral-nodes | Rare | d__Bacteria;p__Proteobacteria;c__Gammaproteobacteria;o__Enterobacteriales;f__Enterobacteriaceae;g__Serratia;s__Raoultella sp. R47 |
| HM204918.1.1464 | -0.22099 | 0.265306 | III | 14 | 0.0061% | peripheral-nodes | Rare | d__Bacteria;p__Bacteroidetes;c__Sphingobacteriia;o__Sphingobacteriales;f__Sphingobacteriaceae;g__Mucilaginibacter;s__Mucilaginibacter sp. PBI 162 |
| DQ796778.1.1374 | 0.756843 | 0.121094 | IV | 32 | 0.0061% | peripheral-nodes | Rare | d__Bacteria;p__Firmicutes;c__Clostridia;o__Clostridiales;f__Clostridiaceae 1;g__Clostridium sensu stricto 1;s__uncultured bacterium |
| DQ818915.1.1456 | -0.00369 | 0.209877 | II | 18 | 0.0061% | peripheral-nodes | Rare | d__Bacteria;p__Proteobacteria;c__Gammaproteobacteria;o__Enterobacteriales;f__Enterobacteriaceae;g__Escherichia-Shigella;s__uncultured bacterium |
| EF401512.1.1477 | 0.430898 | 0.0784 | IV | 25 | 0.0061% | peripheral-nodes | Rare | d__Bacteria;p__Firmicutes;c__Clostridia;o__Clostridiales;f__Clostridiaceae 1;g__Clostridium sensu stricto 1;s__uncultured bacterium |
| EU460236.1.1396 | -0.38396 | 0 | X | 9 | 0.0061% | peripheral-nodes | Rare | d__Bacteria;p__Proteobacteria;c__Gammaproteobacteria;o__Pseudomonadales;f__Pseudomonadaceae;g__Pseudomonas;s__uncultured bacterium |
| FJ849442.1.1459 | -0.32964 | 0 | X | 10 | 0.0062% | peripheral-nodes | Rare | d__Bacteria;p__Proteobacteria;c__Gammaproteobacteria;o__Pseudomonadales;f__Pseudomonadaceae;g__Pseudomonas;s__uncultured bacterium |
| KC331307.1.1486 | -1.19338 | 1 | Other | 6 | 0.0062% | connectors | Rare | d__Bacteria;p__Proteobacteria;c__Gammaproteobacteria;o__Oceanospirillales;f__Oceanospirillaceae;g__Pseudohongiella;s__uncultured bacterium |
| GBXT01022816.16.1503 | -1.21218 | 1 | II | 5 | 0.0062% | connectors | Rare | d__Bacteria;p__Proteobacteria;c__Gammaproteobacteria;o__Enterobacteriales;f__Enterobacteriaceae;g__Escherichia-Shigella;s__Calanus glacialis |
| EU461221.1.1370 | -0.81856 | 0.75 | Other | 2 | 0.0063% | connectors | Rare | d__Bacteria;p__Firmicutes;c__Clostridia;o__Clostridiales;f__Clostridiaceae 1;g__Sarcina;s__uncultured bacterium |
| AIGT01000040.31355.32884 | -0.76423 | 0 | II | 2 | 0.0063% | peripheral-nodes | Rare | d__Bacteria;p__Proteobacteria;c__Gammaproteobacteria;o__Enterobacteriales;f__Enterobacteriaceae;g__Escherichia-Shigella;s__Escherichia coli DEC10E |
| HQ806988.1.1449 | -0.76423 | 0 | II | 2 | 0.0064% | peripheral-nodes | Rare | d__Bacteria;p__Proteobacteria;c__Gammaproteobacteria;o__Enterobacteriales;f__Enterobacteriaceae;g__Escherichia-Shigella;s__uncultured organism |
| AVCC01000013.58497.60007 | 0.32225 | 0.336077 | III | 27 | 0.0064% | peripheral-nodes | Rare | d__Bacteria;p__Proteobacteria;c__Betaproteobacteria;o__Burkholderiales;f__Oxalobacteraceae;g__Herminiimonas;s__Herminiimonas sp. CN |
| JQ183328.1.1343 | -0.16667 | 0 | IV | 13 | 0.0064% | peripheral-nodes | Rare | d__Bacteria;p__Firmicutes;c__Clostridia;o__Clostridiales;f__Clostridiaceae 1;g__Clostridium sensu stricto 1;s__uncultured bacterium |
| JQ410860.1.1526 | -0.43829 | 0.471074 | II | 11 | 0.0064% | peripheral-nodes | Rare | d__Bacteria;p__Proteobacteria;c__Gammaproteobacteria;o__Enterobacteriales;f__Enterobacteriaceae;g__Serratia;s__uncultured bacterium |
| AWGC01000064.299.1762 | 0.104953 | 0.330579 | III | 22 | 0.0064% | peripheral-nodes | Rare | d__Bacteria;p__Proteobacteria;c__Alphaproteobacteria;o__Caulobacterales;f__Caulobacteraceae;g__Asticcacaulis;s__Asticcacaulis sp. AC402 |
| GQ159555.1.1454 | -0.49261 | 0 | IV | 7 | 0.0064% | peripheral-nodes | Rare | d__Bacteria;p__Firmicutes;c__Clostridia;o__Clostridiales;f__Clostridiaceae 1;g__Clostridium sensu stricto 1;s__uncultured bacterium |
| LVJE01000012.628.2145 | -0.76423 | 0 | I | 2 | 0.0065% | peripheral-nodes | Rare | d__Bacteria;p__Bacteroidetes;c__Flavobacteriia;o__Flavobacteriales;f__Flavobacteriaceae;g__Flavobacterium;s__Flavobacterium fryxellicola |
| AJWX01000059.221.1757 | -0.70991 | 0 | I | 3 | 0.0065% | peripheral-nodes | Rare | d__Bacteria;p__Proteobacteria;c__Gammaproteobacteria;o__Pseudomonadales;f__Pseudomonadaceae;g__Pseudomonas;s__Pseudomonas sp. M47T1 |
| EU538971.1.1386 | 0.756843 | 0.305556 | I | 36 | 0.0065% | peripheral-nodes | Rare | d__Bacteria;p__Bacteroidetes;c__Flavobacteriia;o__Flavobacteriales;f__Flavobacteriaceae;g__Flavobacterium;s__uncultured bacterium |
| GQ222391.1.1427 | -0.05802 | 0.221453 | II | 17 | 0.0065% | peripheral-nodes | Rare | d__Bacteria;p__Proteobacteria;c__Gammaproteobacteria;o__Enterobacteriales;f__Enterobacteriaceae;g__Escherichia-Shigella;s__Escherichia coli |
| JF712664.1.1532 | -0.76423 | 0 | Other | 2 | 0.0066% | peripheral-nodes | Rare | d__Bacteria;p__Proteobacteria;c__Gammaproteobacteria;o__Enterobacteriales;f__Enterobacteriaceae;g__Escherichia-Shigella;s__uncultured gamma proteobacterium |
| ARBN01000051.64.1564 | 0.756843 | 0.221453 | III | 34 | 0.0066% | peripheral-nodes | Rare | d__Bacteria;p__Bacteroidetes;c__Cytophagia;o__Cytophagales;f__Cytophagaceae;g__Cytophaga;s__Cytophaga aurantiaca DSM 3654 |
| HQ407221.1.1411 | -0.70991 | 0 | II | 3 | 0.0066% | peripheral-nodes | Rare | d__Bacteria;p__Proteobacteria;c__Gammaproteobacteria;o__Enterobacteriales;f__Enterobacteriaceae;g__Escherichia-Shigella;s__Escherichia fergusonii |
| KJ361504.1.1434 | -0.65555 | 1 | VI | 1 | 0.0066% | connectors | Rare | d__Bacteria;p__Proteobacteria;c__Betaproteobacteria;o__Burkholderiales;f__Oxalobacteraceae;g__Massilia;s__Massilia eurypsychrophila |
| KC539477.1.1396 | -0.38396 | 0.330579 | II | 11 | 0.0067% | peripheral-nodes | Rare | d__Bacteria;p__Proteobacteria;c__Gammaproteobacteria;o__Enterobacteriales;f__Enterobacteriaceae;g__Escherichia-Shigella;s__Escherichia coli |
| EU774638.1.1387 | 0.485222 | 0 | IV | 25 | 0.0067% | peripheral-nodes | Rare | d__Bacteria;p__Firmicutes;c__Clostridia;o__Clostridiales;f__Clostridiaceae 1;g__Clostridium sensu stricto 1;s__uncultured bacterium |
| FLKB01000005.3297.4792 | -0.54694 | 0 | Other | 6 | 0.0067% | peripheral-nodes | Rare | d__Bacteria;p__Firmicutes;c__Clostridia;o__Clostridiales;f__Clostridiaceae 1;g__Clostridium sensu stricto 1;s__Clostridium sp. Marseille-P2414 |
| LK392803.1.1398 | -0.81856 | 0 | VI | 1 | 0.0067% | peripheral-nodes | Rare | d__Bacteria;p__Proteobacteria;c__Betaproteobacteria;o__Burkholderiales;f__Comamonadaceae;g__Aquabacterium;s__uncultured bacterium |
| AJ242969.1.1539 | -0.11234 | 0 | VIII | 14 | 0.0067% | peripheral-nodes | Rare | d__Bacteria;p__Firmicutes;c__Bacilli;o__Lactobacillales;f__Lactobacillaceae;g__Lactobacillus;s__Lactobacillus crispatus |
| JF198605.1.1334 | -0.81856 | 0 | V | 1 | 0.0068% | peripheral-nodes | Rare | d__Bacteria;p__Firmicutes;c__Clostridia;o__Clostridiales;f__Clostridiaceae 1;g__Clostridium sensu stricto 1;s__uncultured bacterium |
| LMKO01000022.441673.443213 | -0.65555 | 1 | VI | 3 | 0.0068% | connectors | Rare | d__Bacteria;p__Proteobacteria;c__Betaproteobacteria;o__Burkholderiales;f__Comamonadaceae;g__Xylophilus;s__Xylophilus sp. Leaf220 |
| HQ739279.1.1450 | 0.32225 | 0.2256 | II | 25 | 0.0068% | peripheral-nodes | Rare | d__Bacteria;p__Proteobacteria;c__Gammaproteobacteria;o__Enterobacteriales;f__Enterobacteriaceae;g__Escherichia-Shigella;s__uncultured bacterium |
| FJ906919.1.1457 | -0.70991 | 0 | II | 3 | 0.0068% | peripheral-nodes | Rare | d__Bacteria;p__Proteobacteria;c__Gammaproteobacteria;o__Enterobacteriales;f__Enterobacteriaceae;g__Cronobacter;s__Cronobacter sakazakii |
| JOEB01000011.3693.5194 | -0.76423 | 0.555556 | I | 3 | 0.0069% | peripheral-nodes | Rare | d__Bacteria;p__Actinobacteria;c__Actinobacteria;o__Micrococcales;f__Microbacteriaceae;g__Mycetocola;s__Salana multivorans |
| JX184912.1.1462 | -0.22099 | 0.36 | VII | 15 | 0.0069% | peripheral-nodes | Rare | d__Bacteria;p__Proteobacteria;c__Gammaproteobacteria;o__Pseudomonadales;f__Pseudomonadaceae;g__Pseudomonas;s__Pseudomonas fragi A22 |
| LN561623.1.1318 | -0.87288 | 1 | Other | 1 | 0.0069% | connectors | Rare | d__Bacteria;p__Proteobacteria;c__Alphaproteobacteria;o__Rhizobiales;f__Hyphomicrobiaceae;g__Pelagibacterium;s__uncultured bacterium |
| AQER01000022.44207.45735 | -0.43829 | 0.209877 | IX | 9 | 0.0070% | peripheral-nodes | Rare | d__Bacteria;p__Proteobacteria;c__Gammaproteobacteria;o__Enterobacteriales;f__Enterobacteriaceae;g__Escherichia-Shigella;s__Escherichia coli P0299917.1 |
| GQ132642.1.1378 | -0.54694 | 0.4375 | V | 8 | 0.0070% | peripheral-nodes | Rare | d__Bacteria;p__Firmicutes;c__Clostridia;o__Clostridiales;f__Clostridiaceae 1;g__Clostridium sensu stricto 1;s__uncultured bacterium |
| KJ808419.1.1480 | 0.648195 | 0.128889 | IV | 30 | 0.0070% | peripheral-nodes | Rare | d__Bacteria;p__Firmicutes;c__Clostridia;o__Clostridiales;f__Clostridiaceae 1;g__Clostridium sensu stricto 1;s__uncultured bacterium |
| BCRT01000028.282.1785 | -0.81856 | 0 | Other | 1 | 0.0070% | peripheral-nodes | Rare | d__Bacteria;p__Actinobacteria;c__Actinobacteria;o__Micrococcales;f__Sanguibacteraceae;g__Sanguibacter;s__Sanguibacter suarezii NBRC 16159 |
| AY958779.1.1487 | 0.32225 | 0.159722 | I | 24 | 0.0070% | peripheral-nodes | Rare | d__Bacteria;p__Proteobacteria;c__Gammaproteobacteria;o__Pseudomonadales;f__Pseudomonadaceae;g__Pseudomonas;s__uncultured bacterium |
| EU459276.1.1307 | 0.267926 | 0 | IV | 21 | 0.0070% | peripheral-nodes | Rare | d__Bacteria;p__Firmicutes;c__Clostridia;o__Clostridiales;f__Clostridiaceae 1;g__Clostridium sensu stricto 1;s__uncultured bacterium |
| HQ407269.1.1521 | -0.76423 | 0 | I | 2 | 0.0071% | peripheral-nodes | Rare | d__Bacteria;p__Proteobacteria;c__Gammaproteobacteria;o__Xanthomonadales;f__Xanthomonadaceae;g__Wohlfahrtiimonas;s__Wohlfahrtiimonas chitiniclastica |
| KF150657.1.1454 | 0.104953 | 0.265306 | I | 21 | 0.0071% | peripheral-nodes | Rare | d__Bacteria;p__Proteobacteria;c__Gammaproteobacteria;o__Pseudomonadales;f__Pseudomonadaceae;g__Pseudomonas;s__Pseudomonas sp. DW-1 |
| CQAM01000080.14904.16434 | -0.16667 | 0.248889 | II | 15 | 0.0072% | peripheral-nodes | Rare | d__Bacteria;p__Proteobacteria;c__Gammaproteobacteria;o__Enterobacteriales;f__Enterobacteriaceae;g__Yersinia;s__Yersinia enterocolitica subsp. enterocolitica |
| KU057056.1.1484 | -0.00369 | 0.209877 | II | 18 | 0.0072% | peripheral-nodes | Rare | d__Bacteria;p__Proteobacteria;c__Gammaproteobacteria;o__Enterobacteriales;f__Enterobacteriaceae;g__Serratia;s__Serratia sp. H62 |
| FQ658871.1.1363 | -0.43829 | 0 | II | 8 | 0.0072% | peripheral-nodes | Rare | d__Bacteria;p__Proteobacteria;c__Gammaproteobacteria;o__Enterobacteriales;f__Enterobacteriaceae;g__Escherichia-Shigella;s__uncultured soil bacterium |
| EU473256.1.1382 | 0.919815 | 0.159722 | IV | 36 | 0.0072% | peripheral-nodes | Rare | d__Bacteria;p__Firmicutes;c__Clostridia;o__Clostridiales;f__Clostridiaceae 1;g__Clostridium sensu stricto 1;s__uncultured bacterium |
| LXUF01000018.277.1823 | 1.789002 | 0.261003 | III | 57 | 0.0073% | peripheral-nodes | Rare | d__Bacteria;p__Proteobacteria;c__Betaproteobacteria;o__Methylophilales;f__Methylophilaceae;g__uncultured;s__Methylovorus sp. MM1 |
| AB128892.1.1504 | -0.76423 | 0 | VI | 2 | 0.0073% | peripheral-nodes | Rare | d__Bacteria;p__Proteobacteria;c__Betaproteobacteria;o__Burkholderiales;f__Comamonadaceae;g__Aquabacterium;s__uncultured bacterium |
| AB078842.1.1447 | -0.87288 | 1 | Other | 1 | 0.0073% | connectors | Rare | d__Bacteria;p__Bacteroidetes;c__Bacteroidia;o__Bacteroidales;f__Porphyromonadaceae;g__Paludibacter;s__Paludibacter propionicigenes |
| ANVS01000015.616708.618034 | 0.485222 | 0.202806 | II | 28 | 0.0074% | peripheral-nodes | Rare | d__Bacteria;p__Proteobacteria;c__Gammaproteobacteria;o__Enterobacteriales;f__Enterobacteriaceae;g__Escherichia-Shigella;s__Escherichia coli KTE46 |
| HQ674944.1.1469 | -0.54694 | 0 | V | 6 | 0.0074% | peripheral-nodes | Rare | d__Bacteria;p__Acidobacteria;c__Acidobacteria;o__Acidobacteriales;f__Acidobacteriaceae (Subgroup 1);g__Granulicella;s__uncultured Acidobacteria bacterium |
| GQ267975.1.1475 | -0.27532 | 0 | X | 11 | 0.0074% | peripheral-nodes | Rare | d__Bacteria;p__Proteobacteria;c__Gammaproteobacteria;o__Pseudomonadales;f__Pseudomonadaceae;g__Pseudomonas;s__uncultured bacterium |
| AJ289161.1.1484 | 0.919815 | 0.245845 | III | 38 | 0.0075% | peripheral-nodes | Rare | d__Bacteria;p__Proteobacteria;c__Gammaproteobacteria;o__Cellvibrionales;f__Cellvibrionaceae;g__Cellvibrio;s__Cellvibrio sp. R-4001 |
| AB021339.1.1446 | -0.81856 | 0 | Other | 1 | 0.0077% | peripheral-nodes | Rare | d__Bacteria;p__Proteobacteria;c__Betaproteobacteria;o__Burkholderiales;f__Comamonadaceae;g__Diaphorobacter;s__bacterium rM4 |
| LC140804.1.1507 | -0.81856 | 0 | II | 1 | 0.0077% | peripheral-nodes | Rare | d__Bacteria;p__Proteobacteria;c__Gammaproteobacteria;o__Enterobacteriales;f__Enterobacteriaceae;g__Serratia;s__uncultured Serratia sp. |
| GBHB01070766.2.1503 | -0.05802 | 0.121094 | I | 16 | 0.0077% | peripheral-nodes | Rare | d__Bacteria;p__Proteobacteria;c__Gammaproteobacteria;o__Pseudomonadales;f__Pseudomonadaceae;g__Pseudomonas;s__Teleogryllus commodus |
| JUEH01000050.3451.4829 | -0.87288 | 1 | Other | 1 | 0.0078% | connectors | Rare | d__Bacteria;p__Proteobacteria;c__Gammaproteobacteria;o__Pseudomonadales;f__Pseudomonadaceae;g__Pseudomonas;s__Pseudomonas nitroreducens |
| AY315175.1.1393 | -0.76423 | 0.555556 | III | 3 | 0.0078% | peripheral-nodes | Rare | d__Bacteria;p__Proteobacteria;c__Betaproteobacteria;o__Burkholderiales;f__Comamonadaceae;g__Polaromonas;s__glacier bacterium FJS17 |
| EU460309.1.1298 | -0.32964 | 0.173554 | VII | 11 | 0.0078% | peripheral-nodes | Rare | d__Bacteria;p__Proteobacteria;c__Gammaproteobacteria;o__Pseudomonadales;f__Pseudomonadaceae;g__Pseudomonas;s__uncultured bacterium |
| JQ410828.1.1530 | -0.32964 | 0 | II | 10 | 0.0079% | peripheral-nodes | Rare | d__Bacteria;p__Proteobacteria;c__Gammaproteobacteria;o__Enterobacteriales;f__Enterobacteriaceae;g__Serratia;s__uncultured bacterium |
| AYSU01000028.26826.28292 | -0.65555 | 1 | V | 2 | 0.0080% | connectors | Rare | d__Bacteria;p__Proteobacteria;c__Alphaproteobacteria;o__Rhizobiales;f__Bradyrhizobiaceae;g__Rhodopseudomonas;s__Rhodopseudomonas palustris JSC-3b |
| JF234721.1.1335 | -0.43829 | 0.471074 | V | 11 | 0.0080% | peripheral-nodes | Rare | d__Bacteria;p__Firmicutes;c__Clostridia;o__Clostridiales;f__Clostridiaceae 1;g__Clostridium sensu stricto 1;s__uncultured bacterium |
| DQ815993.1.1428 | -0.38396 | 0.330579 | V | 11 | 0.0080% | peripheral-nodes | Rare | d__Bacteria;p__Firmicutes;c__Clostridia;o__Clostridiales;f__Clostridiaceae 1;g__Clostridium sensu stricto 1;s__uncultured bacterium |
| AY468453.1.1450 | 2.114946 | 0.187046 | I | 61 | 0.0081% | peripheral-nodes | Rare | d__Bacteria;p__Bacteroidetes;c__Flavobacteriia;o__Flavobacteriales;f__Flavobacteriaceae;g__Flavobacterium;s__cf. Chryseobacterium sp. UOF CM895 |
| KF080806.1.1364 | 0.104953 | 0.19 | II | 20 | 0.0081% | peripheral-nodes | Rare | d__Bacteria;p__Proteobacteria;c__Gammaproteobacteria;o__Enterobacteriales;f__Enterobacteriaceae;g__Serratia;s__uncultured bacterium |
| JRLX01000051.261.1786 | 1.517381 | 0.2256 | I | 50 | 0.0082% | peripheral-nodes | Rare | d__Bacteria;p__Bacteroidetes;c__Flavobacteriia;o__Flavobacteriales;f__Flavobacteriaceae;g__Flavobacterium;s__Flavobacterium rivuli WB 3.3-2 = DSM 21788 |
| EF608528.1.1461 | -0.05802 | 0.305556 | II | 18 | 0.0082% | peripheral-nodes | Rare | d__Bacteria;p__Proteobacteria;c__Gammaproteobacteria;o__Enterobacteriales;f__Enterobacteriaceae;g__norank;s__uncultured bacterium |
| HQ807522.1.1456 | -0.76423 | 0 | II | 2 | 0.0082% | peripheral-nodes | Rare | d__Bacteria;p__Proteobacteria;c__Gammaproteobacteria;o__Enterobacteriales;f__Enterobacteriaceae;g__Escherichia-Shigella;s__uncultured organism |
| EU774989.1.1390 | -0.00369 | 0 | X | 16 | 0.0083% | peripheral-nodes | Rare | d__Bacteria;p__Proteobacteria;c__Gammaproteobacteria;o__Pseudomonadales;f__Pseudomonadaceae;g__Pseudomonas;s__uncultured bacterium |
| GQ898504.1.1475 | 0.756843 | 0.063476 | IV | 31 | 0.0083% | peripheral-nodes | Rare | d__Bacteria;p__Firmicutes;c__Clostridia;o__Clostridiales;f__Clostridiaceae 1;g__Clostridium sensu stricto 1;s__uncultured bacterium |
| APWT01000006.129743.131262 | -0.60126 | 0 | Other | 5 | 0.0083% | peripheral-nodes | Rare | d__Bacteria;p__Proteobacteria;c__Gammaproteobacteria;o__Pseudomonadales;f__Pseudomonadaceae;g__Pseudomonas;s__Pseudomonas syringae pv. syringae SM |
| KP745593.1.1439 | -0.43829 | 0 | I | 8 | 0.0083% | peripheral-nodes | Rare | d__Bacteria;p__Proteobacteria;c__Gammaproteobacteria;o__Pseudomonadales;f__Pseudomonadaceae;g__Pseudomonas;s__Pseudomonas fragi |
| GQ267972.1.1461 | 0.756843 | 0.063476 | I | 31 | 0.0084% | peripheral-nodes | Rare | d__Bacteria;p__Proteobacteria;c__Gammaproteobacteria;o__Pseudomonadales;f__Pseudomonadaceae;g__Pseudomonas;s__uncultured bacterium |
| AB681873.1.1467 | 0.376574 | 0.217456 | II | 26 | 0.0084% | peripheral-nodes | Rare | d__Bacteria;p__Proteobacteria;c__Gammaproteobacteria;o__Enterobacteriales;f__Enterobacteriaceae;g__Serratia;s__Serratia ficaria |
| LC055581.1.1501 | -0.65555 | 1 | VII | 20 | 0.0084% | connectors | Rare | d__Bacteria;p__Proteobacteria;c__Gammaproteobacteria;o__Pseudomonadales;f__Pseudomonadaceae;g__Pseudomonas;s__uncultured Pseudomonas sp. |
| HQ176032.1.1473 | 0.430898 | 0.0784 | IV | 25 | 0.0085% | peripheral-nodes | Rare | d__Bacteria;p__Firmicutes;c__Clostridia;o__Clostridiales;f__Clostridiaceae 1;g__Clostridium sensu stricto 1;s__uncultured bacterium |
| HM270587.1.1373 | 3.038457 | 0.247496 | III | 83 | 0.0085% | module-hubs | Rare | d__Bacteria;p__Proteobacteria;c__Deltaproteobacteria;o__Bdellovibrionales;f__Bacteriovoracaceae;g__Bacteriovorax;s__uncultured bacterium |
| KF109672.1.1363 | 0.756843 | 0.173554 | II | 33 | 0.0086% | peripheral-nodes | Rare | d__Bacteria;p__Proteobacteria;c__Gammaproteobacteria;o__Enterobacteriales;f__Enterobacteriaceae;g__Serratia;s__uncultured bacterium |
| EU451330.1.1432 | -0.11234 | 0 | VIII | 14 | 0.0086% | peripheral-nodes | Rare | d__Bacteria;p__Firmicutes;c__Bacilli;o__Lactobacillales;f__Lactobacillaceae;g__Lactobacillus;s__uncultured bacterium |
| AB680072.1.1467 | 0.648195 | 0.184183 | II | 31 | 0.0087% | peripheral-nodes | Rare | d__Bacteria;p__Proteobacteria;c__Gammaproteobacteria;o__Enterobacteriales;f__Enterobacteriaceae;g__Escherichia-Shigella;s__Escherichia coli |
| JQ815715.1.1485 | 0.376574 | 0.081597 | IV | 24 | 0.0087% | peripheral-nodes | Rare | d__Bacteria;p__Firmicutes;c__Clostridia;o__Clostridiales;f__Clostridiaceae 1;g__Clostridium sensu stricto 1;s__uncultured bacterium |
| HQ176208.1.1431 | -0.70991 | 0.4375 | Other | 4 | 0.0088% | peripheral-nodes | Rare | d__Bacteria;p__Firmicutes;c__Clostridia;o__Clostridiales;f__Clostridiaceae 1;g__Clostridium sensu stricto 1;s__uncultured bacterium |
| KF842941.1.1417 | -0.56549 | 0.99762 | II | 41 | 0.0088% | connectors | Rare | d__Bacteria;p__Proteobacteria;c__Gammaproteobacteria;o__Enterobacteriales;f__Enterobacteriaceae;g__Escherichia-Shigella;s__uncultured bacterium |
| KM035944.1.1434 | 2.060622 | 0.241415 | I | 62 | 0.0088% | peripheral-nodes | Rare | d__Bacteria;p__Bacteroidetes;c__Sphingobacteriia;o__Sphingobacteriales;f__Sphingobacteriaceae;g__Pedobacter;s__Pedobacter trunci |
| GQ222390.1.1427 | 0.050629 | 0.2775 | II | 20 | 0.0089% | peripheral-nodes | Rare | d__Bacteria;p__Proteobacteria;c__Gammaproteobacteria;o__Enterobacteriales;f__Enterobacteriaceae;g__Escherichia-Shigella;s__Escherichia coli |
| EU469572.1.1395 | 0.97414 | 0.155588 | I | 37 | 0.0089% | peripheral-nodes | Rare | d__Bacteria;p__Proteobacteria;c__Gammaproteobacteria;o__Pseudomonadales;f__Pseudomonadaceae;g__Pseudomonas;s__uncultured bacterium |
| AB491200.1.1443 | 1.408733 | 0.128889 | II | 45 | 0.0089% | peripheral-nodes | Rare | d__Bacteria;p__Proteobacteria;c__Gammaproteobacteria;o__Enterobacteriales;f__Enterobacteriaceae;g__Cedecea;s__Enterobacterial endosymbiont of Drosicha corpulenta |
| KT369683.1.1408 | -0.70991 | 0 | I | 3 | 0.0090% | peripheral-nodes | Rare | d__Bacteria;p__Proteobacteria;c__Gammaproteobacteria;o__Pseudomonadales;f__Pseudomonadaceae;g__Pseudomonas;s__bacterium ASP2 |
| FJ444768.1.1511 | 0.32225 | 0.284024 | III | 26 | 0.0090% | peripheral-nodes | Rare | d__Bacteria;p__Verrucomicrobia;c__Opitutae;o__Opitutales;f__Opitutaceae;g__Opitutus;s__uncultured bacterium |
| AY342004.1.1500 | -0.81856 | 0 | I | 1 | 0.0091% | peripheral-nodes | Rare | d__Bacteria;p__Proteobacteria;c__Gammaproteobacteria;o__Pseudomonadales;f__Pseudomonadaceae;g__Pseudomonas;s__Pseudomonas sp. CA18 |
| LFBR01000002.190330.192178 | 1.191436 | 0.140988 | II | 41 | 0.0091% | peripheral-nodes | Rare | d__Bacteria;p__Proteobacteria;c__Gammaproteobacteria;o__Enterobacteriales;f__Enterobacteriaceae;g__Serratia;s__Serratia marcescens |
| GQ383921.1.1450 | -1.01036 | 1 | Other | 1 | 0.0091% | connectors | Rare | d__Bacteria;p__Proteobacteria;c__Alphaproteobacteria;o__Rhizobiales;f__Hyphomicrobiaceae;g__Pelagibacterium;s__Hyphomicrobiaceae bacterium H642 |
| JQ746036.1.1542 | -0.81856 | 0 | Other | 1 | 0.0092% | peripheral-nodes | Rare | d__Bacteria;p__Proteobacteria;c__Gammaproteobacteria;o__Xanthomonadales;f__Xanthomonadaceae;g__Vulcaniibacterium;s__Vulcaniibacterium thermophilum |
| GDHW01147822.1.1369 | -0.8765 | 1 | I | 1 | 0.0092% | connectors | Rare | d__Bacteria;p__Proteobacteria;c__Betaproteobacteria;o__Burkholderiales;f__Comamonadaceae;g__Sphaerotilus;s__Austrofundulus limnaeus |
| BAME01000208.20.1360 | -0.76423 | 0 | Other | 2 | 0.0092% | peripheral-nodes | Rare | d__Bacteria;p__Bacteroidetes;c__Bacteroidia;o__Bacteroidales;f__Porphyromonadaceae;g__Porphyromonas;s__Porphyromonas bennonis DSM 23058 = JCM 16335 |
| GBYS01010499.306.1788 | -0.81856 | 0 | Other | 1 | 0.0093% | peripheral-nodes | Rare | d__Bacteria;p__Cyanobacteria;c__Chloroplast;o__norank;f__norank;g__norank;s__Oryza meyeriana |
| JN867385.1.1504 | -0.70991 | 0 | Other | 3 | 0.0093% | peripheral-nodes | Rare | d__Bacteria;p__Proteobacteria;c__Gammaproteobacteria;o__Pseudomonadales;f__Pseudomonadaceae;g__Pseudomonas;s__uncultured bacterium |
| JPLY01000001.145690.147219 | -0.00369 | 0.36 | I | 20 | 0.0093% | peripheral-nodes | Rare | d__Bacteria;p__Bacteroidetes;c__Flavobacteriia;o__Flavobacteriales;f__Flavobacteriaceae;g__Epilithonimonas;s__Epilithonimonas lactis |
| LECU01000006.90.1617 | 1.300084 | 0.27569 | I | 47 | 0.0094% | peripheral-nodes | Rare | d__Bacteria;p__Bacteroidetes;c__Sphingobacteriia;o__Sphingobacteriales;f__Sphingobacteriaceae;g__Pedobacter;s__Pedobacter sp. BMA |
| KC189756.1.1450 | 0.159278 | 0.31758 | III | 23 | 0.0094% | peripheral-nodes | Rare | d__Bacteria;p__Proteobacteria;c__Alphaproteobacteria;o__Alphaproteobacteria Incertae Sedis;f__uncultured;g__norank;s__uncultured bacterium |
| KF065335.1.1373 | -0.81856 | 0 | Other | 1 | 0.0095% | peripheral-nodes | Rare | d__Bacteria;p__Firmicutes;c__Bacilli;o__Lactobacillales;f__Leuconostocaceae;g__Leuconostoc;s__uncultured bacterium |
| M58820.1.1521 | -0.11234 | 0.128889 | VIII | 15 | 0.0095% | peripheral-nodes | Rare | d__Bacteria;p__Firmicutes;c__Bacilli;o__Lactobacillales;f__Lactobacillaceae;g__Lactobacillus;s__Lactobacillus gasseri |
| FCOY01000002.274549.276073 | -0.81856 | 0 | Other | 1 | 0.0095% | peripheral-nodes | Rare | d__Bacteria;p__Proteobacteria;c__Gammaproteobacteria;o__Xanthomonadales;f__Xanthomonadaceae;g__Xylella;s__Xanthomonas sp. SN8 |
| HM459688.1.1456 | -0.70991 | 0.4375 | III | 4 | 0.0095% | peripheral-nodes | Rare | d__Bacteria;p__Verrucomicrobia;c__Opitutae;o__Opitutae vadinHA64;f__norank;g__norank;s__uncultured bacterium |
| JF901345.1.1423 | -0.22099 | 0.147929 | II | 13 | 0.0096% | peripheral-nodes | Rare | d__Bacteria;p__Proteobacteria;c__Gammaproteobacteria;o__Enterobacteriales;f__Enterobacteriaceae;g__Rahnella;s__endophytic bacterium 90L-3 |
| KR019684.1.1490 | 1.082788 | 0.147929 | II | 39 | 0.0096% | peripheral-nodes | Rare | d__Bacteria;p__Proteobacteria;c__Gammaproteobacteria;o__Enterobacteriales;f__Enterobacteriaceae;g__Serratia;s__Serratia odorifera |
| KF828863.1.1384 | -0.76423 | 0 | Other | 2 | 0.0096% | peripheral-nodes | Rare | d__Bacteria;p__Proteobacteria;c__Gammaproteobacteria;o__Enterobacteriales;f__Enterobacteriaceae;g__Pantoea;s__Pantoea sp. XJJC-134-5RF4 |
| AE014075.4699063.4700613 | -0.27532 | 0 | II | 11 | 0.0097% | peripheral-nodes | Rare | d__Bacteria;p__Proteobacteria;c__Gammaproteobacteria;o__Enterobacteriales;f__Enterobacteriaceae;g__Escherichia-Shigella;s__Escherichia coli CFT073 |
| FJ868840.1.1463 | -0.11234 | 0.234375 | VII | 16 | 0.0097% | peripheral-nodes | Rare | d__Bacteria;p__Proteobacteria;c__Gammaproteobacteria;o__Pseudomonadales;f__Pseudomonadaceae;g__Pseudomonas;s__uncultured bacterium |
| EU730914.1.1361 | -0.65555 | 1 | I | 6 | 0.0097% | connectors | Rare | d__Bacteria;p__Proteobacteria;c__Alphaproteobacteria;o__Caulobacterales;f__Caulobacteraceae;g__Brevundimonas;s__Brevundimonas diminuta |
| CBVQ010000169.16978.18487 | -0.43829 | 0.471074 | III | 11 | 0.0098% | peripheral-nodes | Rare | d__Bacteria;p__Actinobacteria;c__Actinobacteria;o__Micrococcales;f__Microbacteriaceae;g__Microbacterium;s__Microbacterium sp. C448 |
| EF111107.1.1287 | -0.70991 | 0 | III | 3 | 0.0098% | peripheral-nodes | Rare | d__Bacteria;p__Proteobacteria;c__Gammaproteobacteria;o__Pseudomonadales;f__Pseudomonadaceae;g__Pseudomonas;s__Pseudomonas gingeri |
| GQ018414.1.1345 | -0.81856 | 0 | Other | 1 | 0.0098% | peripheral-nodes | Rare | d__Bacteria;p__Bacteroidetes;c__Flavobacteriia;o__Flavobacteriales;f__Flavobacteriaceae;g__Chryseobacterium;s__uncultured bacterium |
| CP014504.5065363.5066872 | -0.32964 | 0.305556 | I | 12 | 0.0099% | peripheral-nodes | Rare | d__Bacteria;p__Bacteroidetes;c__Sphingobacteriia;o__Sphingobacteriales;f__Sphingobacteriaceae;g__Pedobacter;s__Pedobacter cryoconitis |
| LAZX01000092.189166.190655 | 0.811167 | 0.258488 | I | 36 | 0.0100% | peripheral-nodes | Moderate | d__Bacteria;p__Proteobacteria;c__Alphaproteobacteria;o__Sphingomonadales;f__Sphingomonadaceae;g__Sphingomonas;s__Sphingomonas sp. Ag1 |
| GQ383925.1.1483 | -0.54694 | 0 | I | 6 | 0.0101% | peripheral-nodes | Moderate | d__Bacteria;p__Bacteroidetes;c__Flavobacteriia;o__Flavobacteriales;f__Flavobacteriaceae;g__Empedobacter;s__Algoriella xinjiangensis |
| EU474097.1.1381 | -0.54694 | 0.4375 | V | 8 | 0.0101% | peripheral-nodes | Moderate | d__Bacteria;p__Firmicutes;c__Clostridia;o__Clostridiales;f__Clostridiaceae 1;g__Clostridium sensu stricto 1;s__uncultured bacterium |
| LC125154.1.1442 | -0.76423 | 0 | IV | 2 | 0.0101% | peripheral-nodes | Moderate | d__Bacteria;p__Proteobacteria;c__Gammaproteobacteria;o__Pseudomonadales;f__Pseudomonadaceae;g__Pseudomonas;s__Pseudomonas sp. Oh-1.0.2 |
| CQ955777.4.1525 | 0.865491 | 0.164082 | II | 35 | 0.0102% | peripheral-nodes | Moderate | d__Bacteria;p__Proteobacteria;c__Gammaproteobacteria;o__Enterobacteriales;f__Enterobacteriaceae;g__Serratia;s__unidentified |
| CXWL01003141.114212.115751 | -0.87288 | 1 | Other | 1 | 0.0102% | connectors | Moderate | d__Bacteria;p__Verrucomicrobia;c__Opitutae;o__Opitutales;f__Opitutaceae;g__Opitutus;s__groundwater metagenome |
| AQEN01000039.658693.660217 | 0.702519 | 0.178711 | II | 32 | 0.0103% | peripheral-nodes | Moderate | d__Bacteria;p__Proteobacteria;c__Gammaproteobacteria;o__Enterobacteriales;f__Enterobacteriaceae;g__Escherichia-Shigella;s__Escherichia coli P0304777.1 |
| KF465374.1.1405 | 0.539546 | 0 | I | 26 | 0.0103% | peripheral-nodes | Moderate | d__Bacteria;p__Proteobacteria;c__Gammaproteobacteria;o__Pseudomonadales;f__Pseudomonadaceae;g__Pseudomonas;s__uncultured bacterium |
| JX145037.1.1531 | 1.408733 | 0.128889 | II | 45 | 0.0103% | peripheral-nodes | Moderate | d__Bacteria;p__Proteobacteria;c__Gammaproteobacteria;o__Enterobacteriales;f__Enterobacteriaceae;g__Escherichia-Shigella;s__gamma proteobacterium POB1 |
| CP009278.101598.103127 | -0.81856 | 0 | I | 1 | 0.0104% | peripheral-nodes | Moderate | d__Bacteria;p__Bacteroidetes;c__Sphingobacteriia;o__Sphingobacteriales;f__Sphingobacteriaceae;g__Sphingobacterium;s__Sphingobacterium sp. ML3W |
| JROH01000090.372.1843 | 1.789002 | 0.261003 | III | 57 | 0.0104% | peripheral-nodes | Moderate | d__Bacteria;p__Proteobacteria;c__Alphaproteobacteria;o__Sphingomonadales;f__Sphingomonadaceae;g__Sphingomonas;s__Sphingomonas sp. 37zxx |
| BATC01000012.9360.10830 | -0.87288 | 1 | Other | 1 | 0.0104% | connectors | Moderate | d__Bacteria;p__Proteobacteria;c__Alphaproteobacteria;o__Caulobacterales;f__Caulobacteraceae;g__Brevundimonas;s__Brevundimonas abyssalis TAR-001 |
| LJOJ01000050.3.1288 | 1.517381 | 0.123585 | II | 47 | 0.0105% | peripheral-nodes | Moderate | d__Bacteria;p__Proteobacteria;c__Gammaproteobacteria;o__Enterobacteriales;f__Enterobacteriaceae;g__Escherichia-Shigella;s__Escherichia coli |
| EU473153.1.1384 | -0.81856 | 0 | V | 1 | 0.0105% | peripheral-nodes | Moderate | d__Bacteria;p__Firmicutes;c__Clostridia;o__Clostridiales;f__Clostridiaceae 1;g__Clostridium sensu stricto 1;s__uncultured bacterium |
| JF179845.1.1357 | -0.8765 | 1 | VII | 23 | 0.0105% | connectors | Moderate | d__Bacteria;p__Proteobacteria;c__Gammaproteobacteria;o__Pseudomonadales;f__Pseudomonadaceae;g__Pseudomonas;s__uncultured bacterium |
| GQ246673.1.1422 | -0.81856 | 0 | I | 1 | 0.0106% | peripheral-nodes | Moderate | d__Bacteria;p__Bacteroidetes;c__Flavobacteriia;o__Flavobacteriales;f__Flavobacteriaceae;g__norank;s__Wautersiella sp. M1T8B12 |
| HM038005.1.1456 | -0.49261 | 0.234375 | III | 8 | 0.0106% | peripheral-nodes | Moderate | d__Bacteria;p__Proteobacteria;c__Betaproteobacteria;o__Burkholderiales;f__Oxalobacteraceae;g__Rugamonas;s__Rugamonas rubra |
| EU469621.1.1396 | 1.24576 | 0.095181 | I | 41 | 0.0107% | peripheral-nodes | Moderate | d__Bacteria;p__Proteobacteria;c__Gammaproteobacteria;o__Pseudomonadales;f__Pseudomonadaceae;g__Pseudomonas;s__uncultured bacterium |
| AUGO01000020.105.1600 | -0.76423 | 0.555556 | I | 3 | 0.0107% | peripheral-nodes | Moderate | d__Bacteria;p__Bacteroidetes;c__Flavobacteriia;o__Flavobacteriales;f__Flavobacteriaceae;g__Flavobacterium;s__Flavobacterium soli DSM 19725 |
| FN824879.1.1231 | 0.430898 | 0.265306 | III | 28 | 0.0108% | peripheral-nodes | Moderate | d__Bacteria;p__Proteobacteria;c__Alphaproteobacteria;o__Alphaproteobacteria Incertae Sedis;f__uncultured;g__norank;s__uncultured bacterium |
| JRUQ01000100.9.1559 | -0.70991 | 0 | Other | 3 | 0.0108% | peripheral-nodes | Moderate | d__Bacteria;p__Proteobacteria;c__Gammaproteobacteria;o__Enterobacteriales;f__Enterobacteriaceae;g__Erwinia;s__Erwinia typographi |
| JN637320.1.1396 | 0.702519 | 0.313469 | I | 35 | 0.0109% | peripheral-nodes | Moderate | d__Bacteria;p__Bacteroidetes;c__Sphingobacteriia;o__Sphingobacteriales;f__Sphingobacteriaceae;g__Pedobacter;s__Pedobacter sp. hp16 |
| JUHE01000001.1123234.1124752 | 0.811167 | 0.298028 | I | 37 | 0.0109% | peripheral-nodes | Moderate | d__Bacteria;p__Proteobacteria;c__Betaproteobacteria;o__Methylophilales;f__Methylophilaceae;g__Methylophilus;s__Methylophilus sp. Q8 |
| KF843130.1.1396 | 1.354408 | 0.131715 | II | 44 | 0.0109% | peripheral-nodes | Moderate | d__Bacteria;p__Proteobacteria;c__Gammaproteobacteria;o__Enterobacteriales;f__Enterobacteriaceae;g__Escherichia-Shigella;s__uncultured bacterium |
| KJ147064.1.1497 | -0.76423 | 0 | Other | 2 | 0.0109% | peripheral-nodes | Moderate | d__Bacteria;p__Proteobacteria;c__Gammaproteobacteria;o__Xanthomonadales;f__Xanthomonadaceae;g__Vulcaniibacterium;s__Lysobacter xinjiangensis |
| KF991506.1.1295 | 0.865491 | 0.164082 | II | 35 | 0.0110% | peripheral-nodes | Moderate | d__Bacteria;p__Proteobacteria;c__Gammaproteobacteria;o__Enterobacteriales;f__Enterobacteriaceae;g__Escherichia-Shigella;s__Klebsiella sp. SUS10K |
| BACV01000023.6069.7570 | -0.00369 | 0.209877 | III | 18 | 0.0111% | peripheral-nodes | Moderate | d__Bacteria;p__Bacteroidetes;c__Sphingobacteriia;o__Sphingobacteriales;f__Sphingobacteriaceae;g__Mucilaginibacter;s__Cytophagales bacterium B6 |
| HQ741797.1.1413 | 0.702519 | 0.065556 | IV | 30 | 0.0112% | peripheral-nodes | Moderate | d__Bacteria;p__Firmicutes;c__Clostridia;o__Clostridiales;f__Clostridiaceae 1;g__Clostridium sensu stricto 1;s__uncultured bacterium |
| HQ744264.1.1432 | 0.865491 | 0.114187 | IV | 34 | 0.0114% | peripheral-nodes | Moderate | d__Bacteria;p__Firmicutes;c__Clostridia;o__Clostridiales;f__Clostridiaceae 1;g__Clostridium sensu stricto 1;s__uncultured organism |
| JQ691700.1.1487 | -0.70991 | 0 | VI | 3 | 0.0114% | peripheral-nodes | Moderate | d__Bacteria;p__Proteobacteria;c__Gammaproteobacteria;o__Pseudomonadales;f__Pseudomonadaceae;g__Pseudomonas;s__Pseudomonas fluorescens |
| APIO01000122.50.1579 | -0.70991 | 0 | Other | 3 | 0.0115% | peripheral-nodes | Moderate | d__Bacteria;p__Proteobacteria;c__Gammaproteobacteria;o__Pseudomonadales;f__Pseudomonadaceae;g__Pseudomonas;s__Pseudomonas sp. G5(2012) |
| HM305788.1.1363 | 1.354408 | 0.131715 | II | 44 | 0.0116% | peripheral-nodes | Moderate | d__Bacteria;p__Proteobacteria;c__Gammaproteobacteria;o__Enterobacteriales;f__Enterobacteriaceae;g__Escherichia-Shigella;s__uncultured bacterium |
| KC734214.1.1399 | 1.463057 | 0.126181 | II | 46 | 0.0116% | peripheral-nodes | Moderate | d__Bacteria;p__Proteobacteria;c__Gammaproteobacteria;o__Enterobacteriales;f__Enterobacteriaceae;g__Serratia;s__bacterium 28W115 |
| EU538961.1.1398 | 0.648195 | 0.184183 | I | 31 | 0.0117% | peripheral-nodes | Moderate | d__Bacteria;p__Proteobacteria;c__Gammaproteobacteria;o__Pseudomonadales;f__Pseudomonadaceae;g__Pseudomonas;s__uncultured bacterium |
| FJ673641.1.1371 | 0.702519 | 0.065556 | IV | 30 | 0.0118% | peripheral-nodes | Moderate | d__Bacteria;p__Firmicutes;c__Clostridia;o__Clostridiales;f__Clostridiaceae 1;g__Clostridium sensu stricto 1;s__uncultured bacterium |
| X75273.1.1441 | 1.463057 | 0.126181 | II | 46 | 0.0119% | peripheral-nodes | Moderate | d__Bacteria;p__Proteobacteria;c__Gammaproteobacteria;o__Enterobacteriales;f__Enterobacteriaceae;g__Yersinia;s__Yersinia frederiksenii |
| JUXF01000227.26.1547 | -0.81856 | 0 | II | 1 | 0.0119% | peripheral-nodes | Moderate | d__Bacteria;p__Proteobacteria;c__Gammaproteobacteria;o__Enterobacteriales;f__Enterobacteriaceae;g__Serratia;s__Serratia marcescens |
| FJ685461.1.1348 | -0.22099 | 0 | IV | 12 | 0.0119% | peripheral-nodes | Moderate | d__Bacteria;p__Firmicutes;c__Clostridia;o__Clostridiales;f__Clostridiaceae 1;g__Clostridium sensu stricto 1;s__uncultured bacterium |
| JN867377.1.1527 | 0.865491 | 0.114187 | I | 34 | 0.0119% | peripheral-nodes | Moderate | d__Bacteria;p__Proteobacteria;c__Gammaproteobacteria;o__Pseudomonadales;f__Pseudomonadaceae;g__Pseudomonas;s__uncultured bacterium |
| EU452231.1.1430 | -0.11234 | 0.128889 | VIII | 15 | 0.0120% | peripheral-nodes | Moderate | d__Bacteria;p__Firmicutes;c__Bacilli;o__Lactobacillales;f__Lactobacillaceae;g__Lactobacillus;s__uncultured bacterium |
| KF077988.1.1363 | -0.56549 | 0.998462 | II | 51 | 0.0120% | connectors | Moderate | d__Bacteria;p__Proteobacteria;c__Gammaproteobacteria;o__Enterobacteriales;f__Enterobacteriaceae;g__Serratia;s__uncultured bacterium |
| HQ795689.1.1458 | 1.571705 | 0.121094 | II | 48 | 0.0122% | peripheral-nodes | Moderate | d__Bacteria;p__Proteobacteria;c__Gammaproteobacteria;o__Enterobacteriales;f__Enterobacteriaceae;g__Escherichia-Shigella;s__uncultured organism |
| AJ289163.1.1487 | -0.65558 | 0.36 | I | 5 | 0.0123% | peripheral-nodes | Moderate | d__Bacteria;p__Proteobacteria;c__Gammaproteobacteria;o__Cellvibrionales;f__Cellvibrionaceae;g__Cellvibrio;s__Cellvibrio sp. R-4075 |
| JAEQ01000018.182.1646 | -0.76423 | 0.555556 | IV | 3 | 0.0125% | peripheral-nodes | Moderate | d__Bacteria;p__Proteobacteria;c__Alphaproteobacteria;o__Rhizobiales;f__Phyllobacteriaceae;g__norank;s__Chelativorans sp. J32 |
| JRMY01000047.88.1633 | -0.81856 | 0 | Other | 1 | 0.0125% | peripheral-nodes | Moderate | d__Bacteria;p__Firmicutes;c__Clostridia;o__Clostridiales;f__Family XI;g__Ezakiella;s__Tissierellia bacterium S7-1-4 |
| EU772602.1.1240 | -0.76423 | 0 | II | 2 | 0.0126% | peripheral-nodes | Moderate | d__Bacteria;p__Proteobacteria;c__Gammaproteobacteria;o__Enterobacteriales;f__Enterobacteriaceae;g__Escherichia-Shigella;s__uncultured bacterium |
| EU469741.1.1396 | 0.811167 | 0.168685 | I | 34 | 0.0128% | peripheral-nodes | Moderate | d__Bacteria;p__Proteobacteria;c__Gammaproteobacteria;o__Pseudomonadales;f__Pseudomonadaceae;g__Pseudomonas;s__uncultured bacterium |
| AIFW01000001.288112.289642 | 0.593871 | 0.19 | II | 30 | 0.0129% | peripheral-nodes | Moderate | d__Bacteria;p__Proteobacteria;c__Gammaproteobacteria;o__Enterobacteriales;f__Enterobacteriaceae;g__Escherichia-Shigella;s__Escherichia coli DEC6B |
| EU772607.1.1332 | -1.01036 | 1 | Other | 1 | 0.0129% | connectors | Moderate | d__Bacteria;p__Proteobacteria;c__Gammaproteobacteria;o__Enterobacteriales;f__Enterobacteriaceae;g__Escherichia-Shigella;s__uncultured bacterium |
| KC210871.1.1428 | 1.24576 | 0.137755 | II | 42 | 0.0130% | peripheral-nodes | Moderate | d__Bacteria;p__Proteobacteria;c__Gammaproteobacteria;o__Enterobacteriales;f__Enterobacteriaceae;g__Hafnia-Obesumbacterium;s__Hafnia alvei |
| JQ236817.1.1439 | 1.028464 | 0.194609 | I | 39 | 0.0130% | peripheral-nodes | Moderate | d__Bacteria;p__Proteobacteria;c__Gammaproteobacteria;o__Pseudomonadales;f__Pseudomonadaceae;g__Pseudomonas;s__bacterium hswX133 |
| HM343520.1.1360 | -0.81856 | 0 | I | 1 | 0.0130% | peripheral-nodes | Moderate | d__Bacteria;p__Bacteroidetes;c__Sphingobacteriia;o__Sphingobacteriales;f__Sphingobacteriaceae;g__Sphingobacterium;s__uncultured bacterium |
| CAHF01000002.3754.5264 | 1.24576 | 0.21436 | I | 44 | 0.0131% | peripheral-nodes | Moderate | d__Bacteria;p__Proteobacteria;c__Betaproteobacteria;o__Burkholderiales;f__Oxalobacteraceae;g__Herbaspirillum;s__Herbaspirillum massiliense JC206 |
| KM187631.1.1402 | 0.593871 | 0.19 | II | 30 | 0.0131% | peripheral-nodes | Moderate | d__Bacteria;p__Proteobacteria;c__Gammaproteobacteria;o__Enterobacteriales;f__Enterobacteriaceae;g__Hafnia-Obesumbacterium;s__Enterobacteriaceae bacterium PRE7B |
| EF151505.1.1509 | -0.54694 | 0 | VI | 6 | 0.0133% | peripheral-nodes | Moderate | d__Bacteria;p__Actinobacteria;c__Actinobacteria;o__Micrococcales;f__Micrococcaceae;g__Nesterenkonia;s__Nesterenkonia sp. YIM 90713 |
| HQ747801.1.1417 | 0.702519 | 0.12487 | IV | 31 | 0.0133% | peripheral-nodes | Moderate | d__Bacteria;p__Firmicutes;c__Clostridia;o__Clostridiales;f__Clostridiaceae 1;g__Clostridium sensu stricto 1;s__uncultured organism |
| KM924548.1.1437 | -0.60126 | 0.305556 | V | 6 | 0.0134% | peripheral-nodes | Moderate | d__Bacteria;p__Proteobacteria;c__Alphaproteobacteria;o__Rhizobiales;f__1174-901-12;g__norank;s__Rhizobiales bacterium 8433D |
| APFF01000036.3669.5181 | 0.919815 | 0.284024 | I | 39 | 0.0138% | peripheral-nodes | Moderate | d__Bacteria;p__Proteobacteria;c__Betaproteobacteria;o__Burkholderiales;f__Oxalobacteraceae;g__Massilia;s__Janthinobacterium sp. CG3 |
| LETU01000019.3467.4996 | 1.24576 | 0.137755 | II | 42 | 0.0138% | peripheral-nodes | Moderate | d__Bacteria;p__Proteobacteria;c__Gammaproteobacteria;o__Enterobacteriales;f__Enterobacteriaceae;g__Escherichia-Shigella;s__Escherichia coli |
| EU469687.1.1292 | -0.65558 | 0 | V | 4 | 0.0138% | peripheral-nodes | Moderate | d__Bacteria;p__Firmicutes;c__Clostridia;o__Clostridiales;f__Clostridiaceae 1;g__Clostridium sensu stricto 1;s__uncultured bacterium |
| JX515435.1.1445 | 1.028464 | 0.194609 | I | 39 | 0.0140% | peripheral-nodes | Moderate | d__Bacteria;p__Proteobacteria;c__Alphaproteobacteria;o__Rhizobiales;f__Brucellaceae;g__Pseudochrobactrum;s__uncultured bacterium |
| EF509349.1.1494 | -0.8765 | 1 | I | 1 | 0.0140% | connectors | Moderate | d__Bacteria;p__Bacteroidetes;c__Flavobacteriia;o__Flavobacteriales;f__Flavobacteriaceae;g__Myroides;s__uncultured bacterium |
| GQ359984.1.1464 | -0.70991 | 0 | Other | 3 | 0.0142% | peripheral-nodes | Moderate | d__Bacteria;p__Firmicutes;c__Clostridia;o__Clostridiales;f__Peptostreptococcaceae;g__Terrisporobacter;s__uncultured bacterium |
| EU452382.1.1427 | -0.11234 | 0.128889 | VIII | 15 | 0.0142% | peripheral-nodes | Moderate | d__Bacteria;p__Firmicutes;c__Bacilli;o__Lactobacillales;f__Lactobacillaceae;g__Lactobacillus;s__uncultured bacterium |
| JHVA01000001.4067904.4069383 | -0.43829 | 0 | V | 8 | 0.0143% | peripheral-nodes | Moderate | d__Bacteria;p__Acidobacteria;c__Acidobacteria;o__Acidobacteriales;f__Acidobacteriaceae (Subgroup 1);g__Edaphobacter;s__Acidobacteria bacterium KBS 146 |
| AZJT01000071.1165.2796 | -0.87288 | 1 | V | 1 | 0.0144% | connectors | Moderate | d__Bacteria;p__Firmicutes;c__Bacilli;o__Lactobacillales;f__Streptococcaceae;g__Streptococcus;s__Streptococcus thermophilus M17PTZA496 |
| DQ809413.1.1374 | 0.430898 | 0.0784 | IV | 25 | 0.0145% | peripheral-nodes | Moderate | d__Bacteria;p__Firmicutes;c__Clostridia;o__Clostridiales;f__Clostridiaceae 1;g__Clostridium sensu stricto 1;s__uncultured bacterium |
| LELH02000031.11452.12993 | -0.76423 | 0 | I | 2 | 0.0145% | peripheral-nodes | Moderate | d__Bacteria;p__Proteobacteria;c__Betaproteobacteria;o__Burkholderiales;f__Oxalobacteraceae;g__Massilia;s__Massilia sp. WF1 |
| LQXW01000075.88.1634 | -0.64784 | 1 | Other | 2 | 0.0145% | connectors | Moderate | d__Bacteria;p__Proteobacteria;c__Gammaproteobacteria;o__Pseudomonadales;f__Pseudomonadaceae;g__Pseudomonas;s__Pseudomonas sp. ABFPK |
| AM421976.1.1498 | 0.811167 | 0.061523 | I | 32 | 0.0147% | peripheral-nodes | Moderate | d__Bacteria;p__Proteobacteria;c__Gammaproteobacteria;o__Pseudomonadales;f__Pseudomonadaceae;g__Pseudomonas;s__Pseudomonas sp. NJ-24 |
| JF920371.1.1345 | -0.81856 | 0 | Other | 1 | 0.0147% | peripheral-nodes | Moderate | d__Bacteria;p__Firmicutes;c__Clostridia;o__Clostridiales;f__Lachnospiraceae;g__Epulopiscium;s__uncultured bacterium |
| EU460258.1.1401 | 0.32225 | 0.2256 | II | 25 | 0.0148% | peripheral-nodes | Moderate | d__Bacteria;p__Proteobacteria;c__Gammaproteobacteria;o__Enterobacteriales;f__Enterobacteriaceae;g__Plesiomonas;s__uncultured bacterium |
| LOSF01000001.4080205.4081760 | 0.97414 | 0.108025 | II | 36 | 0.0149% | peripheral-nodes | Moderate | d__Bacteria;p__Proteobacteria;c__Gammaproteobacteria;o__Enterobacteriales;f__Enterobacteriaceae;g__Escherichia-Shigella;s__Shigella sonnei |
| ATWL01000047.929.2440 | -0.81856 | 0 | Other | 1 | 0.0152% | peripheral-nodes | Moderate | d__Bacteria;p__Actinobacteria;c__Actinobacteria;o__Micrococcales;f__Ruaniaceae;g__Ruania;s__Ruania albidiflava DSM 18029 |
| KF105166.1.1391 | -0.11234 | 0.128889 | VIII | 15 | 0.0153% | peripheral-nodes | Moderate | d__Bacteria;p__Firmicutes;c__Bacilli;o__Lactobacillales;f__Lactobacillaceae;g__Lactobacillus;s__uncultured bacterium |
| JAUG01000180.350.1851 | 1.24576 | 0.281191 | I | 46 | 0.0155% | peripheral-nodes | Moderate | d__Bacteria;p__Bacteroidetes;c__Sphingobacteriia;o__Sphingobacteriales;f__Sphingobacteriaceae;g__Pedobacter;s__Pedobacter borealis DSM 19626 |
| AYXT01000013.423283.424892 | 2.060622 | 0.21634 | I | 61 | 0.0158% | peripheral-nodes | Moderate | d__Bacteria;p__Proteobacteria;c__Betaproteobacteria;o__Burkholderiales;f__Alcaligenaceae;g__Advenella;s__Advenella kashmirensis W13003 |
| JQ428707.1.1442 | -0.81856 | 0 | Other | 1 | 0.0161% | peripheral-nodes | Moderate | d__Bacteria;p__Proteobacteria;c__Alphaproteobacteria;o__Rhizobiales;f__Phyllobacteriaceae;g__uncultured;s__uncultured bacterium |
| JN873172.1.1240 | -0.64784 | 1 | VII | 1 | 0.0161% | connectors | Moderate | d__Bacteria;p__Firmicutes;c__Clostridia;o__Clostridiales;f__Clostridiaceae 1;g__Clostridium sensu stricto 1;s__Clostridium sp. KOPRI80152 |
| JNFF01000078.74.1510 | 1.789002 | 0.234375 | I | 56 | 0.0165% | peripheral-nodes | Moderate | d__Bacteria;p__Bacteroidetes;c__Sphingobacteriia;o__Sphingobacteriales;f__Sphingobacteriaceae;g__Pedobacter;s__Pedobacter antarcticus 4BY |
| EU535141.1.1394 | -0.65558 | 0 | Other | 4 | 0.0167% | peripheral-nodes | Moderate | d__Bacteria;p__Proteobacteria;c__Gammaproteobacteria;o__Pseudomonadales;f__Pseudomonadaceae;g__Pseudomonas;s__uncultured bacterium |
| EU474075.1.1405 | -1.01036 | 1 | V | 5 | 0.0171% | connectors | Moderate | d__Bacteria;p__Firmicutes;c__Bacilli;o__Lactobacillales;f__Streptococcaceae;g__Streptococcus;s__uncultured bacterium |
| HQ449706.1.1408 | -0.32964 | 0.305556 | III | 12 | 0.0172% | peripheral-nodes | Moderate | d__Bacteria;p__Bacteroidetes;c__Sphingobacteriia;o__Sphingobacteriales;f__Sphingobacteriaceae;g__Mucilaginibacter;s__Mucilaginibacter sp. HME6837 |
| JF162619.1.1357 | 1.680353 | 0.1164 | I | 50 | 0.0173% | peripheral-nodes | Moderate | d__Bacteria;p__Proteobacteria;c__Gammaproteobacteria;o__Pseudomonadales;f__Pseudomonadaceae;g__Pseudomonas;s__uncultured bacterium |
| AEZJ02000039.521.2062 | 2.114946 | 0.100773 | II | 58 | 0.0173% | peripheral-nodes | Moderate | d__Bacteria;p__Proteobacteria;c__Gammaproteobacteria;o__Enterobacteriales;f__Enterobacteriaceae;g__Escherichia-Shigella;s__Escherichia coli 97.0246 |
| HK240782.1.1332 | -0.87288 | 1 | Other | 1 | 0.0174% | connectors | Moderate | d__Bacteria;p__Firmicutes;c__Clostridia;o__Clostridiales;f__Clostridiaceae 1;g__Clostridium sensu stricto 1;s__unidentified |
| GQ069620.1.1358 | 1.680353 | 0.1164 | I | 50 | 0.0179% | peripheral-nodes | Moderate | d__Bacteria;p__Proteobacteria;c__Gammaproteobacteria;o__Pseudomonadales;f__Pseudomonadaceae;g__Pseudomonas;s__uncultured bacterium |
| JQ655794.1.1496 | 0.865491 | 0.252009 | III | 37 | 0.0180% | peripheral-nodes | Moderate | d__Bacteria;p__Verrucomicrobia;c__Opitutae;o__Opitutae vadinHA64;f__norank;g__norank;s__uncultured bacterium |
| AYOD01000070.271.1739 | -0.38396 | 0.19 | IV | 10 | 0.0183% | peripheral-nodes | Moderate | d__Bacteria;p__Proteobacteria;c__Alphaproteobacteria;o__Rhizobiales;f__Phyllobacteriaceae;g__Aliihoeflea;s__Aliihoeflea sp. 2WW |
| HM045828.1.1503 | -0.65555 | 1 | I | 38 | 0.0184% | connectors | Moderate | d__Bacteria;p__Proteobacteria;c__Gammaproteobacteria;o__Pseudomonadales;f__Pseudomonadaceae;g__Pseudomonas;s__Pseudomonas sp. WJ04 |
| KF066673.1.1358 | -0.60126 | 0 | I | 5 | 0.0184% | peripheral-nodes | Moderate | d__Bacteria;p__Proteobacteria;c__Betaproteobacteria;o__Burkholderiales;f__Comamonadaceae;g__Diaphorobacter;s__uncultured bacterium |
| EU468251.1.1323 | -0.81856 | 0 | VI | 1 | 0.0184% | peripheral-nodes | Moderate | d__Bacteria;p__Firmicutes;c__Clostridia;o__Clostridiales;f__Clostridiaceae 1;g__Clostridium sensu stricto 1;s__uncultured bacterium |
| DQ177470.1.1434 | -0.54694 | 0 | III | 6 | 0.0187% | peripheral-nodes | Moderate | d__Bacteria;p__Proteobacteria;c__Betaproteobacteria;o__Burkholderiales;f__Oxalobacteraceae;g__Massilia;s__Janthinobacterium sp. Tibet-IIK43 |
| LN558643.1.1484 | 1.24576 | 0.137755 | II | 42 | 0.0191% | peripheral-nodes | Moderate | d__Bacteria;p__Proteobacteria;c__Gammaproteobacteria;o__Enterobacteriales;f__Enterobacteriaceae;g__Escherichia-Shigella;s__Escherichia coli |
| KC433599.1.1410 | -0.70991 | 0 | Other | 3 | 0.0198% | peripheral-nodes | Moderate | d__Bacteria;p__Proteobacteria;c__Gammaproteobacteria;o__Pseudomonadales;f__Pseudomonadaceae;g__Pseudomonas;s__Pseudomonas sp. 6PC2 |
| AYZX01097562.161.1662 | 2.114946 | 0.237843 | I | 63 | 0.0199% | peripheral-nodes | Moderate | d__Bacteria;p__Bacteroidetes;c__Sphingobacteriia;o__Sphingobacteriales;f__Sphingobacteriaceae;g__Pedobacter;s__Beta vulgaris subsp. vulgaris |
| JQ816944.1.1482 | -0.65558 | 0 | II | 4 | 0.0202% | peripheral-nodes | Moderate | d__Bacteria;p__Proteobacteria;c__Gammaproteobacteria;o__Enterobacteriales;f__Enterobacteriaceae;g__Escherichia-Shigella;s__uncultured bacterium |
| HM140707.1.1427 | -0.49261 | 0 | I | 7 | 0.0202% | peripheral-nodes | Moderate | d__Bacteria;p__Proteobacteria;c__Betaproteobacteria;o__Burkholderiales;f__Comamonadaceae;g__Comamonas;s__Comamonas sp. SJM |
| GQ014216.1.1367 | -0.76423 | 0 | Other | 2 | 0.0210% | peripheral-nodes | Moderate | d__Bacteria;p__Bacteroidetes;c__Bacteroidia;o__Bacteroidales;f__Porphyromonadaceae;g__Porphyromonas;s__uncultured bacterium |
| AUAA01000078.1985.3481 | -0.81856 | 0 | Other | 1 | 0.0212% | peripheral-nodes | Moderate | d__Bacteria;p__Bacteroidetes;c__Flavobacteriia;o__Flavobacteriales;f__Flavobacteriaceae;g__Epilithonimonas;s__Epilithonimonas tenax DSM 16811 |
| AB753963.1.1449 | 1.354408 | 0.270399 | III | 48 | 0.0215% | peripheral-nodes | Moderate | d__Bacteria;p__Bacteroidetes;c__Flavobacteriia;o__Flavobacteriales;f__Cryomorphaceae;g__Fluviicola;s__uncultured bacterium |
| AF538774.1.1469 | -0.81856 | 0 | Other | 1 | 0.0218% | peripheral-nodes | Moderate | d__Bacteria;p__Bacteroidetes;c__Flavobacteriia;o__Flavobacteriales;f__Flavobacteriaceae;g__Chryseobacterium;s__enrichment culture bacterium LB-Q |
| EF102863.1.1450 | 1.082788 | 0.147929 | II | 39 | 0.0224% | peripheral-nodes | Moderate | d__Bacteria;p__Proteobacteria;c__Gammaproteobacteria;o__Enterobacteriales;f__Enterobacteriaceae;g__Serratia;s__Serratia marcescens |
| KM268031.1.1393 | -0.70991 | 0.4375 | V | 4 | 0.0224% | peripheral-nodes | Moderate | d__Bacteria;p__Firmicutes;c__Clostridia;o__Clostridiales;f__Clostridiaceae 1;g__Clostridium sensu stricto 1;s__uncultured soil bacterium |
| GDIP01007208.1.1371 | -0.49261 | 0.234375 | I | 8 | 0.0225% | peripheral-nodes | Moderate | d__Bacteria;p__Proteobacteria;c__Betaproteobacteria;o__Burkholderiales;f__Comamonadaceae;g__Variovorax;s__Daphnia magna |
| BAEW01000035.25.1529 | -0.81856 | 0 | Other | 1 | 0.0225% | peripheral-nodes | Moderate | d__Bacteria;p__Firmicutes;c__Clostridia;o__Clostridiales;f__Family XI;g__Peptoniphilus;s__Peptoniphilus rhinitidis 1-13 |
| JN637322.1.1364 | 2.766836 | 0.201956 | I | 75 | 0.0230% | module-hubs | Moderate | d__Bacteria;p__Bacteroidetes;c__Cytophagia;o__Cytophagales;f__Cytophagaceae;g__Dyadobacter;s__Dyadobacter sp. hp19 |
| LNIY01000034.3620.5109 | -0.16667 | 0.415225 | III | 17 | 0.0231% | peripheral-nodes | Moderate | d__Bacteria;p__Proteobacteria;c__Alphaproteobacteria;o__Caulobacterales;f__Caulobacteraceae;g__Caulobacter;s__Caulobacter vibrioides |
| AQEX01000006.691.2218 | -0.81856 | 0 | II | 1 | 0.0235% | peripheral-nodes | Moderate | d__Bacteria;p__Proteobacteria;c__Gammaproteobacteria;o__Enterobacteriales;f__Enterobacteriaceae;g__Escherichia-Shigella;s__Escherichia coli MP021017.1 |
| EU772369.1.1408 | -0.76423 | 0 | II | 2 | 0.0236% | peripheral-nodes | Moderate | d__Bacteria;p__Firmicutes;c__Bacilli;o__Lactobacillales;f__Streptococcaceae;g__Streptococcus;s__uncultured bacterium |
| EU037322.1.1402 | -0.27532 | 0.159722 | IV | 12 | 0.0243% | peripheral-nodes | Moderate | d__Bacteria;p__Proteobacteria;c__Alphaproteobacteria;o__Rhizobiales;f__Phyllobacteriaceae;g__uncultured;s__uncultured bacterium |
| JXOG01000088.370.1916 | -0.43829 | 0.36 | I | 10 | 0.0245% | peripheral-nodes | Moderate | d__Bacteria;p__Proteobacteria;c__Gammaproteobacteria;o__Pseudomonadales;f__Pseudomonadaceae;g__Pseudomonas;s__Pseudomonas putida |
| KF088459.1.1352 | 2.114946 | 0.261475 | I | 64 | 0.0246% | peripheral-nodes | Moderate | d__Bacteria;p__Proteobacteria;c__Betaproteobacteria;o__Burkholderiales;f__Comamonadaceae;g__Acidovorax;s__uncultured bacterium |
| AZTT01000006.153.1694 | -0.81856 | 0 | Other | 1 | 0.0247% | peripheral-nodes | Moderate | d__Bacteria;p__Proteobacteria;c__Gammaproteobacteria;o__Pasteurellales;f__Pasteurellaceae;g__Aggregatibacter;s__Aggregatibacter actinomycetemcomitans serotype e str. SA2149 |
| AJ438170.1.1495 | 0.919815 | 0.204529 | I | 37 | 0.0248% | peripheral-nodes | Moderate | d__Bacteria;p__Bacteroidetes;c__Sphingobacteriia;o__Sphingobacteriales;f__Sphingobacteriaceae;g__Pedobacter;s__Pedobacter cryoconitis |
| LLWP01000001.943790.945315 | 1.300084 | 0.305556 | I | 48 | 0.0254% | peripheral-nodes | Moderate | d__Bacteria;p__Bacteroidetes;c__Sphingobacteriia;o__Sphingobacteriales;f__Sphingobacteriaceae;g__Pedobacter;s__Pedobacter sp. Hv1 |
| JPRH01000001.892910.894464 | 2.277919 | 0.178711 | I | 64 | 0.0255% | peripheral-nodes | Moderate | d__Bacteria;p__Bacteroidetes;c__Flavobacteriia;o__Flavobacteriales;f__Flavobacteriaceae;g__Chryseobacterium;s__Chryseobacterium soli |
| HQ741515.1.1430 | 1.028464 | 0.151662 | IV | 38 | 0.0257% | peripheral-nodes | Moderate | d__Bacteria;p__Firmicutes;c__Clostridia;o__Clostridiales;f__Clostridiaceae 1;g__Clostridium sensu stricto 1;s__uncultured bacterium |
| AY315167.1.1345 | -0.76423 | 0 | III | 2 | 0.0265% | peripheral-nodes | Moderate | d__Bacteria;p__Proteobacteria;c__Alphaproteobacteria;o__Rhizobiales;f__Bradyrhizobiaceae;g__Tardiphaga;s__glacier bacterium FXI3 |
| JUGE01000001.518824.520344 | -0.58867 | 0.999405 | I | 82 | 0.0276% | connectors | Moderate | d__Bacteria;p__Proteobacteria;c__Betaproteobacteria;o__Methylophilales;f__Methylophilaceae;g__Methylophilus;s__Methylotenera sp. N17 |
| HM284700.1.1364 | 2.603863 | 0.114187 | II | 68 | 0.0278% | module-hubs | Moderate | d__Bacteria;p__Proteobacteria;c__Gammaproteobacteria;o__Enterobacteriales;f__Enterobacteriaceae;g__Serratia;s__uncultured bacterium |
| JF218031.1.1361 | -0.76423 | 0 | VII | 2 | 0.0282% | peripheral-nodes | Moderate | d__Bacteria;p__Proteobacteria;c__Gammaproteobacteria;o__Pseudomonadales;f__Moraxellaceae;g__Acinetobacter;s__uncultured bacterium |
| AQGK01000001.1611.3112 | 3.310077 | 0.19835 | I | 86 | 0.0286% | module-hubs | Moderate | d__Bacteria;p__Bacteroidetes;c__Sphingobacteriia;o__Sphingobacteriales;f__Sphingobacteriaceae;g__Pedobacter;s__Pedobacter heparinus DSM 2366 |
| AWRU01000031.174562.176089 | -0.56549 | 0.994083 | I | 26 | 0.0296% | connectors | Moderate | d__Bacteria;p__Bacteroidetes;c__Sphingobacteriia;o__Sphingobacteriales;f__Sphingobacteriaceae;g__Pedobacter;s__Pedobacter sp. V48 |
| HM317247.1.1350 | -0.22099 | 0 | I | 12 | 0.0299% | peripheral-nodes | Moderate | d__Bacteria;p__Proteobacteria;c__Betaproteobacteria;o__Burkholderiales;f__Comamonadaceae;g__Acidovorax;s__uncultured bacterium |
| AB680202.1.1472 | 0.702519 | 0.272491 | I | 34 | 0.0304% | peripheral-nodes | Moderate | d__Bacteria;p__Proteobacteria;c__Gammaproteobacteria;o__Xanthomonadales;f__Xanthomonadaceae;g__Xanthomonas;s__Xanthomonas oryzae |
| AY294221.1.1465 | -0.56549 | 0.999419 | III | 83 | 0.0305% | connectors | Moderate | d__Bacteria;p__Proteobacteria;c__Deltaproteobacteria;o__Bdellovibrionales;f__Bacteriovoracaceae;g__Bacteriovorax;s__Bacteriovorax sp. PNEc1 |
| EU473362.1.1385 | -0.8765 | 1 | V | 2 | 0.0307% | connectors | Moderate | d__Bacteria;p__Firmicutes;c__Clostridia;o__Clostridiales;f__Clostridiaceae 1;g__Clostridium sensu stricto 1;s__uncultured bacterium |
| HM777013.1.1406 | 0.267926 | 0.234375 | III | 24 | 0.0310% | peripheral-nodes | Moderate | d__Bacteria;p__Proteobacteria;c__Betaproteobacteria;o__Burkholderiales;f__Oxalobacteraceae;g__Massilia;s__Massilia flava |
| AZTZ01000011.241.1785 | -0.81856 | 0 | Other | 1 | 0.0313% | peripheral-nodes | Moderate | d__Bacteria;p__Proteobacteria;c__Gammaproteobacteria;o__Pasteurellales;f__Pasteurellaceae;g__Aggregatibacter;s__Aggregatibacter actinomycetemcomitans serotype e str. ANH9776 |
| ATZW02000042.2256.3770 | 2.114946 | 0.237843 | I | 63 | 0.0320% | peripheral-nodes | Moderate | d__Bacteria;p__Proteobacteria;c__Betaproteobacteria;o__Burkholderiales;f__Comamonadaceae;g__Xylophilus;s__Bacillus sp. JGI 001011-F15 |
| GAXI01005455.1.1233 | 0.593871 | 0.241415 | I | 31 | 0.0321% | peripheral-nodes | Moderate | d__Bacteria;p__Bacteroidetes;c__Flavobacteriia;o__Flavobacteriales;f__Flavobacteriaceae;g__Chryseobacterium;s__Tetrodontophora bielanensis (giant springtail) |
| HAAB01020513.1.1384 | -0.70991 | 0 | I | 3 | 0.0321% | peripheral-nodes | Moderate | d__Bacteria;p__Proteobacteria;c__Betaproteobacteria;o__Burkholderiales;f__Oxalobacteraceae;g__Duganella;s__Triticum aestivum (bread wheat) |
| KF086163.1.1367 | -0.76423 | 0 | Other | 2 | 0.0324% | peripheral-nodes | Moderate | d__Bacteria;p__Bacteroidetes;c__Bacteroidia;o__Bacteroidales;f__Porphyromonadaceae;g__Porphyromonas;s__uncultured bacterium |
| KF066097.1.1358 | -0.70991 | 0 | VII | 3 | 0.0330% | peripheral-nodes | Moderate | d__Bacteria;p__Proteobacteria;c__Gammaproteobacteria;o__Pseudomonadales;f__Moraxellaceae;g__Acinetobacter;s__uncultured bacterium |
| LCTB01000191.2.1381 | 2.440891 | 0.218442 | I | 69 | 0.0333% | peripheral-nodes | Moderate | d__Bacteria;p__Proteobacteria;c__Betaproteobacteria;o__Burkholderiales;f__Comamonadaceae;g__Pseudorhodoferax;s__Pseudomonas aeruginosa |
| KR047732.1.1423 | 1.028464 | 0.234375 | I | 40 | 0.0336% | peripheral-nodes | Moderate | d__Bacteria;p__Bacteroidetes;c__Flavobacteriia;o__Flavobacteriales;f__Flavobacteriaceae;g__Flavobacterium;s__Flavobacterium sp. JM-222 |
| JF920368.1.1360 | -0.81856 | 0 | VII | 1 | 0.0341% | peripheral-nodes | Moderate | d__Bacteria;p__Firmicutes;c__Clostridia;o__Clostridiales;f__Lachnospiraceae;g__Cellulosilyticum;s__uncultured bacterium |
| GBZK01035487.2.1773 | -0.81856 | 0 | Other | 1 | 0.0346% | peripheral-nodes | Moderate | d__Bacteria;p__Proteobacteria;c__Alphaproteobacteria;o__Rickettsiales;f__Mitochondria;g__norank;s__Calotropis procera |
| JMDZ01000013.7280.8800 | 2.875484 | 0.217456 | I | 78 | 0.0357% | module-hubs | Moderate | d__Bacteria;p__Proteobacteria;c__Betaproteobacteria;o__Burkholderiales;f__Comamonadaceae;g__Polaromonas;s__Polaromonas glacialis |
| LVWW01000020.134.1648 | -0.87288 | 1 | VIII | 1 | 0.0365% | connectors | Moderate | d__Bacteria;p__Firmicutes;c__Bacilli;o__Lactobacillales;f__Streptococcaceae;g__Streptococcus;s__Streptococcus salivarius subsp. thermophilus |
| LMPU01000009.953875.955405 | 1.89765 | 0.226813 | I | 58 | 0.0375% | peripheral-nodes | Moderate | d__Bacteria;p__Bacteroidetes;c__Sphingobacteriia;o__Sphingobacteriales;f__Sphingobacteriaceae;g__Pedobacter;s__Pedobacter sp. Leaf194 |
| AM930377.1.1461 | -0.60126 | 0 | VI | 5 | 0.0379% | peripheral-nodes | Moderate | d__Bacteria;p__Firmicutes;c__Clostridia;o__Clostridiales;f__Peptostreptococcaceae;g__Peptoclostridium;s__uncultured bacterium |
| JUGK01000006.967.2463 | 1.680353 | 0.213599 | I | 53 | 0.0380% | peripheral-nodes | Moderate | d__Bacteria;p__Bacteroidetes;c__Flavobacteriia;o__Flavobacteriales;f__Flavobacteriaceae;g__Chryseobacterium;s__Chryseobacterium sp. YR005 |
| ACHT01000508.52948.54474 | 0.648195 | 0.184183 | I | 31 | 0.0384% | peripheral-nodes | Moderate | d__Bacteria;p__Proteobacteria;c__Gammaproteobacteria;o__Xanthomonadales;f__Xanthomonadaceae;g__Xanthomonas;s__Xanthomonas campestris pv. musacearum NCPPB 4381 |
| JX029047.1.1223 | 2.060622 | 0.133175 | II | 58 | 0.0390% | peripheral-nodes | Moderate | d__Bacteria;p__Proteobacteria;c__Gammaproteobacteria;o__Enterobacteriales;f__Enterobacteriaceae;g__Escherichia-Shigella;s__Pectobacterium carotovorum subsp. carotovorum |
| GQ921467.1.1497 | -0.76423 | 0 | VI | 2 | 0.0399% | peripheral-nodes | Moderate | d__Bacteria;p__Proteobacteria;c__Gammaproteobacteria;o__Enterobacteriales;f__Enterobacteriaceae;g__Klebsiella;s__uncultured bacterium |
| GU935301.1.1457 | -0.65558 | 0 | VII | 4 | 0.0419% | peripheral-nodes | Moderate | d__Bacteria;p__Firmicutes;c__Bacilli;o__Bacillales;f__Planococcaceae;g__Lysinibacillus;s__Lysinibacillus sp. EK-I65 |
| AB369002.1.1355 | -0.54694 | 0 | VII | 6 | 0.0424% | peripheral-nodes | Moderate | d__Bacteria;p__Proteobacteria;c__Gammaproteobacteria;o__Pseudomonadales;f__Moraxellaceae;g__Acinetobacter;s__uncultured bacterium |
| AY534872.1.1442 | -0.60126 | 0.305556 | VII | 6 | 0.0429% | peripheral-nodes | Moderate | d__Bacteria;p__Firmicutes;c__Clostridia;o__Clostridiales;f__Lachnospiraceae;g__Anaerosporobacter;s__Anaerosporobacter mobilis |
| HQ758829.1.1473 | 2.277919 | 0.095942 | II | 61 | 0.0429% | peripheral-nodes | Moderate | d__Bacteria;p__Proteobacteria;c__Gammaproteobacteria;o__Enterobacteriales;f__Enterobacteriaceae;g__Escherichia-Shigella;s__uncultured organism |
| EU636044.1.1427 | 1.571705 | 0.19 | I | 50 | 0.0431% | peripheral-nodes | Moderate | d__Bacteria;p__Proteobacteria;c__Betaproteobacteria;o__Burkholderiales;f__Oxalobacteraceae;g__Massilia;s__Antarctic bacterium GA057 |
| JN688048.1.1427 | -0.76423 | 0 | VI | 2 | 0.0450% | peripheral-nodes | Moderate | d__Bacteria;p__Firmicutes;c__Clostridia;o__Clostridiales;f__Peptostreptococcaceae;g__Terrisporobacter;s__Clostridium sp. C1 |
| JQ200645.1.1346 | -0.81856 | 0 | Other | 1 | 0.0452% | peripheral-nodes | Moderate | d__Bacteria;p__Firmicutes;c__Clostridia;o__Clostridiales;f__Clostridiaceae 1;g__Clostridium sensu stricto 1;s__uncultured bacterium |
| JQ606917.1.1425 | 0.104953 | 0.19 | II | 20 | 0.0460% | peripheral-nodes | Moderate | d__Bacteria;p__Proteobacteria;c__Gammaproteobacteria;o__Enterobacteriales;f__Enterobacteriaceae;g__Escherichia-Shigella;s__bacterium NLAE-zl-P253 |
| LKKK01000002.3256.4801 | -0.43829 | 0 | I | 8 | 0.0462% | peripheral-nodes | Moderate | d__Bacteria;p__Proteobacteria;c__Gammaproteobacteria;o__Pseudomonadales;f__Pseudomonadaceae;g__Pseudomonas;s__Pseudomonas sp. TTU2014-080ASC |
| HQ018739.1.1317 | 1.028464 | 0.234375 | I | 40 | 0.0462% | peripheral-nodes | Moderate | d__Bacteria;p__Proteobacteria;c__Gammaproteobacteria;o__Pseudomonadales;f__Pseudomonadaceae;g__Pseudomonas;s__Pseudomonas chlororaphis subsp. aureofaciens |
| LN881572.1.1253 | -0.65555 | 1 | Other | 1 | 0.0463% | connectors | Moderate | d__Bacteria;p__Firmicutes;c__Clostridia;o__Clostridiales;f__Clostridiaceae 1;g__Clostridium sensu stricto 1;s__Clostridium isatidis |
| FJ679064.1.1424 | -0.81856 | 0 | Other | 1 | 0.0468% | peripheral-nodes | Moderate | d__Bacteria;p__Firmicutes;c__Clostridia;o__Clostridiales;f__Clostridiaceae 1;g__Clostridium sensu stricto 1;s__uncultured bacterium |
| ADUU01000034.39129.40654 | -0.43829 | 0.209877 | II | 9 | 0.0488% | peripheral-nodes | Moderate | d__Bacteria;p__Proteobacteria;c__Gammaproteobacteria;o__Enterobacteriales;f__Enterobacteriaceae;g__Escherichia-Shigella;s__Shigella sonnei 53G |
| KC710044.1.1425 | -0.11234 | 0 | I | 14 | 0.0488% | peripheral-nodes | Moderate | d__Bacteria;p__Proteobacteria;c__Betaproteobacteria;o__Burkholderiales;f__Comamonadaceae;g__Lampropedia;s__Lampropedia sp. 10925 |
| HQ807166.1.1464 | -0.00369 | 0.114187 | VIII | 17 | 0.0495% | peripheral-nodes | Moderate | d__Bacteria;p__Firmicutes;c__Bacilli;o__Lactobacillales;f__Lactobacillaceae;g__Lactobacillus;s__uncultured organism |
| AMWD01000001.1163191.1164707 | 1.408733 | 0.201449 | I | 47 | 0.0514% | peripheral-nodes | Moderate | d__Bacteria;p__Proteobacteria;c__Betaproteobacteria;o__Burkholderiales;f__Oxalobacteraceae;g__Duganella;s__Janthinobacterium sp. HH01 |
| CACS02000061.1.1476 | -0.81856 | 0.75 | VIII | 2 | 0.0515% | connectors | Moderate | d__Bacteria;p__Firmicutes;c__Bacilli;o__Lactobacillales;f__Lactobacillaceae;g__Lactobacillus;s__Lactobacillus reuteri ATCC 53608 |
| JQ084711.1.1533 | -0.00369 | 0.114187 | VIII | 17 | 0.0523% | peripheral-nodes | Moderate | d__Bacteria;p__Firmicutes;c__Bacilli;o__Lactobacillales;f__Lactobacillaceae;g__Lactobacillus;s__uncultured bacterium |
| CAPG01000101.61.1390 | -0.65555 | 1 | Other | 1 | 0.0540% | connectors | Moderate | d__Bacteria;p__Firmicutes;c__Bacilli;o__Bacillales;f__Bacillaceae;g__Bacillus;s__Bacillus massilioanorexius AP8 |
| DQ796133.1.1287 | -0.60126 | 0.305556 | V | 6 | 0.0557% | peripheral-nodes | Moderate | d__Bacteria;p__Firmicutes;c__Clostridia;o__Clostridiales;f__Clostridiaceae 1;g__Clostridium sensu stricto 1;s__uncultured bacterium |
| CBLL010000172.6.1514 | -0.38396 | 0.19 | IV | 10 | 0.0572% | peripheral-nodes | Moderate | d__Bacteria;p__Actinobacteria;c__Actinobacteria;o__Micrococcales;f__Micrococcaceae;g__Nesterenkonia;s__Nesterenkonia sp. NP1 |
| BCUS01000208.467.1981 | -0.58867 | 0.99939 | I | 81 | 0.0582% | connectors | Moderate | d__Bacteria;p__Proteobacteria;c__Betaproteobacteria;o__Burkholderiales;f__Comamonadaceae;g__Variovorax;s__Variovorax boronicumulans NBRC 103145 |
| HAAB01020508.1.1261 | -0.81856 | 0 | I | 1 | 0.0585% | peripheral-nodes | Moderate | d__Bacteria;p__Proteobacteria;c__Betaproteobacteria;o__Burkholderiales;f__Oxalobacteraceae;g__Massilia;s__Triticum aestivum (bread wheat) |
| HACI01281742.47.1472 | -0.76423 | 0 | I | 2 | 0.0586% | peripheral-nodes | Moderate | d__Bacteria;p__Proteobacteria;c__Gammaproteobacteria;o__Pseudomonadales;f__Pseudomonadaceae;g__Pseudomonas;s__Pogona vitticeps (central bearded dragon) |
| KR422351.1.1495 | -0.65558 | 0 | IV | 4 | 0.0599% | peripheral-nodes | Moderate | d__Bacteria;p__Firmicutes;c__Erysipelotrichia;o__Erysipelotrichales;f__Erysipelotrichaceae;g__Turicibacter;s__Turicibacter sanguinis |
| LRAC01000105.2.1555 | -0.05802 | 0.221453 | I | 17 | 0.0608% | peripheral-nodes | Moderate | d__Bacteria;p__Firmicutes;c__Bacilli;o__Bacillales;f__Paenibacillaceae;g__Paenibacillus;s__Paenibacillus sp. DMB5 |
| JN210898.1.1382 | -0.43829 | 0.36 | IV | 10 | 0.0613% | peripheral-nodes | Moderate | d__Bacteria;p__Proteobacteria;c__Alphaproteobacteria;o__Rhizobiales;f__Phyllobacteriaceae;g__Aliihoeflea;s__Aliihoeflea sp. S9 |
| KF843057.1.1395 | -0.56549 | 0.998889 | II | 60 | 0.0617% | connectors | Moderate | d__Bacteria;p__Proteobacteria;c__Gammaproteobacteria;o__Enterobacteriales;f__Enterobacteriaceae;g__Escherichia-Shigella;s__uncultured bacterium |
| JSYO01000045.9.1526 | -0.56549 | 0.999228 | I | 72 | 0.0627% | connectors | Moderate | d__Bacteria;p__Bacteroidetes;c__Flavobacteriia;o__Flavobacteriales;f__Flavobacteriaceae;g__Flavobacterium;s__Flavobacterium sp. JRM |
| BADC01000707.6291.7794 | 0.756843 | 0.305556 | I | 36 | 0.0630% | peripheral-nodes | Moderate | d__Bacteria;p__Actinobacteria;c__Actinobacteria;o__Micrococcales;f__Microbacteriaceae;g__Herbiconiux;s__Corynebacterium-like bacterium B27 |
| AB539906.1.1320 | -0.76423 | 0 | VI | 2 | 0.0649% | peripheral-nodes | Moderate | d__Bacteria;p__Firmicutes;c__Clostridia;o__Clostridiales;f__Clostridiaceae 1;g__Clostridium sensu stricto 1;s__Clostridium sp. C5S17 |
| BAZJ01000111.3785.5292 | -0.81856 | 0 | I | 1 | 0.0666% | peripheral-nodes | Moderate | d__Bacteria;p__Proteobacteria;c__Betaproteobacteria;o__Burkholderiales;f__Comamonadaceae;g__Diaphorobacter;s__Diaphorobacter nitroreducens JCM 11421 |
| EF111232.1.1272 | -0.11234 | 0.128889 | I | 15 | 0.0676% | peripheral-nodes | Moderate | d__Bacteria;p__Bacteroidetes;c__Sphingobacteriia;o__Sphingobacteriales;f__Sphingobacteriaceae;g__Sphingobacterium;s__Bacteroidetes bacterium RBE2CD-54 |
| DQ129408.1.1279 | -0.65558 | 0 | VI | 4 | 0.0679% | peripheral-nodes | Moderate | d__Bacteria;p__Firmicutes;c__Erysipelotrichia;o__Erysipelotrichales;f__Erysipelotrichaceae;g__Turicibacter;s__uncultured bacterium |
| CCNK01000040.27609.29128 | -0.81856 | 0 | Other | 1 | 0.0694% | peripheral-nodes | Moderate | d__Bacteria;p__Proteobacteria;c__Gammaproteobacteria;o__Pasteurellales;f__Pasteurellaceae;g__norank;s__Haemophilus sp. FF7 |
| KT952763.1.1479 | -0.11234 | 0.395062 | V | 18 | 0.0697% | peripheral-nodes | Moderate | d__Bacteria;p__Firmicutes;c__Clostridia;o__Clostridiales;f__Clostridiaceae 1;g__Clostridium sensu stricto 1;s__uncultured bacterium |
| HG800055.1.1454 | -0.81856 | 0 | Other | 1 | 0.0713% | peripheral-nodes | Moderate | d__Bacteria;p__Proteobacteria;c__Gammaproteobacteria;o__Xanthomonadales;f__Xanthomonadaceae;g__Stenotrophomonas;s__bacterium BII-R7 |
| HG917264.1.1510 | -0.54694 | 0 | VI | 6 | 0.0717% | peripheral-nodes | Moderate | d__Bacteria;p__Proteobacteria;c__Gammaproteobacteria;o__Pseudomonadales;f__Pseudomonadaceae;g__Pseudomonas;s__uncultured Bacilli bacterium |
| LKIE01000078.65.1536 | -0.81856 | 0 | VII | 1 | 0.0757% | peripheral-nodes | Moderate | d__Bacteria;p__Firmicutes;c__Bacilli;o__Bacillales;f__Planococcaceae;g__Lysinibacillus;s__Lysinibacillus sp. F5 |
| AY465361.1.1362 | -0.81856 | 0 | Other | 1 | 0.0771% | peripheral-nodes | Moderate | d__Bacteria;p__Proteobacteria;c__Gammaproteobacteria;o__Pasteurellales;f__Pasteurellaceae;g__Actinobacillus;s__Bisgaard Taxon 10 |
| ACOM01000010.1834.3339 | -0.81856 | 0 | Other | 1 | 0.0861% | peripheral-nodes | Moderate | d__Bacteria;p__Firmicutes;c__Clostridia;o__Clostridiales;f__Clostridiaceae 1;g__Clostridium sensu stricto 1;s__Clostridium butyricum E4 str. BoNT E BL5262 |
| AB020206.1.1443 | 1.89765 | 0.199446 | I | 57 | 0.0876% | peripheral-nodes | Moderate | d__Bacteria;p__Bacteroidetes;c__Sphingobacteriia;o__Sphingobacteriales;f__Sphingobacteriaceae;g__Pedobacter;s__Sphingobacterium sp. OM-E81 |
| JNIX01000007.103331.104771 | 1.89765 | 0.199446 | I | 57 | 0.0921% | peripheral-nodes | Moderate | d__Bacteria;p__Proteobacteria;c__Alphaproteobacteria;o__Caulobacterales;f__Caulobacteraceae;g__Brevundimonas;s__Brevundimonas bacteroides DSM 4726 |
| JRMB01000002.1341.2886 | 1.626029 | 0.186467 | I | 51 | 0.0931% | peripheral-nodes | Moderate | d__Bacteria;p__Proteobacteria;c__Gammaproteobacteria;o__Pseudomonadales;f__Pseudomonadaceae;g__Pseudomonas;s__Pseudomonas lutea |
| GQ099298.1.1364 | 0.050629 | 0.2775 | I | 20 | 0.0999% | peripheral-nodes | Moderate | d__Bacteria;p__Proteobacteria;c__Gammaproteobacteria;o__Enterobacteriales;f__Enterobacteriaceae;g__Serratia;s__uncultured bacterium |
| FJ849472.1.1466 | -0.76423 | 0 | VI | 2 | 0.1008% | peripheral-nodes | Abundant | d__Bacteria;p__Proteobacteria;c__Gammaproteobacteria;o__Enterobacteriales;f__Enterobacteriaceae;g__Serratia;s__uncultured bacterium |
| JANU01000013.125494.127008 | 2.060622 | 0.16231 | I | 59 | 0.1016% | peripheral-nodes | Moderate | d__Bacteria;p__Proteobacteria;c__Betaproteobacteria;o__Burkholderiales;f__Comamonadaceae;g__Caenimonas;s__Caenimonas sp. SL110 |
| JUZO01000179.291.1810 | -0.65555 | 1 | I | 5 | 0.1105% | connectors | Moderate | d__Bacteria;p__Proteobacteria;c__Gammaproteobacteria;o__Enterobacteriales;f__Enterobacteriaceae;g__Raoultella;s__Salmonella enterica |
| CBXX010000006.258445.259957 | 2.929808 | 0.151662 | I | 76 | 0.1116% | module-hubs | Moderate | d__Bacteria;p__Proteobacteria;c__Betaproteobacteria;o__Burkholderiales;f__Oxalobacteraceae;g__Herbaspirillum;s__Herbaspirillum sp. RV1423 |
| LEKS01000043.570.2057 | 2.549539 | 0.19 | I | 70 | 0.1123% | module-hubs | Moderate | d__Bacteria;p__Bacteroidetes;c__Flavobacteriia;o__Flavobacteriales;f__Flavobacteriaceae;g__Flavobacterium;s__Flavobacterium sp. ABG |
| FJ678991.1.1426 | -0.70991 | 0 | VII | 3 | 0.1125% | peripheral-nodes | Moderate | d__Bacteria;p__Firmicutes;c__Clostridia;o__Clostridiales;f__Clostridiaceae 1;g__Clostridium sensu stricto 1;s__uncultured bacterium |
| KF103466.1.1359 | 0.104953 | 0.102493 | I | 19 | 0.1161% | peripheral-nodes | Moderate | d__Bacteria;p__Proteobacteria;c__Gammaproteobacteria;o__Enterobacteriales;f__Enterobacteriaceae;g__Enterobacter;s__uncultured bacterium |
| GBGQ01006232.25.1541 | -0.76423 | 0 | I | 2 | 0.1162% | peripheral-nodes | Moderate | d__Bacteria;p__Proteobacteria;c__Gammaproteobacteria;o__Pseudomonadales;f__Pseudomonadaceae;g__Pseudomonas;s__Schmidtea mediterranea |
| ARMC01000030.225697.227205 | -2.04669 | 0.999731 | I | 61 | 0.1170% | connectors | Moderate | d__Bacteria;p__Proteobacteria;c__Betaproteobacteria;o__Burkholderiales;f__Oxalobacteraceae;g__Duganella;s__Oxalobacteraceae bacterium AB_14 |
| JUEU01000001.55214.56761 | 2.16927 | 0.157216 | I | 61 | 0.1192% | peripheral-nodes | Moderate | d__Bacteria;p__Proteobacteria;c__Gammaproteobacteria;o__Pseudomonadales;f__Pseudomonadaceae;g__Pseudomonas;s__Pseudomonas coronafaciens pv. porri |
| JRLF01000016.33782.35290 | -0.43829 | 0.471074 | I | 11 | 0.1285% | peripheral-nodes | Moderate | d__Bacteria;p__Bacteroidetes;c__Flavobacteriia;o__Flavobacteriales;f__Flavobacteriaceae;g__Flavobacterium;s__Flavobacterium aquidurense |
| GQ263283.1.1438 | 0.430898 | 0.0784 | IV | 25 | 0.1288% | peripheral-nodes | Moderate | d__Bacteria;p__Proteobacteria;c__Alphaproteobacteria;o__Rhizobiales;f__Hyphomicrobiaceae;g__Pelagibacterium;s__uncultured bacterium |
| ACPC01000001.16975.18506 | -0.76423 | 0 | VII | 2 | 0.1419% | peripheral-nodes | Moderate | d__Bacteria;p__Firmicutes;c__Bacilli;o__Bacillales;f__Bacillaceae;g__Bacillus;s__Bacillus sp. m3-13 |
| FM865640.1.1411 | 2.495215 | 0.192607 | I | 69 | 0.1492% | peripheral-nodes | Moderate | d__Bacteria;p__Bacteroidetes;c__Sphingobacteriia;o__Sphingobacteriales;f__Sphingobacteriaceae;g__Pedobacter;s__uncultured Pedobacter sp. |
| BAGY01000082.170.1698 | 0.104953 | 0 | I | 18 | 0.1595% | peripheral-nodes | Moderate | d__Bacteria;p__Proteobacteria;c__Gammaproteobacteria;o__Pseudomonadales;f__Moraxellaceae;g__Acinetobacter;s__Acinetobacter radioresistens DSM 6976 = NBRC 102413 = CIP 103788 |
| HE582440.1.1499 | 1.789002 | 0.176612 | II | 54 | 0.1613% | peripheral-nodes | Moderate | d__Bacteria;p__Proteobacteria;c__Gammaproteobacteria;o__Enterobacteriales;f__Enterobacteriaceae;g__Escherichia-Shigella;s__uncultured bacterium |
| KU353559.1.1392 | -0.49261 | 0 | VI | 7 | 0.1617% | peripheral-nodes | Moderate | d__Bacteria;p__Proteobacteria;c__Gammaproteobacteria;o__Pseudomonadales;f__Moraxellaceae;g__Acinetobacter;s__Acinetobacter sp. YC6 |
| CENA01000012.265.1757 | -0.64784 | 1 | IV | 3 | 0.1738% | connectors | Moderate | d__Bacteria;p__Firmicutes;c__Clostridia;o__Clostridiales;f__Peptostreptococcaceae;g__Terrisporobacter;s__Paeniclostridium sordellii |
| AVSV01000024.80.1573 | -0.81856 | 0 | Other | 1 | 0.1801% | peripheral-nodes | Moderate | d__Bacteria;p__Firmicutes;c__Clostridia;o__Clostridiales;f__Clostridiaceae 1;g__Clostridium sensu stricto 1;s__Clostridium sp. Ade.TY |
| ASZT02000282.376.1913 | -0.65558 | 0.36 | I | 5 | 0.1880% | peripheral-nodes | Moderate | d__Bacteria;p__Bacteroidetes;c__Sphingobacteriia;o__Sphingobacteriales;f__Sphingobacteriaceae;g__Sphingobacterium;s__Sphingobacterium sp. IITKGP-BTPF85 |
| JQ691692.1.1488 | -0.60126 | 0 | VI | 5 | 0.1906% | peripheral-nodes | Moderate | d__Bacteria;p__Proteobacteria;c__Gammaproteobacteria;o__Pseudomonadales;f__Pseudomonadaceae;g__Pseudomonas;s__Pseudomonas fluorescens |
| AEAZ01000049.71.1611 | 1.137112 | 0.223923 | I | 42 | 0.1916% | peripheral-nodes | Moderate | d__Bacteria;p__Proteobacteria;c__Gammaproteobacteria;o__Pseudomonadales;f__Pseudomonadaceae;g__Pseudomonas;s__Pseudomonas fluorescens WH6 |
| JATV01000001.327279.328772 | 1.951974 | 0.223212 | I | 59 | 0.2101% | peripheral-nodes | Moderate | d__Bacteria;p__Bacteroidetes;c__Flavobacteriia;o__Flavobacteriales;f__Flavobacteriaceae;g__Flavobacterium;s__Flavobacterium succinicans LMG 10402 |
| AB175372.1.1452 | 0.865491 | 0.209877 | I | 36 | 0.2295% | peripheral-nodes | Moderate | d__Bacteria;p__Proteobacteria;c__Betaproteobacteria;o__Burkholderiales;f__Comamonadaceae;g__Comamonas;s__uncultured bacterium |
| DQ816532.1.1431 | -1.19338 | 1 | V | 14 | 0.2418% | connectors | Moderate | d__Bacteria;p__Firmicutes;c__Clostridia;o__Clostridiales;f__Clostridiaceae 1;g__Clostridium sensu stricto 1;s__uncultured bacterium |
| JXRA01000009.689.2208 | 0.648195 | 0.234375 | I | 32 | 0.2809% | peripheral-nodes | Moderate | d__Bacteria;p__Bacteroidetes;c__Sphingobacteriia;o__Sphingobacteriales;f__Sphingobacteriaceae;g__Pedobacter;s__Pedobacter sp. NL19 |
| HM627388.1.1294 | 1.408733 | 0.234375 | I | 48 | 0.2837% | peripheral-nodes | Moderate | d__Bacteria;p__Proteobacteria;c__Gammaproteobacteria;o__Enterobacteriales;f__Enterobacteriaceae;g__Raoultella;s__Raoultella sp. 49 |
| AOIF01000172.30.1396 | -0.76423 | 0 | Other | 2 | 0.3410% | peripheral-nodes | Moderate | d__Bacteria;p__Firmicutes;c__Clostridia;o__Clostridiales;f__Clostridiaceae 1;g__Clostridium sensu stricto 1;s__Clostridium saccharoperbutylacetonicum N1-4(HMT) |
| GQ868411.1.1429 | -0.65555 | 1 | V | 2 | 0.3464% | connectors | Moderate | d__Bacteria;p__Firmicutes;c__Clostridia;o__Clostridiales;f__Peptostreptococcaceae;g__Terrisporobacter;s__uncultured Clostridium sp. |
| DQ145940.1.1404 | 0.213602 | 0.305556 | I | 24 | 0.3930% | peripheral-nodes | Moderate | d__Bacteria;p__Bacteroidetes;c__Sphingobacteriia;o__Sphingobacteriales;f__Sphingobacteriaceae;g__Pedobacter;s__Pedobacter sp. TSBY-14 |
| ATZS02000073.1.1275 | 2.277919 | 0.178711 | I | 64 | 0.3973% | peripheral-nodes | Moderate | d__Bacteria;p__Proteobacteria;c__Betaproteobacteria;o__Burkholderiales;f__Oxalobacteraceae;g__Duganella;s__beta proteobacterium SCGC AAA027-I06 |
| CYZR01000022.1365.2854 | -0.81856 | 0 | Other | 1 | 0.4245% | peripheral-nodes | Moderate | d__Bacteria;p__Firmicutes;c__Clostridia;o__Clostridiales;f__Clostridiaceae 1;g__Sarcina;s__Clostridium ventriculi |
| GAMA01019719.1.1503 | -0.81856 | 0.75 | I | 2 | 0.4463% | connectors | Moderate | d__Bacteria;p__Proteobacteria;c__Gammaproteobacteria;o__Enterobacteriales;f__Enterobacteriaceae;g__Rosenbergiella;s__Actinidia chinensis |
| AF212202.1.1454 | 0.267926 | 0.166352 | IV | 23 | 0.4538% | peripheral-nodes | Moderate | d__Bacteria;p__Proteobacteria;c__Gammaproteobacteria;o__Oceanospirillales;f__Halomonadaceae;g__Halomonas;s__Halomonas neptunia |
| EU071514.1.1480 | -0.76423 | 0.75 | V | 4 | 0.4993% | connectors | Moderate | d__Bacteria;p__Firmicutes;c__Erysipelotrichia;o__Erysipelotrichales;f__Erysipelotrichaceae;g__Turicibacter;s__uncultured Firmicutes bacterium |
| LBCO01000023.28.1555 | 2.332243 | 0.176095 | I | 65 | 0.5539% | peripheral-nodes | Moderate | d__Bacteria;p__Proteobacteria;c__Betaproteobacteria;o__Burkholderiales;f__Oxalobacteraceae;g__Massilia;s__Janthinobacterium sp. KBS0711 |
| BAAW01000377.208.1702 | -0.81856 | 0 | Other | 1 | 0.5970% | peripheral-nodes | Moderate | d__Bacteria;p__Firmicutes;c__Clostridia;o__Clostridiales;f__Clostridiaceae 1;g__Clostridium sensu stricto 1;s__human gut metagenome |
| HQ739847.1.1448 | -0.16667 | 0.137755 | IV | 14 | 0.5975% | peripheral-nodes | Moderate | d__Bacteria;p__Proteobacteria;c__Gammaproteobacteria;o__Enterobacteriales;f__Enterobacteriaceae;g__Escherichia-Shigella;s__uncultured bacterium |
| BAWX01000122.24.1513 | -2.09872 | 1 | IV | 23 | 0.6089% | connectors | Moderate | d__Bacteria;p__Proteobacteria;c__Gammaproteobacteria;o__Oceanospirillales;f__Halomonadaceae;g__Halomonas;s__Halomonas sp. JCM 19031 |
| JXQR01000012.10.1527 | 2.984132 | 0.192277 | I | 79 | 0.6366% | module-hubs | Moderate | d__Bacteria;p__Bacteroidetes;c__Flavobacteriia;o__Flavobacteriales;f__Flavobacteriaceae;g__Flavobacterium;s__Flavobacterium sp. MEB061 |
| GBYS01143968.8667.10121 | -0.76423 | 0 | Other | 2 | 0.6931% | peripheral-nodes | Moderate | d__Bacteria;p__Cyanobacteria;c__Chloroplast;o__norank;f__norank;g__norank;s__Oryza meyeriana |
| AB812750.1.1545 | -0.76423 | 0.555556 | VIII | 3 | 0.7073% | peripheral-nodes | Moderate | d__Bacteria;p__Firmicutes;c__Bacilli;o__Lactobacillales;f__Lactobacillaceae;g__Lactobacillus;s__Lactobacillus faecis |
| ACLL01000037.71.1634 | -0.8765 | 1 | VIII | 13 | 0.8205% | connectors | Moderate | d__Bacteria;p__Firmicutes;c__Bacilli;o__Lactobacillales;f__Lactobacillaceae;g__Lactobacillus;s__Lactobacillus antri DSM 16041 |
| GAIQ01048712.3375.4897 | -0.81856 | 0 | IV | 1 | 0.9196% | peripheral-nodes | Moderate | d__Bacteria;p__Proteobacteria;c__Gammaproteobacteria;o__Enterobacteriales;f__Enterobacteriaceae;g__Escherichia-Shigella;s__Alexandrium tamarense |
| GAQJ01015982.54.1465 | -0.60126 | 0 | I | 5 | 1.1326% | peripheral-nodes | Moderate | d__Bacteria;p__Proteobacteria;c__Gammaproteobacteria;o__Pseudomonadales;f__Pseudomonadaceae;g__Pseudomonas;s__Ostrinia furnacalis (Asian corn borer) |
| GEGG01003054.119.1620 | 2.332243 | 0.200872 | I | 66 | 1.1858% | peripheral-nodes | Moderate | d__Bacteria;p__Actinobacteria;c__Actinobacteria;o__Streptomycetales;f__Streptomycetaceae;g__E1B-B3-114;s__Rhacophorus dennysi |
| AICL01000003.170338.171909 | -0.70991 | 0.4375 | VIII | 4 | 1.5517% | peripheral-nodes | Moderate | d__Bacteria;p__Firmicutes;c__Bacilli;o__Lactobacillales;f__Lactobacillaceae;g__Lactobacillus;s__Lactobacillus salivarius SMXD51 |
| JROC01000033.4711.6372 | -0.60126 | 0.305556 | VIII | 6 | 2.2889% | peripheral-nodes | Moderate | d__Bacteria;p__Firmicutes;c__Bacilli;o__Lactobacillales;f__Lactobacillaceae;g__Lactobacillus;s__Lactobacillus mucosae |
| LARB01000143.417.1794 | -0.49261 | 0.234375 | IV | 8 | 3.1582% | peripheral-nodes | Moderate | d__Bacteria;p__Firmicutes;c__Clostridia;o__Clostridiales;f__Clostridiaceae 1;g__Clostridium sensu stricto 1;s__Ruminococcus sp. A254.MGS-254 |
| ACGR01000047.118.1678 | -0.16667 | 0.137755 | VIII | 14 | 4.8199% | peripheral-nodes | Moderate | d__Bacteria;p__Firmicutes;c__Bacilli;o__Lactobacillales;f__Lactobacillaceae;g__Lactobacillus;s__Lactobacillus johnsonii ATCC 33200 |
| CBXK010001759.1485.3013 | -0.81856 | 0 | II | 1 | 5.4190% | peripheral-nodes | Moderate | d__Bacteria;p__Firmicutes;c__Bacilli;o__Lactobacillales;f__Streptococcaceae;g__Streptococcus;s__Trichuris trichiura (human whipworm) |
| GBHB01021588.2.1499 | 0.267926 | 0.088843 | I | 22 | 6.7432% | peripheral-nodes | Moderate | d__Bacteria;p__Proteobacteria;c__Gammaproteobacteria;o__Pseudomonadales;f__Pseudomonadaceae;g__Pseudomonas;s__Teleogryllus commodus |
| AMEZ01000038.1.1425 | -0.38396 | 0.19 | V | 10 | 11.0771% | peripheral-nodes | Moderate | d__Bacteria;p__Firmicutes;c__Clostridia;o__Clostridiales;f__Clostridiaceae 1;g__Clostridium sensu stricto 1;s__Clostridium celatum DSM 1785 |
| GADT01088274.11.1516 | 3.907643 | 0.123585 | II | 94 | 20.7762% | module-hubs | Moderate | d__Bacteria;p__Proteobacteria;c__Gammaproteobacteria;o__Enterobacteriales;f__Enterobacteriaceae;g__Escherichia-Shigella;s__Camelus dromedarius (Arabian camel) |

#### Table. S4

**Table. S4** Dissimilarity index of microbial network among three giant panda populations (GPCAP, GPMS, and GPXXL).

| **Associated networks** | **Dissimilarity index** |
| --- | --- |
| GPCAP -- GPMS | 0.999514504 |
| GPCAP -- GPXXL | 0.985812409 |
| GPXXL -- GPMS | 0.873962555 |

#### Table. S5

**Table. S5** Information on keystone species in giant pandas.

| **Groups** | **OTU-ID** | **Degree** | **Betweenness** | **Abundance** | **Relative-abundance** | **Abundance classification** | **Taxonomy** |
| --- | --- | --- | --- | --- | --- | --- | --- |
| GP | HM295535.1.1352 | 52 | 2693.66 | 185 | 0.0044% | Rare | d__Bacteria; p__Proteobacteria; c__Betaproteobacteria; o__Burkholderiales; f__Oxalobacteraceae; g__Paucimonas; s__uncultured bacterium |
|  | AY468453.1.1450 | 66 | 3991.16 | 341 | 0.0100% | Moderate | d__Bacteria; p__Bacteroidetes; c__Flavobacteriia; o__Flavobacteriales; f__Flavobacteriaceae; g__Flavobacterium; s__cf. Chryseobacterium sp. UOF CM895 |
|  | KM035944.1.1434 | 66 | 3111.60 | 372 | 0.0100% | Moderate | d__Bacteria; p__Bacteroidetes; c__Sphingobacteriia; o__Sphingobacteriales; f__Sphingobacteriaceae; g__Pedobacter; s__Pedobacter trunci |
|  | JROH01000090.372.1843 | 61 | 3580.29 | 439 | 0.0100% | Moderate | d__Bacteria; p__Proteobacteria; c__Alphaproteobacteria; o__Sphingomonadales; f__Sphingomonadaceae; g__Sphingomonas; s__Sphingomonas sp. 37zxx |
|  | JRLX01000051.261.1786 | 52 | 397.40 | 345 | 0.0100% | Moderate | d__Bacteria; p__Bacteroidetes; c__Flavobacteriia; o__Flavobacteriales; f__Flavobacteriaceae; g__Flavobacterium; s__Flavobacterium rivuli WB 3.3-2 = DSM 21788 |
|  | KF077988.1.1363 | 51 | 1423.13 | 504 | 0.0100% | Moderate | d__Bacteria; p__Proteobacteria; c__Gammaproteobacteria; o__Enterobacteriales; f__Enterobacteriaceae; g__Serratia; s__uncultured bacterium |
|  | AYZX01097562.161.1662 | 66 | 2059.27 | 838 | 0.0200% | Moderate | d__Bacteria; p__Bacteroidetes; c__Sphingobacteriia; o__Sphingobacteriales; f__Sphingobacteriaceae; g__Pedobacter; s__Beta vulgaris subsp. vulgaris |
|  | AYXT01000013.423283.424892 | 63 | 3607.68 | 667 | 0.0200% | Moderate | d__Bacteria; p__Proteobacteria; c__Betaproteobacteria; o__Burkholderiales; f__Alcaligenaceae; g__Advenella; s__Advenella kashmirensis W13003 |
|  | AEZJ02000039.521.2062 | 58 | 96.40 | 730 | 0.0200% | Moderate | d__Bacteria; p__Proteobacteria; c__Gammaproteobacteria; o__Enterobacteriales; f__Enterobacteriaceae; g__Escherichia-Shigella; s__Escherichia coli 97.0246 |
|  | JNFF01000078.74.1510 | 56 | 1738.47 | 697 | 0.0200% | Moderate | d__Bacteria; p__Bacteroidetes; c__Sphingobacteriia; o__Sphingobacteriales; f__Sphingobacteriaceae; g__Pedobacter; s__Pedobacter antarcticus 4BY |
|  | HM284700.1.1364 | 68 | 487.97 | 1173 | 0.0300% | Moderate | d__Bacteria; p__Proteobacteria; c__Gammaproteobacteria; o__Enterobacteriales; f__Enterobacteriaceae; g__Serratia; s__uncultured bacterium |
|  | HQ758829.1.1473 | 61 | 313.27 | 1809 | 0.0400% | Moderate | d__Bacteria; p__Proteobacteria; c__Gammaproteobacteria; o__Enterobacteriales; f__Enterobacteriaceae; g__Escherichia-Shigella; s__uncultured organism |
|  | JX029047.1.1223 | 58 | 231.64 | 1642 | 0.0400% | Moderate | d__Bacteria; p__Proteobacteria; c__Gammaproteobacteria; o__Enterobacteriales; f__Enterobacteriaceae; g__Escherichia-Shigella; s__Pectobacterium carotovorum subsp. carotovorum |
|  | LMPU01000009.953875.955405 | 58 | 1062.56 | 1580 | 0.0400% | Moderate | d__Bacteria; p__Bacteroidetes; c__Sphingobacteriia; o__Sphingobacteriales; f__Sphingobacteriaceae; g__Pedobacter; s__Pedobacter sp. Leaf194 |
|  | JUGK01000006.967.2463 | 54 | 2844.26 | 1602 | 0.0400% | Moderate | d__Bacteria; p__Bacteroidetes; c__Flavobacteriia; o__Flavobacteriales; f__Flavobacteriaceae; g__Chryseobacterium; s__Chryseobacterium sp. YR005 |
|  | AB020206.1.1443 | 58 | 3119.45 | 3691 | 0.0900% | Moderate | d__Bacteria; p__Bacteroidetes; c__Sphingobacteriia; o__Sphingobacteriales; f__Sphingobacteriaceae; g__Pedobacter; s__Sphingobacterium sp. OM-E81 |
|  | JNIX01000007.103331.104771 | 57 | 1356.99 | 3882 | 0.0900% | Moderate | d__Bacteria; p__Proteobacteria; c__Alphaproteobacteria; o__Caulobacterales; f__Caulobacteraceae; g__Brevundimonas; s__Brevundimonas bacteroides DSM 4726 |
|  | JANU01000013.125494.127008 | 59 | 3192.17 | 4279 | 0.1000% | Moderate | d__Bacteria; p__Proteobacteria; c__Betaproteobacteria; o__Burkholderiales; f__Comamonadaceae; g__Caenimonas; s__Caenimonas sp. SL110 |
|  | JATV01000001.327279.328772 | 59 | 1857.16 | 8853 | 0.2100% | Abundant | d__Bacteria; p__Bacteroidetes; c__Flavobacteriia; o__Flavobacteriales; f__Flavobacteriaceae; g__Flavobacterium; s__Flavobacterium succinicans LMG 10402 |
| GPMS | AJ288899.1.1371 | 60 | 4520.16 | 182 | 0.0153% | Moderate | d__Bacteria; p__Proteobacteria; c__Deltaproteobacteria; o__Bdellovibrionales; f__Bacteriovoracaceae; g__Bacteriovorax; s__Bacteriovorax stolpii |
|  | KM035944.1.1434 | 53 | 3489.24 | 323 | 0.0272% | Moderate | d__Bacteria; p__Bacteroidetes; c__Sphingobacteriia; o__Sphingobacteriales; f__Sphingobacteriaceae; g__Pedobacter; s__Pedobacter trunci |
|  | FJ444768.1.1511 | 54 | 3432.97 | 379 | 0.0319% | Moderate | d__Bacteria; p__Verrucomicrobia; c__Opitutae; o__Opitutales; f__Opitutaceae; g__Opitutus; s__uncultured bacterium |
|  | JUHE01000001.1123234.1124752 | 59 | 3654.84 | 390 | 0.0328% | Moderate | d__Bacteria; p__Proteobacteria; c__Betaproteobacteria; o__Methylophilales; f__Methylophilaceae; g__Methylophilus; s__Methylophilus sp. Q8 |
|  | KC189756.1.1450 | 51 | 4195.53 | 396 | 0.0333% | Moderate | d__Bacteria; p__Proteobacteria; c__Alphaproteobacteria; o__Alphaproteobacteria Incertae Sedis; f__uncultured; g__norank; s__uncultured bacterium |
|  | JROH01000090.372.1843 | 60 | 4930.82 | 428 | 0.0360% | Moderate | d__Bacteria; p__Proteobacteria; c__Alphaproteobacteria; o__Sphingomonadales; f__Sphingomonadaceae; g__Sphingomonas; s__Sphingomonas sp. 37zxx |
|  | JAUG01000180.350.1851 | 61 | 2104.75 | 530 | 0.0446% | Moderate | d__Bacteria; p__Bacteroidetes; c__Sphingobacteriia; o__Sphingobacteriales; f__Sphingobacteriaceae; g__Pedobacter; s__Pedobacter borealis DSM 19626 |
|  | JNFF01000078.74.1510 | 55 | 1106.92 | 579 | 0.0487% | Moderate | d__Bacteria; p__Bacteroidetes; c__Sphingobacteriia; o__Sphingobacteriales; f__Sphingobacteriaceae; g__Pedobacter; s__Pedobacter antarcticus 4BY |
|  | JQ655794.1.1496 | 63 | 3455.26 | 758 | 0.0638% | Moderate | d__Bacteria; p__Verrucomicrobia; c__Opitutae; o__Opitutae vadinHA64; f__norank; g__norank; s__uncultured bacterium |
|  | KF088459.1.1352 | 53 | 1105.67 | 936 | 0.0788% | Moderate | d__Bacteria; p__Proteobacteria; c__Betaproteobacteria; o__Burkholderiales; f__Comamonadaceae; g__Acidovorax; s__uncultured bacterium |
|  | LMPU01000009.953875.955405 | 75 | 3770.57 | 1205 | 0.1014% | Abundant | d__Bacteria; p__Bacteroidetes; c__Sphingobacteriia; o__Sphingobacteriales; f__Sphingobacteriaceae; g__Pedobacter; s__Pedobacter sp. Leaf194 |
|  | LCTB01000191.2.1381 | 60 | 3309.74 | 1303 | 0.1097% | Abundant | d__Bacteria; p__Proteobacteria; c__Betaproteobacteria; o__Burkholderiales; f__Comamonadaceae; g__Pseudorhodoferax; s__Pseudomonas aeruginosa |
|  | CBXX010000006.258445.259957 | 62 | 2598.83 | 4604 | 0.3875% | Abundant | d__Bacteria; p__Proteobacteria; c__Betaproteobacteria; o__Burkholderiales; f__Oxalobacteraceae; g__Herbaspirillum; s__Herbaspirillum sp. RV1423 |
|  | ARMC01000030.225697.227205 | 57 | 1574.05 | 4775 | 0.4019% | Abundant | d__Bacteria; p__Proteobacteria; c__Betaproteobacteria; o__Burkholderiales; f__Oxalobacteraceae; g__Duganella; s__Oxalobacteraceae bacterium AB_14 |
| GPXXL | EU358719.1.1513 | 51 | 174.87 | 30 | 0.0024% | Rare | d__Bacteria; p__Firmicutes; c__Bacilli; o__Lactobacillales; f__Streptococcaceae; g__Streptococcus; s__uncultured bacterium |
|  | CBYE010000031.3297.4803 | 70 | 328.81 | 32 | 0.0025% | Rare | d__Bacteria; p__Bacteroidetes; c__Flavobacteriia; o__Flavobacteriales; f__Flavobacteriaceae; g__Elizabethkingia; s__Elizabethkingia anophelis PW2809 |
|  | KC918346.1.1295 | 62 | 253.84 | 38 | 0.0030% | Rare | d__Bacteria; p__Proteobacteria; c__Gammaproteobacteria; o__Aeromonadales; f__Aeromonadaceae; g__Aeromonas; s__uncultured Aeromonas sp. |
|  | FJ670960.1.1402 | 55 | 170.00 | 42 | 0.0033% | Rare | d__Bacteria; p__Firmicutes; c__Clostridia; o__Clostridiales; f__Clostridiaceae 1; g__Clostridium sensu stricto 1; s__uncultured bacterium |
|  | HQ800197.1.1426 | 57 | 376.34 | 42 | 0.0033% | Rare | d__Bacteria; p__Firmicutes; c__Clostridia; o__Clostridiales; f__Clostridiaceae 1; g__Clostridium sensu stricto 1; s__uncultured organism |
|  | LC125154.1.1442 | 53 | 279.45 | 42 | 0.0033% | Rare | d__Bacteria; p__Proteobacteria; c__Gammaproteobacteria; o__Pseudomonadales; f__Pseudomonadaceae; g__Pseudomonas; s__Pseudomonas sp. Oh-1.0.2 |
|  | HQ809662.1.1427 | 62 | 199.18 | 44 | 0.0035% | Rare | d__Bacteria; p__Firmicutes; c__Clostridia; o__Clostridiales; f__Clostridiaceae 1; g__Clostridium sensu stricto 1; s__uncultured organism |
|  | HQ741332.1.1445 | 76 | 623.56 | 45 | 0.0036% | Rare | d__Bacteria; p__Firmicutes; c__Clostridia; o__Clostridiales; f__Clostridiaceae 1; g__Clostridium sensu stricto 1; s__uncultured bacterium |
|  | JQ453607.1.1387 | 52 | 127.11 | 48 | 0.0038% | Rare | d__Bacteria; p__Firmicutes; c__Bacilli; o__Lactobacillales; f__Streptococcaceae; g__Streptococcus; s__uncultured bacterium |
|  | EU775669.1.1323 | 73 | 465.70 | 49 | 0.0039% | Rare | d__Bacteria; p__Firmicutes; c__Clostridia; o__Clostridiales; f__Clostridiaceae 1; g__Clostridium sensu stricto 1; s__uncultured bacterium |
|  | GQ868409.1.1439 | 80 | 571.90 | 53 | 0.0042% | Rare | d__Bacteria; p__Firmicutes; c__Clostridia; o__Clostridiales; f__Clostridiaceae 1; g__Clostridium sensu stricto 1; s__uncultured Clostridium sp. |
|  | HQ741385.1.1426 | 59 | 429.57 | 58 | 0.0046% | Rare | d__Bacteria; p__Firmicutes; c__Clostridia; o__Clostridiales; f__Clostridiaceae 1; g__Clostridium sensu stricto 1; s__uncultured bacterium |
|  | HQ741239.1.1415 | 61 | 195.06 | 63 | 0.0050% | Rare | d__Bacteria; p__Firmicutes; c__Clostridia; o__Clostridiales; f__Clostridiaceae 1; g__Clostridium sensu stricto 1; s__uncultured bacterium |
|  | EF401512.1.1477 | 60 | 409.50 | 66 | 0.0053% | Rare | d__Bacteria; p__Firmicutes; c__Clostridia; o__Clostridiales; f__Clostridiaceae 1; g__Clostridium sensu stricto 1; s__uncultured bacterium |
|  | EU459317.1.1373 | 79 | 721.69 | 70 | 0.0056% | Rare | d__Bacteria; p__Firmicutes; c__Clostridia; o__Clostridiales; f__Clostridiaceae 1; g__Clostridium sensu stricto 1; s__uncultured bacterium |
|  | FJ682831.1.1371 | 74 | 817.84 | 73 | 0.0058% | Rare | d__Bacteria; p__Firmicutes; c__Clostridia; o__Clostridiales; f__Clostridiaceae 1; g__Clostridium sensu stricto 1; s__uncultured bacterium |
|  | HQ176105.1.1471 | 78 | 678.88 | 74 | 0.0059% | Rare | d__Bacteria; p__Firmicutes; c__Clostridia; o__Clostridiales; f__Clostridiaceae 1; g__Clostridium sensu stricto 1; s__uncultured bacterium |
|  | AM982567.1.1476 | 69 | 349.83 | 81 | 0.0064% | Rare | d__Bacteria; p__Firmicutes; c__Clostridia; o__Clostridiales; f__Clostridiaceae 1; g__Clostridium sensu stricto 1; s__uncultured bacterium |
|  | DQ796778.1.1374 | 87 | 2926.13 | 85 | 0.0068% | Rare | d__Bacteria; p__Firmicutes; c__Clostridia; o__Clostridiales; f__Clostridiaceae 1; g__Clostridium sensu stricto 1; s__uncultured bacterium |
|  | KJ808419.1.1480 | 72 | 435.62 | 100 | 0.0080% | Rare | d__Bacteria; p__Firmicutes; c__Clostridia; o__Clostridiales; f__Clostridiaceae 1; g__Clostridium sensu stricto 1; s__uncultured bacterium |
|  | EU473256.1.1382 | 77 | 623.11 | 105 | 0.0084% | Rare | d__Bacteria; p__Firmicutes; c__Clostridia; o__Clostridiales; f__Clostridiaceae 1; g__Clostridium sensu stricto 1; s__uncultured bacterium |
|  | GQ898504.1.1475 | 66 | 279.63 | 132 | 0.0105% | Moderate | d__Bacteria; p__Firmicutes; c__Clostridia; o__Clostridiales; f__Clostridiaceae 1; g__Clostridium sensu stricto 1; s__uncultured bacterium |
|  | HQ176032.1.1473 | 51 | 497.30 | 135 | 0.0107% | Moderate | d__Bacteria; p__Firmicutes; c__Clostridia; o__Clostridiales; f__Clostridiaceae 1; g__Clostridium sensu stricto 1; s__uncultured bacterium |
|  | HQ747801.1.1417 | 76 | 565.08 | 150 | 0.0119% | Moderate | d__Bacteria; p__Firmicutes; c__Clostridia; o__Clostridiales; f__Clostridiaceae 1; g__Clostridium sensu stricto 1; s__uncultured organism |
|  | FJ673641.1.1371 | 65 | 215.36 | 165 | 0.0131% | Moderate | d__Bacteria; p__Firmicutes; c__Clostridia; o__Clostridiales; f__Clostridiaceae 1; g__Clostridium sensu stricto 1; s__uncultured bacterium |
|  | HQ741797.1.1413 | 59 | 250.24 | 180 | 0.0143% | Moderate | d__Bacteria; p__Firmicutes; c__Clostridia; o__Clostridiales; f__Clostridiaceae 1; g__Clostridium sensu stricto 1; s__uncultured bacterium |
|  | HQ744264.1.1432 | 70 | 491.34 | 181 | 0.0144% | Moderate | d__Bacteria; p__Firmicutes; c__Clostridia; o__Clostridiales; f__Clostridiaceae 1; g__Clostridium sensu stricto 1; s__uncultured organism |
|  | DQ809413.1.1374 | 56 | 585.35 | 225 | 0.0179% | Moderate | d__Bacteria; p__Firmicutes; c__Clostridia; o__Clostridiales; f__Clostridiaceae 1; g__Clostridium sensu stricto 1; s__uncultured bacterium |
|  | JN210898.1.1382 | 58 | 961.36 | 366 | 0.0291% | Moderate | d__Bacteria; p__Proteobacteria; c__Alphaproteobacteria; o__Rhizobiales; f__Phyllobacteriaceae; g__Aliihoeflea; s__Aliihoeflea sp. S9 |
|  | HQ741515.1.1430 | 81 | 1315.73 | 392 | 0.0312% | Moderate | d__Bacteria; p__Firmicutes; c__Clostridia; o__Clostridiales; f__Clostridiaceae 1; g__Clostridium sensu stricto 1; s__uncultured bacterium |
|  | JUSY01000045.135.1671 | 59 | 640.67 | 1608 | 0.1280% | Abundant | d__Bacteria; p__Firmicutes; c__Bacilli; o__Lactobacillales; f__Streptococcaceae; g__Streptococcus; s__Streptococcus oligofermentans |
|  | JUTC01000041.4666.6201 | 58 | 674.38 | 3134 | 0.2494% | Abundant | d__Bacteria; p__Firmicutes; c__Bacilli; o__Lactobacillales; f__Streptococcaceae; g__Streptococcus; s__Streptococcus anginosus subsp. anginosus |

#### Table. S6

**Table. S6** Information on the composition of Phylum of rare taxa in GPCAP, GPMS and GPXXL.

| Group | Phylum | Number of OTUs | Proportion |
| --- | --- | --- | --- |
| GPCAP | Firmicutes | 106 | 47.75% |
|  | Proteobacteria | 74 | 33.33% |
|  | Bacteroidetes | 12 | 5.41% |
|  | Planctomycetes | 7 | 3.15% |
|  | Chloroflexi | 6 | 2.70% |
|  | Actinobacteria | 5 | 2.25% |
|  | Acidobacteria | 5 | 2.25% |
|  | Cyanobacteria | 3 | 1.35% |
|  | Gemmatimonadetes | 2 | 0.90% |
|  | Latescibacteria | 1 | 0.45% |
|  | Nitrospirae | 1 | 0.45% |
| GPMS | Proteobacteria | 236 | 61.14% |
|  | Firmicutes | 96 | 24.87% |
|  | Bacteroidetes | 42 | 10.88% |
|  | Actinobacteria | 5 | 1.30% |
|  | Cyanobacteria | 2 | 0.52% |
|  | Verrucomicrobia | 2 | 0.52% |
|  | Planctomycetes | 1 | 0.26% |
|  | Fibrobacteres | 1 | 0.26% |
|  | Acidobacteria | 1 | 0.26% |
| GPXXL | Proteobacteria | 266 | 55.42% |
|  | Firmicutes | 130 | 27.08% |
|  | Bacteroidetes | 53 | 11.04% |
|  | Actinobacteria | 15 | 3.13% |
|  | Planctomycetes | 6 | 1.25% |
|  | Cyanobacteria | 3 | 0.63% |
|  | Deinococcus-Thermus | 2 | 0.42% |
|  | Acidobacteria | 2 | 0.42% |
|  | Verrucomicrobia | 1 | 0.21% |
|  | Thermotogae | 1 | 0.21% |
|  | Fusobacteria | 1 | 0.21% |

#### Table. S7

**Table. S7** Information on the presence of the genus *Bacteriovorax* in all giant panda populations.

| **Group** | **OUT-ID** | **Module** | **Degree** | **Relative abundance** | **Species** | **Notes of importance** |
| --- | --- | --- | --- | --- | --- | --- |
| GPCAP | No related OTU |  |  |  |  |  |
| GPMS | AY294221.1.1465 | I | 104 | 0.1072% | *Bacteriovorax sp. PNEc1* | Working as connector in GP-network and as general node in GPMS-network |
|  | HM270587.1.1373 | I | 93 | 0.0281% | *uncultured bacterium* | Working as module hub in GP-network and as general node in GPMS-network |
|  | AJ288899.1.1371 | II | 57 | 0.0153% | *Bacteriovorax stolpii* | Working as peripheral node in GP-network and as keystone species in GPMS-network |
| GPXXL | HM270587.1.1373 | III | 40 | 0.0020% | *uncultured bacterium* | Working as module hub in GP-network and as general node in GPXXL-network |
